# Combined inorganic base promoted N-addition/[2,3]-sigmatropic rearrangement to construct homoallyl sulfur-containing pyrazolones†

**DOI:** 10.1039/c9ra07610g

**Published:** 2019-10-29

**Authors:** Shou-Jie Shen, Xiao-Li Du, Xiao-Li Xu, Yue-Hua Wu, Ming-gang Zhao, Jin-Yan Liang

**Affiliations:** Key Laboratory of Magnetic Molecules, Magnetic Information Materials Ministry of Education, The School of Chemical and Material Science, Shanxi Normal University Linfen 041004 China shoujie_shen@outlook.com jinyan_liang@outlook.com; College of Life Science, Shanxi Normal University Linfen 041004 China

## Abstract

The first sequentially combined inorganic base promoted N-addition/[2,3]-sigmatropic rearrangement reaction of α-alkylidene pyrazolinones and propargyl sulfonium salts has been reported to construct homoallyl sulfur-containing pyrazolones with moderate to excellent yields. α-Alkylidene pyrazolinones function as N-nucleophilic agents distinguished from the reported C-addition reactions. Propargyl sulfonium salts were first involved in the [2,3]-sigmatropic rearrangement protocol differentiated from the well-established annulation reactions. The excellent regioselectivity, the broad scope of substrates, gram-scale synthesis and convenient transformation embody the synthetic superiority of this cascade process.

## Abstract 2

The combined inorganic base promoted N-addition/[2,3]-sigmatropic rearrangement of α-alkylidene pyrazolinones and propargyl sulfonium salts has been reported to construct homoallyl sulfur-containing pyrazolones with moderate to excellent yields.

## Graphical abstract

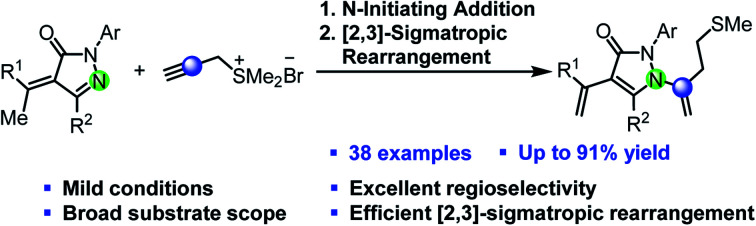

## Introduction

**Scheme 1:**
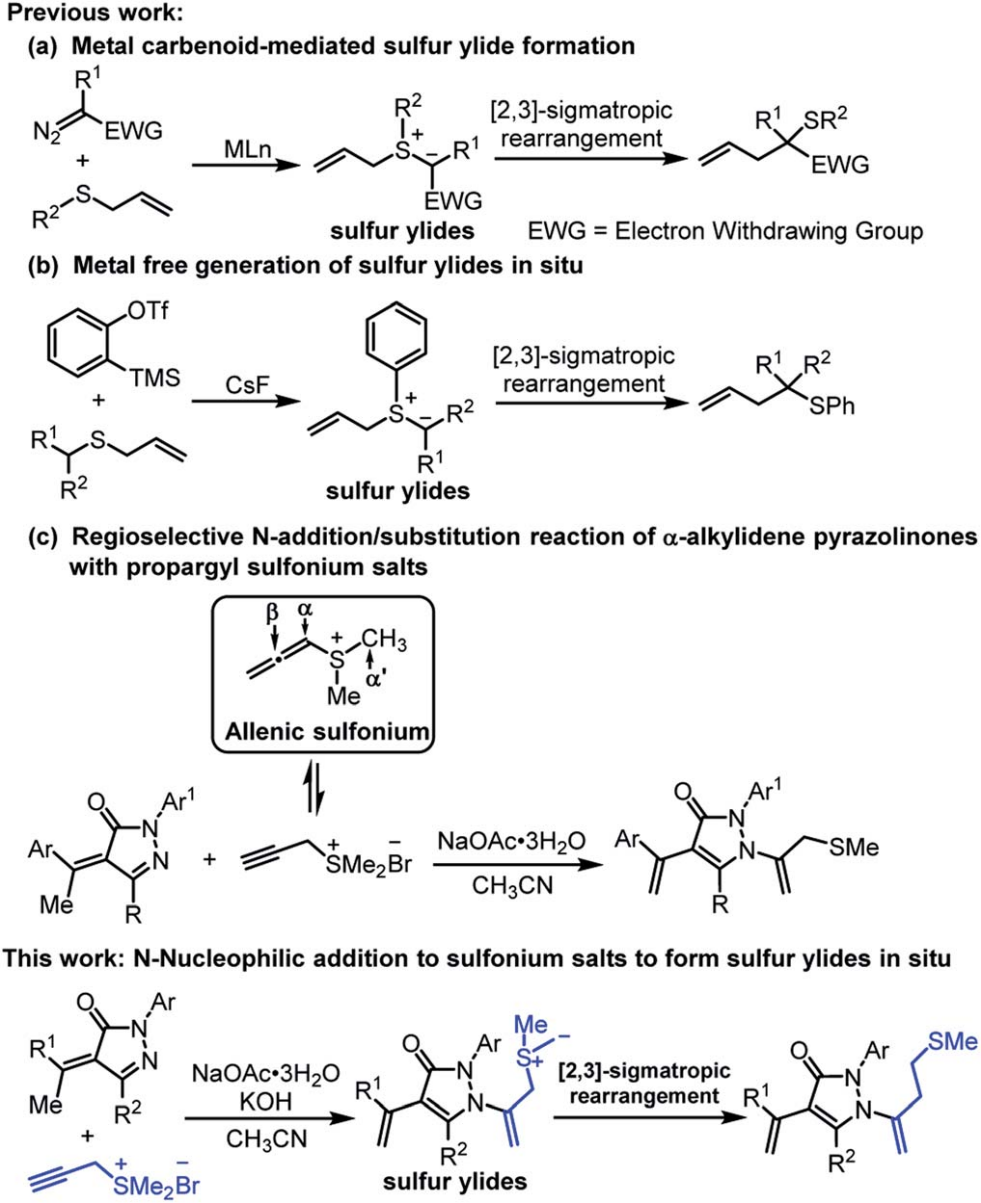
[2,3]-Sigmatropic rearrangements of sulfur ylides (a and b) and N-2 initiating nucleophilic reaction of α,β-unsaturated pyrazolone (c).

Organosulfur compounds that exist broadly in biologically active natural products as well as pharmaceuticals and are engaged in numerous chemical transformations, have been attracting vivid interest from academia and industry.^1^ Sulfur ylides are among the most versatile class of structural motifs, having widespread applications ranging from classical cyclopropanation, epoxidation and aziridination to more complicated [*n* + 1]-cycloadditions, domino reactions and rearrangement reactions based on transition metal catalysis, organocatalysis and photocatalysis.^2^ As an efficient C–C bond-forming strategy, [2,3]-sigmatropic rearrangements of sulfur ylides have been widely explored and applied since their discovery in the late 1960s.^3,4^ The transition metal carbenoid-mediated rearrangement reactions between allyl sulfides and diazo species named Doyle–Kirmse reactions are representative examples and have made impressive progress especially in to the content of catalytic and asymmetric variants in the past decade (Scheme 1a).^5^ Metal-free generation of sulfur ylides *in situ* between allyl thioethers and arynes followed by [2,3]-sigmatropic rearrangements are also alternative methods (Scheme 1b).^6^ Despite the aforementioned excellent work, the attempt to utilize propargyl sulfonium salts to form key species of sulfur ylides accompanied by subsequent [2,3]-sigmatropic rearrangements process has not been achieved.

Propargyl sulfonium salts, because of their easy acquirements and multiple reaction sites, are versatile and promising building blocks. Generally, propargyl sulfonium salts can isomerize to allenic sulfonium salts in the presence of the base, which are active forms and possess three reactive sites of α-carbon, β-carbon and α′-carbon (Scheme 1c). Kanematsu's,^7^ Huang's^8^ and our group^9^ have reported the [*n* + 2] or [*n* + 1] cascade annulation reactions based on α-carbon and β-carbon sites. Meanwhile, pyrazolones represent a class of privileged heterocycles that exhibit extensive physiological and pharmacological activities and are valuable drug candidates.^10^ Accordingly, fruitful protocols have been explored to access versatile pyrazolones architectures based on the multiple reactive sites of pyrazolin-5-ones and α,β-unsaturated pyrazolones.^11,12^ As to α,β-unsaturated pyrazolones having γ-H, the preferential γ-C nucleophilic property facilities their functioning as C3 synthons to construct spiro-pyrazolones by [3 + *n*] annulation.^13^ In contrast, the N-2 initiating nucleophilic reaction of α,β-unsaturated pyrazolones was less investigated. Recently our group reported the first regioselective NaOAc·3H_2_O-promoted N-addition/substitution reaction between α-alkylidene pyrazolinones and propargyl sulfonium salts (Scheme 1c).^14^ Accidentally, we found that strong inorganic bases can efficiently promoted the rearrangement of sulfur salts. Based on our processive interests on constructing functionalized pyrazolones and exploring the diverse reactive pathway of propargyl sulfonium salts,^9,15^ we herein report the realization of regioselective NaOAc·3H_2_O/KOH-promoted N-addition/[2,3]-sigmatropic rearrangement reaction of α-alkylidene pyrazolinones and propargyl sulfonium salts, delivering bioactive homoallyl sulfur-containing pyrazolones in moderate to excellent yields (Scheme 1d).

## Results and discussion

We began our investigation by selecting α-alkylidene pyrazolinone 1a and propargyl sulfonium salt 2a as model substrates (Table 1). When 1a (0.2 mmol, 1.0 equiv.), 2a (0.24 mmol, 1.2 equiv.) and NaOAc·3H_2_O (0.1 mmol, 0.5 equiv.) in CH_3_CN (2 mL) were mixed and stirred for 10 minutes at 20 °C, the α-alkylidene pyrazolinone 1a was consumed completely. After the reaction temperature was decreased to 0 °C, KOH (0.4 mmol, 2.0 equiv.) was added and the reaction was stirred for 6 h at 0 °C to afford N-addition/[2,3]-sigmatropic rearrangement product 3a with 82% yield (Table 1, entry 4). When the mixture of NaOAc·3H_2_O and KOH was added in one portion, α-alkylidene pyrazolinone could not be consumed completely even after 12 h and the operation caused decreased yield. Extensive exploration of a range of bases indicated that NaOAc·3H_2_O or anhydrous NaOAc was most efficient to promote the process (entries 4–5 *vs.* 1–3 and 6–10 in Table 1). The combined usage of bases NaOAc·3H_2_O and KOH was crucial for the high efficiency while NaOAc·3H_2_O or KOH was utilized separately to give the desired 3a in 0% and 46% yields, respectively (Table 1, entries 11–12). Replacement of KOH with NaOH or LiOH gave related 74% and 68% yields (Table 1, entries 13–14). Screening of solvents including CH_2_Cl_2_, CHCl_3_, MeOH, THF and toluene did not give a better yield (entries 4 *vs.* 15–19). We also probed the influence of the amount of sulfonium salts 2a, NaOAc·3H_2_O and KOH. Increasing the content of propargyl sulfonium salt 2a to 2.0 equiv. has little influence on yield (entry 20). Decreasing the amount of NaOAc·3H_2_O to 0.25 equiv. or increasing to 1.0 equiv. had a slight effect on yield (entries 21–22). Increasing the quantities of KOH to 4.0 equiv. did not improve the yield (entry 23). The yield was declined from 82% to 78% when the reaction was stirred at 20 °C (entry 24).

**Table 1:** Optimization of the reaction conditions^a^

Entry	Base1/base2	Solvent	1a : 2a : base1 : base2	Yield^b^ (%)
1	Na_2_CO_3_/KOH	CH_3_CN	1 : 1.2 : 0.5 : 2	69
2	K_2_CO_3_/KOH	CH_3_CN	1 : 1.2 : 0.5 : 2	58
3	Cs_2_CO_3_/KOH	CH_3_CN	1 : 1.2 : 0.5 : 2	75
**4**	**NaOAc·3H**_**2**_ **O/KOH**	**CH**_**3**_ **CN**	**1** **:** **1.2** **:** **0.5** **:** **2**	**82**
5	NaOAc/KOH	CH_3_CN	1 : 1.2 : 0.5 : 2	82
6	KOAc/KOH	CH_3_CN	1 : 1.2 : 0.5 : 2	68
7	NEt_3_/KOH	CH_3_CN	1 : 1.2 : 0.5 : 2	39
8	DABCO^*c*^/KOH	CH_3_CN	1 : 1.2 : 0.5 : 2	68
9	DBU^*d*^/KOH	CH_3_CN	1 : 1.2 : 0.5 : 2	60
10	DMAP^*e*^/KOH	CH_3_CN	1 : 1.2 : 0.5 : 2	75
11	NaOAc·3H_2_O	CH_3_CN	1 : 1.2 : 0.5 : 0	0
12	KOH	CH_3_CN	1 : 1.2 : 0 : 2	46
13	NaOAc·3H_2_O/NaOH	CH_3_CN	1 : 1.2 : 0.5 : 2	74
14	NaOAc·3H_2_O/LiOH	CH_3_CN	1 : 1.2 : 0.5 : 2	68
15	NaOAc·3H_2_O/KOH	CH_2_CI_2_	1 : 1.2 : 0.5 : 2	74
16	NaOAc·3H_2_O/KOH	CHCI_3_	1 : 1.2 : 0.5 : 2	53
17	NaOAc·3H_2_O/KOH	MeOH	1 : 1.2 : 0.5 : 2	64
18	NaOAc·3H_2_O/KOH	THF	1 : 1.2 : 0.5 : 2	48
19	NaOAc·3H_2_O/KOH	Toluene	1 : 1.2 : 0.5 : 2	36
20	NaOAc·3H_2_O/KOH	CH_3_CN	1 : 2.0 : 0.5 : 2	83
21	NaOAc·3H_2_O/KOH	CH_3_CN	1 : 1.2 : 0.25 : 2	80
22	NaOAc·3H_2_O/KOH	CH_3_CN	1 : 1.2 : 1.0 : 2	82
23	NaOAc·3H_2_O/KOH	CH_3_CN	1 : 1.2 : 0.5 : 4	81
24	NaOAc·3H_2_O/KOH	CH_3_CN	1 : 1.2 : 0.5 : 2	78

a Unless otherwise noted, the reactions were performed under air and α-alkylidene pyrazolinones 1 (0.2 mmol, 1.0 equiv.), sulfonium salt 2a (0.24 mmol, 1.2 equiv.) and NaOAc·3H_2_O (0.1 mmol, 0.5 equiv.) in CH_3_CN (2.0 mL) were mixed and stirred for 10–40 minutes at 20 °C until starting material 1a disappeared (monitored by TLC), then the reaction temperature was decreased to 0 °C and KOH (2.0 equiv.) was added to keep stirring at 0 °C for 6–10 h.

b Isolated yield.

**Scheme 2:**
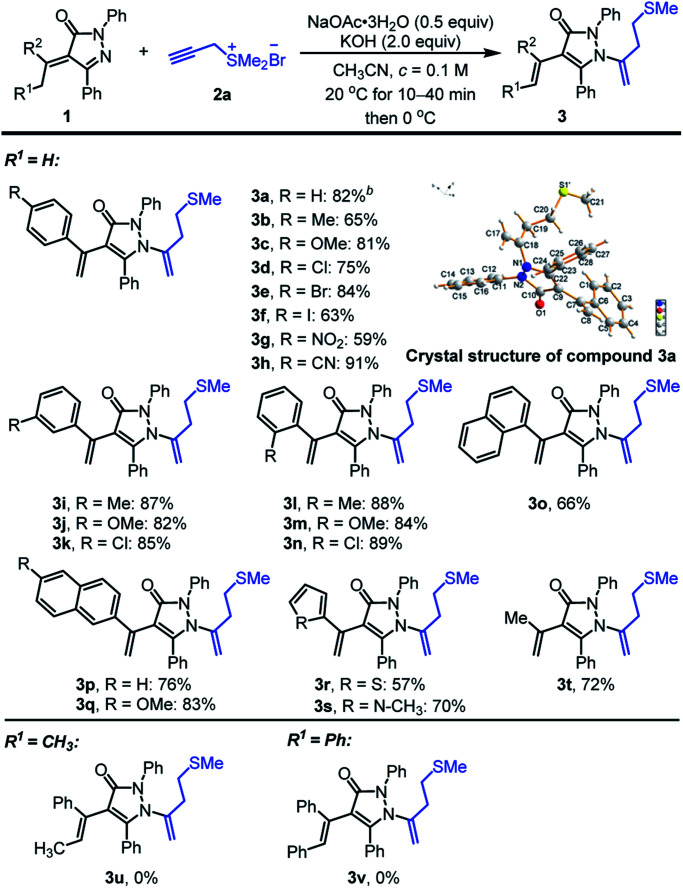
Scope of Disubstituted α-Alkylidene Pyrazolinones.^*a*^ Unless otherwise noted, the reactions were performed under air and α-alkylidene pyrazolinones 1 (0.2 mmol, 1.0 equiv.), sulfonium salt 2a (0.24 mmol, 1.2 equiv.) and NaOAc·3H_2_O (0.1 mmol, 0.5 equiv.) in CH_3_CN (2.0 mL) were mixed, stirred for 10–40 minutes at 20 °C until starting material 1a disappeared (monitored by TLC), then the reaction temperature was decreased to 0 °C and KOH (2.0 equiv.) was added to keep stirring at 0 °C for 6–10 h.^*b*^ Isolated yield.

Having established the optimized conditions (Table 1, entry 4), we commenced to explore the substrate scope of the reaction (Scheme 2). Generally, the existence of methyl group (R^1^ = H) at α-position of alkylidene pyrazolinones was pivotal for the success of the reaction and various aryl and alkyl-substituted alkylidene pyrazolinones 1 was adaptable to the transformation. Acetophenones derived alkylidene pyrazolinones 1 with methyl, methoxy, chloro-, bromo-, iodo-, nitro- and cyano-groups on *ortho*-, *meta*- or *para*-positions, could react effectively with propargyl sulfonium salt 2a to furnish the related homoallyl sulfur-containing pyrazolones in 59–91% yields (3a–3n in Scheme 2). As to the same substituent on phenyl group, such as methyl, methoxy and chloro-, *ortho*- and *meta*-positions exhibited higher yields than *para*-position (3b*vs.*3i, 3l; 3c*vs.*3j, 3m; 3d*vs.*3k, 3n). Naphthyl-substituted α-alkylidene pyrazolinones were well-tolerated to provide 3o, 3p and 3q in 66, 76 and 83% yields, respectively. Hetero-aromatic unsaturated pyrazolinones containing thiophene and N-methyl protected pyrrole could participate in the reaction to afford corresponding product 3r and 3s in 57 and 70% yields, respectively. Double alkyl-substituted alkylidene pyrazolinone could also be engaged in the reaction to produce the predicted 3t with 72% yield. In contrast, when the methyl group was replaced by ethyl (R^1^ = CH_3_) or benzyl groups (R^1^ = Ph), the desired reaction was sluggish, abundant alkylidene pyrazolinones were recovered and no target products 3u or 3v could be separated. In addition, the structure of homoallyl sulfur-containing pyrazolone derivative 3a was assigned unambiguously by using single crystal X-ray analysis.^16^

**Scheme 3:**
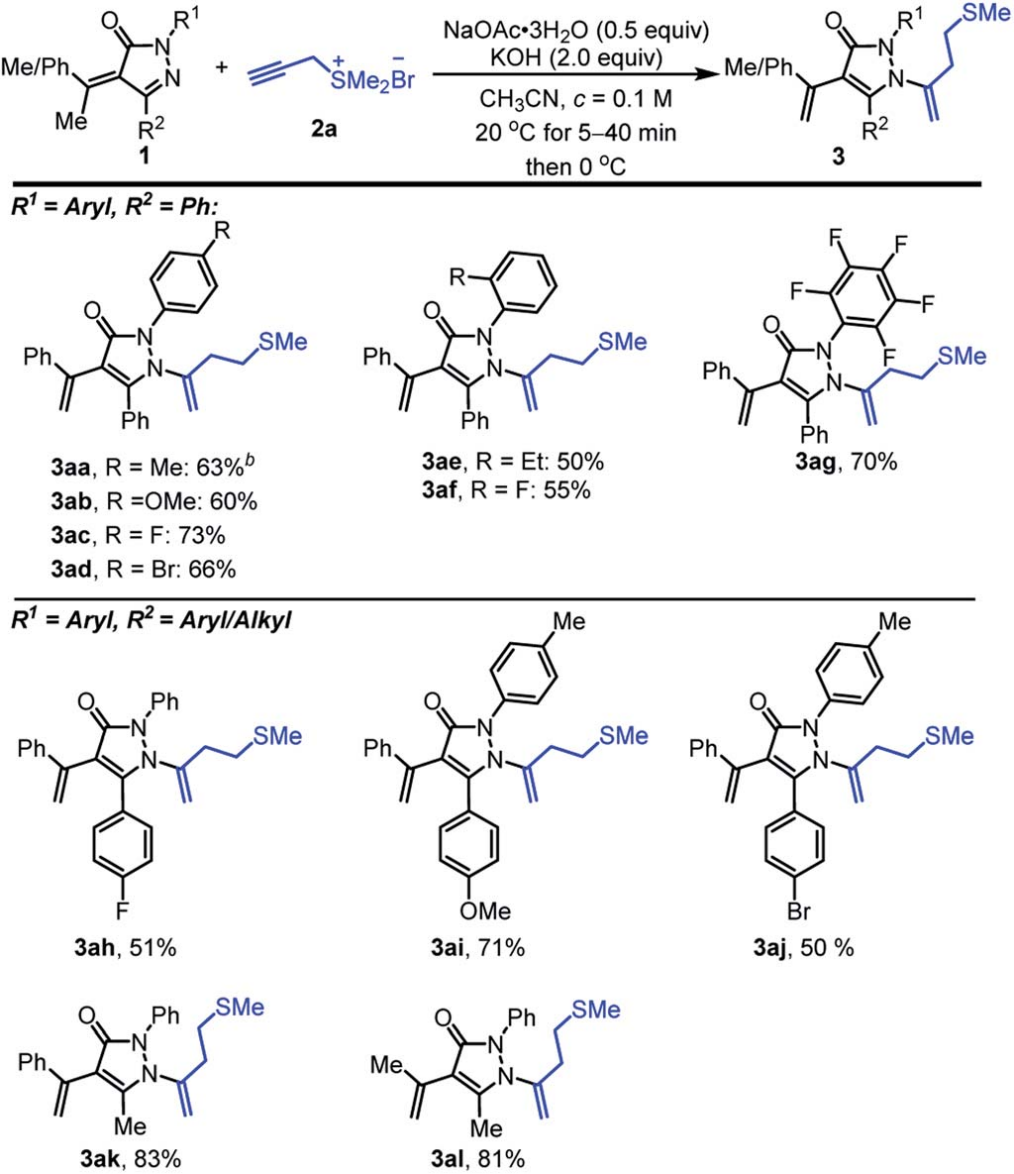
Scope of α-alkylidene pyrazolinones on the pyrazolinone ring.^*a*^ Unless otherwise noted, the reactions were performed under air and α-alkylidene pyrazolinones 1 (0.2 mmol, 1.0 equiv.), sulfonium salt 2a (0.24 mmol, 1.2 equiv.) and NaOAc·3H_2_O (0.1 mmol, 0.5 equiv.) in CH_3_CN (2.0 mL) were mixed and stirred for 10–40 minutes at 20 °C until starting material 1 disappeared (monitored by TLC), then the reaction temperature was decreased to 0 °C and KOH (2.0 equiv.) was added to keep stirring at 0 °C for 6–10 h. ^*b*^Isolated yield.

Subsequently, we went on to evaluate the effect of different substituents on pyrazolinone ring (Scheme 3). α-Alkylidene pyrazolinones 1 with the *para*-substituted phenyl-ring of R^1^ worked well to deliver the corresponding products 3aa, 3ab, 3ac and 3ad with moderate yields of 63, 60, 73 and 66%, respectively. Electron-withdrawing groups on the phenyl group gave better yields than electron-donating groups (3ac, 3ad*vs.*3aa, 3ab). *ortho*-Ethyl, fluoro-substituted phenyl ring of R^1^ gave relatively lower yields of 50 and 55% partially because of the instability of 1. It is noteworthy that the substrate having electron-withdrawing group C_6_F_5_- supplied the desired product 3ag with a moderate yield of 70%. α-Alkylidene pyrazolinones 1 with 4-fluoro-, 4-methoxy and 4-bromo-substituted phenyl ring of R^2^ could also be applied to the reaction and provide the related homoallyl sulfur-containing pyrazolones 3ah, 3ai and 3aj with 51, 71 and 50% yields. α-Alkylidene pyrazolinones 1 including alkyl group of R^2^ showed excellent compatibility and afforded 3ak with 83% yield. Moreover, trimethyl involved alkylidene pyrazolinone displayed proof of tolerance and 81% yield was obtained (3al).

**Scheme 4:**
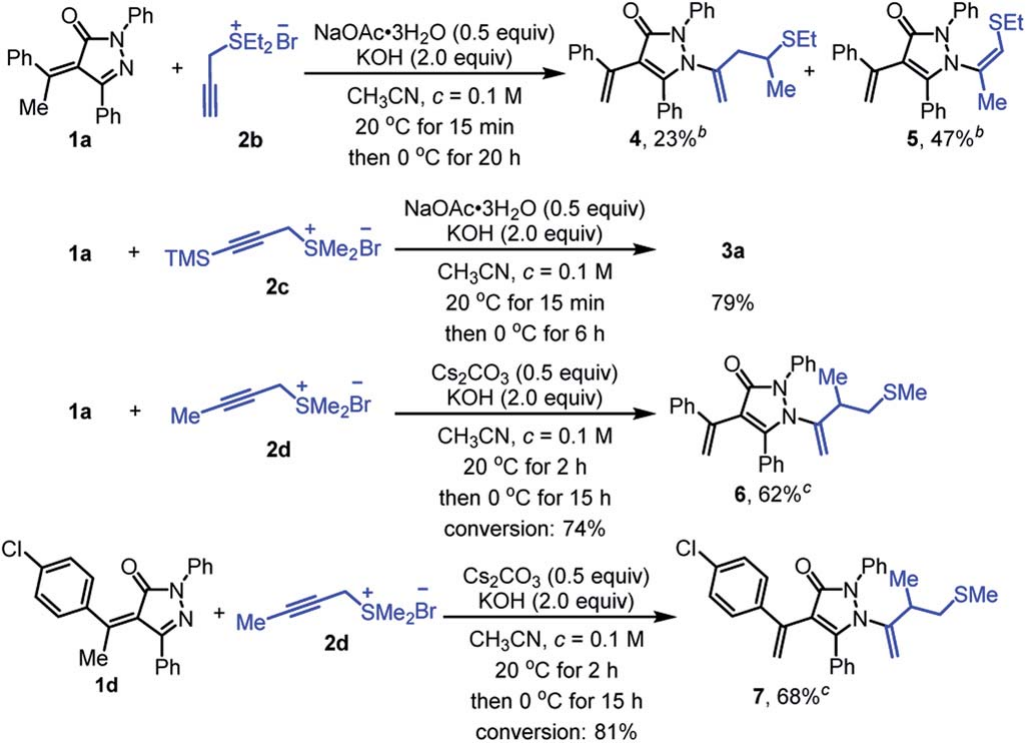
Scope of propargyl sulfonium salts.^*a*^ Unless otherwise noted, the reactions were performed under air and α-alkylidene pyrazolinone 1a or 1d (0.2 mmol, 1.0 equiv.), sulfonium salts 2b, 2c or 2d (0.24 mmol, 1.2 equiv.) and NaOAc·3H_2_O (0.1 mmol, 0.5 equiv.) in CH_3_CN (2.0 mL) were mixed and stirred at 20 °C until starting material 1a or 1d disappeared (monitored by TLC), then the reaction temperature was decreased to 0 °C and KOH (2.0 equiv.) was added to keep stirring at 0 °C for related time. ^*b*^Isolated yield.^*c*^ Cs_2_CO_3_ (0.1 mmol, 0.5 equiv.) was utilized instead of NaOAc·3H_2_O under the standard condition and the reaction was kept stirring at 0 °C for 15 h.

To further broaden the scope of the reaction, other representative propargyl sulfonium salts were also investigated (Scheme 4). Diethyl thioether derived propargyl sulfonium salt 2b was adaptable to give the predicted homoallyl sulfur-containing pyrazolone 4 in 23% yield, together with additional isomerization product 5 in 47% yield. Trimethylsilyl-containing propargyl sulfonium salt 2c can also be applied to the reaction but the desilylation product 3a was obtained with a yield of 79%. Methyl substituted propargyl sulfonium salt 2d did not engage in the reaction under standard condition, mainly because NaOAc·3H_2_O was not suitable to transform propargyl sulfonium salt 2d into active allenic form and alkylidene pyrazolinone 1a was nearly fully recovered after stirred at 20 °C for 20 h. When NaOAc·3H_2_O was replaced by Cs_2_CO_3_, the reaction could proceed to afford the desired product 6 with 62% yield. Substrate 1d could also react with 2d smoothly to provide 7 with 68% yield under the same conditions.

**Scheme 5:**
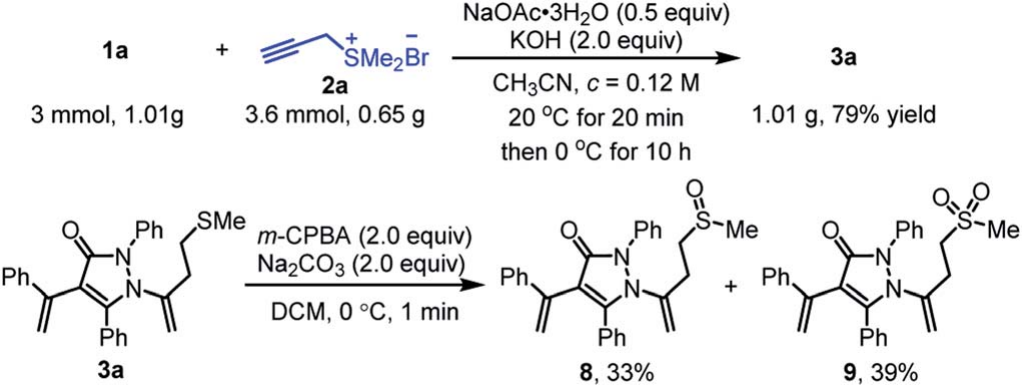
Gram-scale synthesis and further transformation of 3a.

To demonstrate the further synthetic utility of this protocol, we performed the large-scale operation using α-alkylidene pyrazolinone 1a (1.01 g, 3 mmol) and propargyl sulfonium salt 2a (1.2 equiv.) as the representative substrates under the optimized conditions, providing the related product 3a (1.00 g) with 79% yield (Scheme 5). The typical transformation was also conducted by oxidation of 3a with *m*-chloro peroxybenzoic acid (2.0 equiv.), sulfinyl product 8 and sulfonyl product 9 were obtained in Scheme 5 with 33% and 39% yields, respectively.

**Scheme 6:**
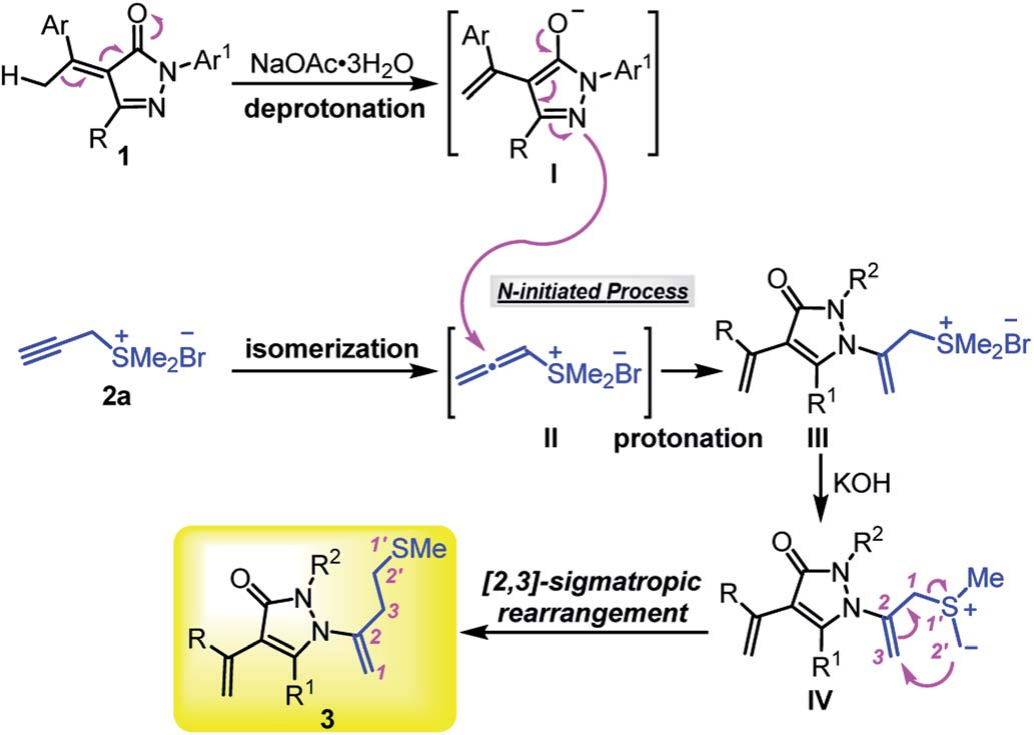
Plausible reaction mechanism.

According to the experimental observations and previous reports,^8,9,14^ a possible mechanism is proposed to account for the formation of homoallyl sulfur-containing pyrazolone derivatives 3 (Scheme 6). Under the activation of inorganic base NaOAc·3H_2_O, α-alkylidene pyrazolone 1 can form intermediate I and propargyl sulfonium salt 2a can isomerize to allenic sulfonium salts II. The N-nucleophilic attack of I to allenic sulfonium salts II initiates the reaction and gives intermediate III after protonation. Subsequently, the deprotonation of methyl-carbon by KOH provides the key sulfur ylide IV. Finally, the [2,3]-sigmatropic rearrangement of key species IV affords the desired product 3.

## Conclusions

In summary, we have developed a sequentially combined inorganic bases promoted N-addition/[2,3]-sigmatropic rearrangement reaction between α-alkylidene pyrazolinones and propargyl sulfonium salts for the first time, delivering bioactive homoallyl sulfur-containing pyrazolones in moderate to excellent yields. In this reaction, α-alkylidene pyrazolinones function as N-nucleophilic agents distinguished from reported C-addition reactions. Meanwhile, propargyl sulfonium salts were first involved in [2,3]-sigmatropic rearrangement protocols differentiated from the well-established annulation reactions. Gram-scale synthesis and convenient transformations are furnished. The proposed mechanism is also discussed. Excellent regioselectivity, the broad scope of substrates, gram-scale synthesis and convenient transformation embody the synthetic superiority of this reaction process.

## Experimental

### General information

All reactions were performed in oven-dried or flame-dried round-bottom flasks and vials. Stainless steel syringes and cannula were used to transfer air- and moisture-sensitive liquids. Flash chromatography was performed using silica gel 60 (230–400 mesh) from Aladdin. Commercial reagents were purchased from Aladdin, J&K, Macklin and Meryer and used as received. All solvents were used after being freshly distilled unless otherwise noted. Proton nuclear magnetic resonance (^1^H NMR) spectra and carbon nuclear magnetic resonance (^13^C NMR) spectra were recorded on Bruker UltraShield-600 (600 MHz). The mass spectroscopic data were obtained using a Micromass Platform II single quadrupole instrument. Infrared (IR) spectra were obtained using a PerkinElmer Spectrum 100 FT-IR spectrometer.

### General procedure for the synthesis of α-alkylidene pyrazolinones (1)

α-Alkylidene pyrazolinones 1 were prepared through a known procedure:^12*b*,13*b*,14,17^ aryl formyl acetate (5.5 mmol, 1.1 equiv.) was slowly added to the mixture of the corresponding hydrazine (5 mmol, 1.0 equiv.) in glacial acetic acid (2 mL). The mixture was stirred at room temperature for 24 h. NEt_3_ (5 mmol, 1.0 equiv.) was added to neutralize the hydrochloride while phenylhydrazine hydrochloride was used. After the reaction was completed, ethyl (50 mL) was added. The precipitate was filtered and washed with 5 mL of ether (three times). The corresponding pyrozolone products were obtained as solid and used in the following step.

Under nitrogen atmosphere, a mixture of pyrazolone (5 mmol, 1.0 equiv.), acetophenone (6 mmol, 1.2 equiv.) and pyridine (0.8 mL, 10 mmol) in THF (10 mL) was stirred for 10 min followed by slow addition (30 min) of Titanium isopropoxide (4.3 mL, 15 mmol). The mixture was stirred at room temperature for 24 h. The resulting reaction mixture was diluted with EtOAc (100 mL) and washed with 1 N aqueous HCl, saturated aqueous solution of NaHCO_3_ and brine. The organic layer was dried over Na_2_SO_4_, concentrated, and purified by column chromatography to provide α-alkylidene pyrazolinone derivatives 1. If the products are mixed with excess liquid acetophenes, they can be further purified by washing with petroleum ether.

Propargyl Sulfonium Salts (2a, 2b, 2c, 2d and 2e) were prepared through a known procedure.^18^

### General procedure for the reaction of unsaturated pyrazolones with propargyl sulfide ylide

To a flame-dried sealable 3-dram vial equipped with a stir bar was added unsaturated pyrazolinones 1 (0.2 mmol, 1.0 equiv.), NaOAc·3H_2_O (0.1 mmol, 0.5 equiv.) and 2a (44 mg, 0.24 mmol, 1.2 equiv.) under air. Subsequently treated CH_3_CN (2 mL, *c* = 0.1 M) was added to vial *via* syringe. The reaction mixture was stirred for 10–40 min at 20 °C until unsaturated pyrazolones 1 was fully consumed (monitored by TLC). Then the reaction was stirred at 0 °C for 6–10 h. The organic solvent was removed under reduced pressure and purified through column chromatography (eluent: petroleum ether and EtOAc) to afford the desired product 3.

### 1-(4-(Methylthio)but-1-*en*-2-yl)-2,5-diphenyl-4-(1-phenylvinyl)-1,2-dihydro-3*H*-pyrazol-3-one (3a)

The reaction was carried out on a 0.2 mmol scale following the General procedure. The product 3a was purified through column chromatography (PE/EtOAc: from 6 : 1 to 4 : 1) and obtained as a white solid (75 mg, 85% yield). mp 174.7–175.6 °C. IR *ν*_max_ (neat)/cm^−1^: 3062, 2985, 1677, 1568, 1452, 1391, 1183, 1026, 955; ^1^H NMR (600 MHz, CDCl_3_): *δ* 7.62 (dd, *J* = 1.2, 7.8 Hz, 2H), 7.47 (t, *J* = 7.8 Hz, 2H), 7.34–7.29 (m, 5H), 7.26 (d, *J* = 7.2 Hz, 1H), 7.23 (t, *J* = 7.2 Hz, 2H), 7.17 (t, *J* = 7.8 Hz, 2H), 7.13 (t, *J* = 7.2 Hz, 1H), 5.69 (d, *J* = 1.2 Hz, 1H), 5.65 (d, *J* = 1.2 Hz, 1H), 5.45 (s, 1H), 5.06 (s, 1H), 2.24 (t, *J* = 7.8 Hz, 2H), 2.05 (t, *J* = 7.8 Hz, 2H), 1.94 (s, 3H); ^13^C{^1^H} NMR (150 MHz, CDCl_3_): *δ* 164.6, 155.6, 145.1, 140.2, 138.3, 135.9, 130.0, 129.6, 129.2, 128.7, 127.9, 127.9, 127.2, 126.9, 126.4, 124.2, 118.9, 117.9, 114.3, 31.3, 31.1, 15.3. HRMS (ESI-TOF, *m*/*z*): calcd for C_28_H_26_N_2_NaOS^+^, [M + Na]^+^, 461.1658, found 461.1662.

### 1-(4-(Methylthio)but-1-*en*-2-yl)-2,5-diphenyl-4-(1-(*p*-tolyl)vinyl)-1,2-dihydro-3*H*-pyrazol-3-one (3b)

The reaction was carried out on a 0.2 mmol scale following the General procedure. The product 3b was purified through column chromatography (PE/EtOAc: from 6 : 1 to 4 : 1) and obtained as a foam solid (59 mg, 65% yield). IR *ν*_max_ (neat)/cm^−1^: 3102, 2899, 1698, 1571, 1433, 1367, 1256, 1142, 1030, 979; ^1^H NMR (600 MHz, CDCl_3_): *δ* 7.62 (dd, *J* = 0.6, 8.4 Hz, 2H), 7.46 (t, *J* = 7.8 Hz, 2H), 7.36 (d, *J* = 8.4 Hz, 2H), 7.31–7.30 (m, 2H), 7.28–7.23 (m, 4H), 7.00 (d, *J* = 7.8 Hz, 2H), 5.64 (d, *J* = 1.2 Hz, 1H), 5.49 (d, *J* = 1.2 Hz, 1H), 5.44 (s, 1H), 5.06 (s, 1H), 2.28 (s, 3H), 2.26 (t, *J* = 7.8 Hz, 2H), 2.06 (t, *J* = 7.8 Hz, 2H), 1.95 (s, 3H); ^13^C{^1^H} NMR (150 MHz, CDCl_3_): *δ* 164.7, 155.6, 145.3, 138.2, 137.4, 137.0, 136.1, 130.1, 129.6, 129.4, 128.7, 127.9, 126.8, 126.4, 124.1, 118.0, 117.8, 114.8, 31.4, 31.1, 21.0, 15.4. HRMS (ESI-TOF, *m*/*z*): calcd for C_29_H_28_N_2_ONaS^+^, [M + Na]^+^, 475.1815, found 475.1812.

### 4-(1-(4-Methoxyphenyl)vinyl)-1-(4-(methylthio)but-1-*en*-2-yl)-2,5-diphenyl-1,2-dihydro-3*H*-pyrazol-3-one (3c)

The reaction was carried out on a 0.2 mmol scale following the General procedure. The product 3c was purified through column chromatography (PE/EtOAc: from 6 : 1 to 4 : 1) and obtained as a foam solid (76 mg, 81% yield). IR *ν*_max_ (neat)/cm^−1^: 3087, 2977, 1664, 1582, 1456, 1361, 1129, 1057, 933, 854; ^1^H NMR (600 MHz, CDCl_3_): *δ* 7.62 (d, *J* = 7.2 Hz, 2H), 7.46 (t, *J* = 7.8 Hz, 2H), 7.36 (d, *J* = 7.2 Hz, 2H), 7.30–7.24 (m, 6H), 6.72 (d, *J* = 9.0 Hz, 2H), 5.60 (d, *J* = 1.2 Hz, 1H), 5.48 (d, *J* = 1.2 Hz, 1H), 5.45 (s, 1H), 5.06 (s, 1H), 3.75 (s, 3H), 2.24 (t, *J* = 7.8 Hz, 2H), 2.05 (t, *J* = 7.8 Hz, 2H), 1.94 (s, 3H); ^13^C{^1^H} NMR (150 MHz, CDCl_3_): *δ* 164.5, 159.1, 155.5, 145.1, 137.6, 135.9, 132.8, 129.9, 129.5, 129.2, 128.6, 127.9, 127.8, 126.3, 124.0, 117.8, 117.1, 114.6, 113.5, 55.2, 31.3, 31.0, 15.3. HRMS (ESI-TOF, *m*/*z*): calcd for C_29_H_29_N_2_O_2_S^+^, [M + H]^+^, 469.1944, found 469.1943.

### 4-(1-(4-Chlorophenyl)vinyl)-1-(4-(methylthio)but-1-*en*-2-yl)-2,5-diphenyl-1,2-dihydro-3*H*-pyrazol-3-one (3d)

The reaction was carried out on a 0.2 mmol scale following the General procedure. The product 3d was purified through column chromatography (PE/EtOAc: from 6 : 1 to 4 : 1) and obtained as a foam solid (71 mg, 75% yield). IR *ν*_max_ (neat)/cm^−1^: 3073, 2956, 1680, 1479, 1364, 1182, 1036, 937; ^1^H NMR (600 MHz, CDCl_3_): *δ* 7.60 (dd, *J* = 1.2, 8.4 Hz, 2H), 7.47 (t, *J* = 8.4 Hz, 2H), 7.30 (d, *J* = 8.4 Hz, 4H), 7.25 (d, *J* = 7.8 Hz, 2H), 7.23–7.21 (m, 2H), 7.11 (d, *J* = 8.4 Hz, 2H), 5.69 (d, *J* = 1.2 Hz, 1H), 5.65 (d, *J* = 1.2 Hz, 1H), 5.44 (s, 1H), 5.06 (s, 1H), 2.23 (t, *J* = 7.8 Hz, 2H), 2.04 (t, *J* = 7.8 Hz, 2H), 1.93 (s, 3H); ^13^C{^1^H} NMR (150 MHz, CDCl_3_): *δ* 164.3, 155.5, 144.9, 138.8, 137.3, 135.7, 133.1, 130.0, 129.7, 129.1, 128.8, 128.2, 128.0, 128.0, 126.5, 124.2, 119.3, 118.0, 113.6, 31.2, 15.3. HRMS (ESI-TOF, *m*/*z*): calcd for C_28_H_26_ClN_2_OS^+^, [M + H]^+^, 473.1449, found 473.1451.

### 4-(1-(4-Bromophenyl)vinyl)-1-(4-(methylthio)but-1-*en*-2-yl)-2,5-diphenyl-1,2-dihydro-3*H*-pyrazol-3-one (3e)

The reaction was carried out on a 0.2 mmol scale following the General procedure. The product 3e was purified through column chromatography (PE/EtOAc: from 6 : 1 to 4 : 1) and obtained as a foam solid (87 mg, 84% yield). IR *ν*_max_ (neat)/cm^−1^: 3102, 2941, 1668, 1603, 1490, 1377, 1268, 1146, 1021, 919, 832; ^1^H NMR (600 MHz, CDCl_3_): *δ* 7.60 (d, *J* = 7.8 Hz, 2H), 7.46 (t, *J* = 7.8 Hz, 2H), 7.31–7.26 (m, 5H), 7.25–7.22 (m, 3H), 7.15 (d, *J* = 8.4 Hz, 2H), 5.69 (d, *J* = 0.6 Hz, 1H), 5.64 (d, *J* = 0.6 Hz, 1H), 5.42 (s, 1H), 5.05 (s, 1H), 2.23 (t, *J* = 7.8 Hz, 2H), 2.04 (t, *J* = 7.8 Hz, 2H), 1.93 (s, 3H); ^13^C{^1^H} NMR (150 MHz, CDCl_3_): *δ* 164.3, 155.4, 144.8, 139.3, 137.3, 135.7, 131.0, 129.9, 129.7, 129.0, 128.7, 128.5, 128.0, 126.5, 124.1, 121.2, 119.2, 118.0, 113.4, 31.2, 15.3. HRMS (ESI-TOF, *m*/*z*): calcd for C_28_H_26_BrN_2_OS^+^, [M + H]^+^, 517.0944, found 517.0945.

### 4-(1-(4-Iodophenyl)vinyl)-1-(4-(methylthio)but-1-*en*-2-yl)-2,5-diphenyl-1,2-dihydro-3*H*-pyrazol-3-one (3f)

The reaction was carried out on a 0.2 mmol scale following the General procedure. The product 3f was purified through column chromatography (PE/EtOAc: from 6 : 1 to 4 : 1) and obtained as a foam solid (71 mg, 63% yield). IR *ν*_max_ (neat)/cm^−1^: 3082, 2986, 1677, 1598, 1465, 1343, 1188, 1067, 962; ^1^H NMR (600 MHz, CDCl_3_): *δ* 7.60 (dd, *J* = 1.2, 7.8 Hz, 2H), 7.48–7.45 (m, 4H), 7.32–7.28 (m, 4H), 7.25 (d, *J* = 7.2 Hz, 2H), 7.03 (d, *J* = 8.4 Hz, 2H), 5.67 (d, *J* = 1.2 Hz, 1H), 5.64 (d, *J* = 1.2 Hz, 1H), 5.42 (s, 1H), 5.06 (s, 1H), 2.23 (t, *J* = 7.8 Hz, 2H), 2.04 (t, *J* = 7.8 Hz, 2H), 1.93 (s, 3H); ^13^C{^1^H} NMR (150 MHz, CDCl_3_): *δ* 164.3, 155.5, 144.9, 139.9, 137.5, 137.0, 135.8, 130.0, 129.7, 129.1, 128.8, 128.7, 128.0, 126.5, 124.2, 119.3, 117.9, 113.4, 92.7, 31.2, 15.3. HRMS (ESI-TOF, *m*/*z*): calcd for C_28_H_25_IN_2_ONaS^+^, [M + Na]^+^, 587.0624, found 587.0620.

### 1-(4-(Methylthio)but-1-*en*-2-yl)-4-(1-(4-nitrophenyl)vinyl)-2,5-diphenyl-1,2-dihydro-3*H*-pyrazol-3-one (3g)

The reaction was carried out on a 0.2 mmol scale following the General procedure. The product 3g was purified through column chromatography (PE/EtOAc: from 6 : 1 to 4 : 1) and obtained as a foam solid (57 mg, 59% yield). IR *ν*_max_ (neat)/cm^−1^: 3076, 2977, 1668, 1599, 1472, 1338, 1169, 1027, 912, 835; ^1^H NMR (600 MHz, CDCl_3_): *δ* 7.98 (d, *J* = 9.0 Hz, 2H), 7.60 (dd, *J* = 1.2, 8.4 Hz, 2H), 7.49 (t, *J* = 7.8 Hz, 2H), 7.39 (d, *J* = 8.4 Hz, 2H), 7.33 (t, *J* = 7.8 Hz, 1H), 7.27–7.25 (m, 3H), 7.22 (t, *J* = 7.2 Hz, 2H), 5.96 (d, *J* = 0.6 Hz, 1H), 5.77 (d, *J* = 0.6 Hz, 1H), 5.45 (s, 1H), 5.08 (s, 1H), 2.23 (t, *J* = 7.8 Hz, 2H), 2.04 (t, *J* = 7.8 Hz, 2H), 1.94 (s, 3H); ^13^C{^1^H} NMR (150 MHz, CDCl_3_): *δ* 164.1, 155.4, 147.1, 144.7, 137.0, 135.7, 130.1, 130.0, 128.9, 128.2, 128.0, 127.9, 126.8, 124.4, 123.2, 121.9, 118.2, 112.5, 31.5, 31.3, 15.4. HRMS (ESI-TOF, *m*/*z*): calcd for C_28_H_25_N_3_O_3_NaS^+^, [M + Na]^+^, 506.1509, found 506.1509.

### 4-(1-(1-(4-(Methylthio)but-1-*en*-2-yl)-3-oxo-2,5-diphenyl-2,3-dihydro-1*H*-pyrazol-4-yl)vinyl) benzonitrile (3h)

The reaction was carried out on a 0.2 mmol scale following the General procedure. The product 3h was purified through column chromatography (PE/EtOAc: from 6 : 1 to 4 : 1) and obtained as a foam solid (84 mg, 91% yield). IR *ν*_max_ (neat)/cm^−1^: 3065, 2963, 2023, 1672, 1564, 1491, 1336, 1287, 1142, 1053, 965; ^1^H NMR (600 MHz, CDCl_3_): *δ* 7.58 (d, *J* = 7.2 Hz, 2H), 7.48 (t, *J* = 7.8, 2H), 7.39 (d, *J* = 7.2, 2H), 7.30–7.34 (m, 3H), 7.28 (d, *J* = 3.6 Hz, 1H), 7.27–7.25 (m, 2H), 7.22 (d, *J* = 7.2 Hz, 2H), 5.90 (s, 1H), 5.72 (s, 1H), 5.44 (s, 1H), 5.07 (s, 1H), 2.22 (t, *J* = 7.8 Hz, 2H), 2.03 (t, *J* = 7.8 Hz, 2H), 1.92 (s, 3H); ^13^C{^1^H} NMR (150 MHz, CDCl_3_): *δ* 164.1, 155.4, 145.1, 144.7, 137.2, 135.7, 131.7, 130.0, 128.9, 128.1, 127.7, 126.8, 124.3, 121.4, 118.7, 118.2, 112.5, 110.9, 31.4, 31.2, 15.4. HRMS (ESI-TOF, *m*/*z*): calcd for C_29_H_25_IN_3_ONaS^+^, [M + Na]^+^, 486.1611, found 486.1610.

### 1-(4-(Methylthio)but-1-*en*-2-yl)-2,5-diphenyl-4-(1-(*m*-tolyl)vinyl)-1,2-dihydro-3*H*-pyrazol-3-one (3i)

The reaction was carried out on a 0.2 mmol scale following the General procedure. The product 3i was purified through column chromatography (PE/EtOAc: from 6 : 1 to 4 : 1) and obtained as a foam solid (79 mg, 87% yield). IR *ν*_max_ (neat)/cm^−1^: 3106, 2932, 1688, 1492, 1356, 1149, 1028, 972, 855; ^1^H NMR (600 MHz, CDCl_3_): *δ* 7.64 (dd, *J* = 1.2, 8.4 Hz, 2H), 7.47 (t, *J* = 7.8 Hz, 2H), 7.34 (d, *J* = 7.2 Hz, 2H), 7.29 (t, *J* = 7.8 Hz, 1H), 7.26 (d, *J* = 7.2 Hz, 1H), 7.23 (t, *J* = 7.2 Hz, 2H), 7.13 (d, *J* = 7.8 Hz, 1H), 7.09 (d, *J* = 6.0 Hz, 1H), 7.06 (d, *J* = 7.2 Hz, 1H), 6.94 (d, *J* = 7.2 Hz, 1H), 5.67 (d, *J* = 1.2 Hz, 1H), 5.64 (d, *J* = 1.2 Hz, 1H), 5.44 (s, 1H), 5.05 (s, 1H), 2.26 (t, *J* = 7.8 Hz, 2H), 2.25 (s, 3H), 2.06 (t, *J* = 7.8 Hz, 2H), 1.95 (s, 3H); ^13^C{^1^H} NMR (150 MHz, CDCl_3_): *δ* 164.6, 155.5, 145.1, 140.1, 138.4, 137.2, 136.0, 130.0, 129.5, 129.3, 128.7, 128.0, 127.8, 127.8, 127.7, 126.3, 124.1, 118.5, 117.8, 114.3, 31.3, 31.1, 21.2, 15.3. HRMS (ESI-TOF, *m*/*z*): calcd for C_29_H_29_N_2_OS^+^, [M + H]^+^, 453.1995, found 453.1993.

### 4-(1-(3-Methoxyphenyl)vinyl)-1-(4-(methylthio)but-1-*en*-2-yl)-2,5-diphenyl-1,2-dihydro-3*H*-pyrazol-3-one (3j)

The reaction was carried out on a 0.2 mmol scale following the General procedure. The product 3j was purified through column chromatography (PE/EtOAc: from 6 : 1 to 4 : 1) and obtained as a foam solid (77 mg, 82% yield). IR *ν*_max_ (neat)/cm^−1^: 3088, 2898, 1667, 1584, 1492, 1336, 1177, 1062, 946; ^1^H NMR (600 MHz, CDCl_3_): *δ* 7.62 (d, *J* = 7.2 Hz, 2H), 7.46 (t, *J* = 7.8 Hz, 2H), 7.34 (d, *J* = 7.2 Hz, 2H), 7.30–7.26 (m, 2H), 7.23 (t, *J* = 7.2 Hz, 2H), 7.08 (t, *J* = 7.8 Hz, 1H), 6.92 (d, *J* = 7.8 Hz, 1H), 6.83 (s, 1H), 6.68 (dd, *J* = 1.8, 7.8 Hz, 1H), 5.68 (d, *J* = 1.2 Hz, 1H), 5.67 (s, 1H), 5.43 (s, 1H), 5.05 (s, 1H), 3.74 (s, 3H), 2.24 (t, *J* = 7.8 Hz, 2H), 2.05 (t, *J* = 7.8 Hz, 2H), 1.94 (s, 3H); ^13^C{^1^H} NMR (150 MHz, CDCl_3_): *δ* 164.5, 159.4, 155.5, 145.1, 141.7, 138.2, 135.9, 130.0, 129.6, 129.2, 128.9, 128.7, 127.8, 126.4, 124.1, 119.7, 118.9, 117.9, 114.2, 113.0, 112.9, 55.1, 31.3, 31.1, 15.3. HRMS (ESI-TOF, *m*/*z*): calcd for C_29_H_28_N_2_NaO_2_S^+^, [M + Na]^+^, 491.1764, found 491.1764.

### 4-(1-(3-Chlorophenyl)vinyl)-1-(4-(methylthio)but-1-*en*-2-yl)-2,5-diphenyl-1,2-dihydro-3*H*-pyrazol-3-one (3k)

The reaction was carried out on a 0.2 mmol scale following the General procedure. The product 3k was purified through column chromatography (PE/EtOAc: from 6 : 1 to 4 : 1) and obtained as a foam solid (80 mg, 85% yield). IR *ν*_max_ (neat)/cm^−1^: 3074, 2973, 1679, 1563, 1455, 1383, 1257, 1061, 977; ^1^H NMR (600 MHz, CDCl_3_): *δ* 7.61 (d, *J* = 7.2 Hz, 2H), 7.47 (t, *J* = 7.8 Hz, 2H), 7.31–7.30 (m, 3H), 7.29–7.21 (m, 4H), 7.15 (dt, *J* = 1.8, 4.8 Hz, 1H), 7.05 (d, *J* = 4.8 Hz, 2H), 5.81 (d, *J* = 1.2 Hz, 1H), 5.66 (d, *J* = 1.2 Hz, 1H), 5.42 (s, 1H), 5.05 (s, 1H), 2.23 (t, *J* = 7.8 Hz, 2H), 2.04 (t, *J* = 7.8 Hz, 2H), 1.94 (s, 3H); ^13^C{^1^H} NMR (150 MHz, CDCl_3_): *δ* 164.3, 155.4, 144.8, 142.2, 137.2, 135.8, 133.8, 130.0, 129.7, 129.1, 128.8, 127.9, 127.3, 127.1, 126.5, 125.2, 124.2, 119.8, 118.0, 113.1, 31.3, 31.2, 15.3. HRMS (ESI-TOF, *m*/*z*): calcd for C_28_H_25_ClN_2_NaOS^+^, [M + Na]^+^, 495.1268, found 495.1265.

### 1-(4-(Methylthio)but-1-*en*-2-yl)-2,5-diphenyl-4-(1-(*o*-tolyl)vinyl)-1,2-dihydro-3*H*-pyrazol-3-one (3l)

The reaction was carried out on a 0.2 mmol scale following the General procedure. The product 3l was purified through column chromatography (PE/EtOAc: from 6 : 1 to 4 : 1) and obtained as a foam solid (80 mg, 88% yield). IR *ν*_max_ (neat)/cm^−1^: 3106, 3023, 2984, 1688, 1492, 1356, 1149, 1028, 972, 855; ^1^H NMR (600 MHz, CDCl_3_): *δ* 7.59 (d, *J* = 7.2 Hz, 2H), 7.47 (t, *J* = 7.2 Hz, 2H), 7.30 (t, *J* = 7.2 Hz, 1H), 7.19–7.16 (m, 1H), 7.12–7.09 (m, 4H), 6.95 (dd, *J* = 1.2, 7.2 Hz, 1H), 6.90 (dt, *J* = 1.2, 7.2 Hz, 1H), 6.86 (t, *J* = 7.2 Hz, 1H), 6.81 (d, *J* = 7.8 Hz, 1H), 6.25 (d, *J* = 1.8 Hz, 1H), 5.37 (d, *J* = 1.8 Hz, 1H), 5.22 (s, 1H), 4.93 (s, 1H), 2.20 (t, *J* = 7.8 Hz, 2H), 2.10 (s, 3H), 2.00 (t, *J* = 7.8 Hz, 2H), 1.93 (s, 3H); ^13^C{^1^H} NMR (150 MHz, CDCl_3_): *δ* 164.2, 154.4, 144.5, 141.1, 138.3, 135.9, 135.7, 134.8, 129.7, 129.6, 129.5, 129.1, 128.7, 127.3, 126.7, 126.4, 124.9, 124.0, 120.1, 117.0, 112.9, 31.5, 31.0, 19.9, 15.2. HRMS (ESI-TOF, *m*/*z*): calcd for C_29_H_28_N_2_NaOS^+^, [M + Na]^+^, 475.1815, found 475.1813.

### 4-(1-(2-Methoxyphenyl)vinyl)-1-(4-(methylthio)but-1-*en*-2-yl)-2,5-diphenyl-1,2-dihydro-3*H*-pyrazol-3-one (3m)

The reaction was carried out on a 0.2 mmol scale following the General procedure. The product 3m was purified through column chromatography (PE/EtOAc: from 6 : 1 to 4 : 1) and obtained as a foam solid (79 mg, 84% yield). IR *ν*_max_ (neat)/cm^−1^: 3078, 2983, 1660, 1576, 1469, 1337, 1147, 1053, 983; ^1^H NMR (600 MHz, CDCl_3_): *δ* 7.59 (d, *J* = 7.2 Hz, 2H), 7.45 (t, *J* = 7.8 Hz, 2H), 7.27–7.25 (m, 3H), 7.21 (t, *J* = 7.2 Hz, 1H), 7.15 (t, *J* = 7.2 Hz, 2H), 7.13 (d, *J* = 7.2 Hz, 1H), 7.04 (dt, *J* = 1.8, 8.4 Hz, 1H), 6.75 (dt, *J* = 0.6, 7.2 Hz, 1H), 6.53 (d, *J* = 8.4 Hz, 1H), 6.03 (d, *J* = 1.8 Hz, 1H), 5.44 (d, *J* = 1.8 Hz, 1H), 5.30 (s, 1H), 4.96 (s, 1H), 3.68 (s, 3H), 2.22 (t, *J* = 7.8 Hz, 2H), 2.02 (t, *J* = 7.8 Hz, 2H), 1.93 (s, 3H); ^13^C{^1^H} NMR (150 MHz, CDCl_3_): *δ* 164.6, 156.4, 154.4, 145.1, 136.2, 130.6, 130.1, 129.4, 129.1, 128.6, 128.4, 127.3, 126.1, 123.9, 120.2, 117.1, 113.8, 110.5, 55.3, 31.2, 15.2. HRMS (ESI-TOF, *m*/*z*): calcd for C_29_H_28_N_2_NaO_2_S^+^, [M + Na]^+^, 491.1764, found 491.1766.

### 4-(1-(2-Chlorophenyl)vinyl)-1-(4-(methylthio)but-1-*en*-2-yl)-2,5-diphenyl-1,2-dihydro-3*H*-pyrazol-3-one (3n)

The reaction was carried out on a 0.2 mmol scale following the General procedure. The product 3n was purified through column chromatography (PE/EtOAc: from 6 : 1 to 4 : 1) and obtained as a foam solid (84 mg, 89% yield). IR *ν*_max_ (neat)/cm^−1^: 3056, 2933, 1679, 1586, 1453, 1332, 1175, 1026, 944; ^1^H NMR (600 MHz, CDCl_3_): *δ* 7.58 (d, *J* = 7.2 Hz, 2H), 7.45 (t, *J* = 7.2 Hz, 2H), 7.29 (t, *J* = 7.2 Hz, 1H), 7.23–7.18 (m, 3H), 7.17 (d, *J* = 6.6 Hz, 2H), 7.07 (dd, *J* = 1.8, 6.6 Hz, 1H), 7.03 (dd, *J* = 1.8, 6.6 Hz, 1H), 6.96–6.92 (m, 2H), 6.23 (d, *J* = 1.2 Hz, 1H), 5.43 (d, *J* = 1.2 Hz, 1H), 5.24 (s, 1H), 4.94 (s, 1H), 2.22 (t, *J* = 7.8 Hz, 2H), 2.02 (t, *J* = 7.8 Hz, 2H), 1.93 (s, 3H); ^13^C{^1^H} NMR (150 MHz, CDCl_3_): *δ* 164.2, 154.2, 144.5, 139.9, 136.5, 135.9, 132.3, 131.3, 129.9, 129.2, 129.1, 128.7, 128.0, 127.5, 126.4, 126.0, 124.1, 121.1, 117.1, 112.0, 31.7, 31.0, 15.2. HRMS (ESI-TOF, *m*/*z*): calcd for C_28_H_25_ClN_2_NaOS^+^, [M + Na]^+^, 495.1268, found 495.1270.

### 1-(4-(Methylthio)but-1-*en*-2-yl)-4-(1-(naphthalen-2-yl)vinyl)-2,5-diphenyl-1,2-dihydro-3*H*-pyrazol-3-one (3o)

The reaction was carried out on a 0.2 mmol scale following the General procedure. The product 3o was purified through column chromatography (PE/EtOAc: from 6 : 1 to 4 : 1) and obtained as a foam solid (65 mg, 66% yield). IR *ν*_max_ (neat)/cm^−1^: 3113, 2965, 1680, 1566, 1473, 1357, 1121, 1073, 961; ^1^H NMR (600 MHz, CDCl_3_): *δ* 7.87 (dd, *J* = 2.4, 7.2 Hz, 1H), 7.62 (d, *J* = 4.8 Hz, 1H), 7.61 (t, *J* = 7.2 Hz, 2H), 7.49 (t, *J* = 7.2 Hz, 2H), 7.44 (d, *J* = 7.8 Hz, 1H), 7.38–7.36 (m, 2H), 7.32 (t, *J* = 7.2 Hz, 1H), 7.13–7.08 (m, 2H), 6.95 (t, *J* = 7.2 Hz, 1H), 6.79 (t, *J* = 7.2 Hz, 2H), 6.68 (d, *J* = 7.8 Hz, 2H), 6.61 (d, *J* = 2.4 Hz, 1H), 5.58 (d, *J* = 2.4 Hz, 1H), 5.06 (s, 1H), 4.83 (s, 1H), 2.16 (t, *J* = 7.8 Hz, 2H), 1.98 (t, *J* = 7.8 Hz, 2H), 1.91 (s, 3H); ^13^C{^1^H} NMR (150 MHz, CDCl_3_): *δ* 164.3, 154.8, 144.3, 139.5, 137.4, 136.0, 133.4, 131.4, 129.5, 128.8, 128.7, 127.9, 127.3, 127.0, 126.8, 126.5, 126.0, 125.5, 125.2, 124.8, 124.1, 120.8, 116.7, 112.9, 32.0, 31.1, 15.3. HRMS (ESI-TOF, *m*/*z*): calcd for C_32_H_28_ClN_2_NaOS^+^, [M + Na]^+^, 511.1815, found 511.1818.

### 1-(4-(Methylthio)but-1-*en*-2-yl)-4-(1-(naphthalen-2-yl)vinyl)-2,5-diphenyl-1,2-dihydro-3*H*-pyrazol-3-one (3p)

The reaction was carried out on a 0.2 mmol scale following the General procedure. The product 3p was purified through column chromatography (PE/EtOAc: from 6 : 1 to 4 : 1) and obtained as a foam solid (74 mg, 76% yield). IR *ν*_max_ (neat)/cm^−1^: 3039, 2976, 1677, 1562, 1473, 1390, 1124, 973, 837; ^1^H NMR (600 MHz, CDCl_3_): *δ* 7.76 (d, *J* = 4.8 Hz, 2H), 7.74 (d, *J* = 7.2 Hz, 1H), 7.68–7.65 (m, 3H), 7.51–7.48 (m, 3H), 7.44–7.40 (m, 2H), 7.38–7.37 (m, 2H), 7.31 (t, *J* = 7.2 Hz, 1H), 7.16–7.14 (m, 3H), 5.84 (s, 1H), 5.74 (s, 1H), 5.47 (s, 1H), 5.07 (s, 1H), 2.29 (t, *J* = 7.8 Hz, 2H), 2.11 (t, *J* = 7.8 Hz, 2H), 1.97 (s, 3H); ^13^C{^1^H} NMR (150 MHz, CDCl_3_): *δ* 164.6, 155.7, 145.1, 138.3, 137.7, 135.9, 133.2, 132.8, 129.9, 129.5, 129.1, 128.7, 128.1, 127.8, 127.5, 127.3, 126.4, 126.0, 125.7, 125.6, 125.1, 124.1, 119.2, 117.9, 114.3, 31.3, 31.2, 15.3. HRMS (ESI-TOF, *m*/*z*): calcd for C_32_H_28_ClN_2_NaOS^+^, [M + Na]^+^, 511.1815, found 511.1816.

### 4-(1-(6-Methoxynaphthalen-2-yl)vinyl)-1-(4-(methylthio)but-1-*en*-2-yl)-2,5-diphenyl-1,2-dihydro-3*H*-pyrazol-3-one (3q)

The reaction was carried out on a 0.2 mmol scale following the General procedure. The product 3q was purified through column chromatography (PE/EtOAc: from 6 : 1 to 4 : 1) and obtained as a foam solid (86 mg, 83% yield). IR *ν*_max_ (neat)/cm^−1^: 3027, 2917, 1673, 1510, 1445, 1307, 1011, 904, 816; ^1^H NMR (600 MHz, CDCl_3_): *δ* 7.69–7.65 (m, 4H), 7.56 (d, *J* = 9.0 Hz, 1H), 7.50–7.47 (m, 3H), 7.39–7.38 (m, 2H), 7.30 (t, *J* = 7.2 Hz, 1H), 7.17–7.16 (m, 4H), 7.11 (dd, *J* = 2.4, 9.0 Hz, 1H), 7.05 (d, *J* = 2.4 Hz, 1H), 5.81 (d, *J* = 0.6 Hz, 1H), 5.67 (s, 1H), 5.47 (s, 1H), 5.07 (s, 1H), 3.89 (s, 3H), 2.28 (t, *J* = 7.8 Hz, 2H), 2.10 (t, *J* = 7.8 Hz, 2H), 1.96 (s, 3H); ^13^C{^1^H} NMR (150 MHz, CDCl_3_): *δ* 164.5, 157.6, 155.6, 145.0, 138.2, 135.9, 135.4, 133.9, 129.8, 129.5, 129.1, 128.7, 127.7, 126.4, 126.3, 125.7, 125.4, 124.0, 118.4, 118.4, 117.9, 114.5, 105.8, 55.1, 31.2, 31.1, 15.3. HRMS (ESI-TOF, *m/z*): calcd for C_33_H_30_N_2_NaO_2_S^+^, [M + Na]^+^, 541.1920, found 541.1922.

### 1-(4-(Methylthio)but-1-*en*-2-yl)-2,5-diphenyl-4-(thiophen-2-yl)-1,2-dihydro-3*H*-pyrazol-3-one (3r)

The reaction was carried out on a 0.2 mmol scale following the General procedure. The product 3r was purified through column chromatography (PE/EtOAc: from 6 : 1 to 4 : 1) and obtained as a foam solid (51 mg, 57% yield). IR *ν*_max_ (neat)/cm^−1^: 3032, 2987, 1681, 1527, 1397, 1262, 1098, 1002, 935, 882; ^1^H NMR (600 MHz, CDCl_3_): *δ* 7.63 (d, *J* = 7.8 Hz, 2H), 7.48–7.45 (m, 4H), 7.37–7.29 (m, 4H), 7.09 (d, *J* = 5.4 Hz, 1H), 6.95 (d, *J* = 3.6 Hz, 1H), 6.84 (dd, *J* = 3.6, 5.4 Hz, 1H), 5.73 (s, 1H), 5.48 (s, 1H), 5.34 (s, 1H), 5.08 (s, 1H), 2.26 (t, *J* = 7.8 Hz, 2H), 2.06 (t, *J* = 7.8 Hz, 2H), 1.94 (s, 3H); ^13^C{^1^H} NMR (150 MHz, CDCl_3_): *δ* 164.1, 155.7, 145.0, 143.9, 135.8, 131.8, 129.8, 129.6, 129.0, 128.7, 128.0, 127.0, 126.4, 125.1, 124.9, 124.4, 124.3, 124.2, 118.1, 117.5, 114.2, 31.2, 31.0, 15.3. HRMS (ESI-TOF, *m*/*z*): calcd for C_26_H_25_N_2_OS^+^, [M + H]^+^, 445.1403, found 445.1405.

### 4-(1-(1-Methyl-1H-pyrrol-2-yl)vinyl)-1-(4-(methylthio)but-1-*en*-2-yl)-2,5-diphenyl-1,2-dihydro-3*H*-pyrazol-3-one (3s)

The reaction was carried out on a 0.2 mmol scale following the General procedure. The product 3s was purified through column chromatography (PE/EtOAc: from 6 : 1 to 4 : 1) and obtained as a foam solid (62 mg, 70% yield). IR *ν*_max_ (neat)/cm^−1^: 3072, 2976, 1672, 1476, 1331, 1229, 1152, 1034, 913, 826; ^1^H NMR (600 MHz, CDCl_3_): *δ* 7.59 (d, *J* = 7.8 Hz, 2H), 7.46 (t, *J* = 7.8 Hz, 2H), 7.31 (t, *J* = 7.8 Hz, 4H), 7.28–7.25 (m, 2H), 6.29 (t, *J* = 2.4 Hz, 1H), 5.92 (dd, *J* = 1.8, 3.0 Hz, 1H), 5.85 (d, *J* = 1.8 Hz, 1H), 5.84 (s, 1H), 5.43 (d, *J* = 1.8 Hz, 1H), 5.38 (s, 1H), 5.02 (s, 1H), 3.42 (s, 3H), 2.22 (t, *J* = 7.8 Hz, 2H), 2.01 (t, *J* = 7.8 Hz, 2H), 1.93 (s, 3H); ^13^C{^1^H} NMR (150 MHz, CDCl_3_): *δ* 164.3, 155.0, 144.9, 135.8, 133.2, 129.7, 129.5, 128.7, 127.6, 126.4, 124.1, 122.8, 119.4, 117.7, 114.0, 109.8, 107.2, 34.5, 31.2, 15.3. HRMS (ESI-TOF, *m*/*z*): calcd for C_27_H_27_N_3_NaOS^+^, [M + Na]^+^, 464.1767, found 464.1772.

### 1-(4-(Methylthio)but-1-*en*-2-yl)-2,5-diphenyl-4-(prop-1-*en*-2-yl)-1,2-dihydro-3*H*-pyrazol-3-one (3t)

The reaction was carried out on a 0.2 mmol scale following the General procedure. The product 3t was purified through column chromatography (PE/EtOAc: from 6 : 1 to 4 : 1) and obtained as a foam solid (54 mg, 72% yield). IR *ν*_max_ (neat)/cm^−1^: 3051, 2982, 1672, 1568, 2448, 1379, 1253, 1062, 937, 784; ^1^H NMR (600 MHz, CDCl_3_): *δ* 7.55 (d, *J* = 7.8 Hz, 2H), 7.47–7.43 (m, 7H), 7.28 (t, *J* = 7.2 Hz, 1H), 5.46 (s, 1H), 5.31 (s, 1H), 5.15 (s, 1H), 4.98 (s, 1H), 2.21 (t, *J* = 7.8 Hz, 2H), 1.99 (t, *J* = 7.8 Hz, 2H), 1.93 (s, 3H), 1.77 (s, 3H); ^13^C{^1^H} NMR (150 MHz, CDCl_3_): *δ* 164.3, 153.7, 144.9, 135.9, 133.8, 130.2, 129.8, 128.7, 128.2, 126.4, 124.1, 117.4, 117.2, 114.7, 31.3, 31.2, 22.6, 15.3. HRMS (ESI-TOF, *m*/*z*): calcd for C_23_H_25_N_2_OS^+^, [M + H]^+^, 377.5255, found 377.5256.

### 1-(4-(Methylthio)but-1-*en*-2-yl)-2,5-diphenyl-4-(thiophen-2-yl)-1,2-dihydro-3*H*-pyrazol-3-one (3aa)

The reaction was carried out on a 0.2 mmol scale following the General procedure. The product 3aa was purified through column chromatography (PE/EtOAc: from 6 : 1 to 4 : 1) and obtained as a foam solid (57 mg, 63% yield). IR *ν*_max_ (neat)/cm^−1^: 3022, 2918, 1672, 1580, 1397, 1301, 1189, 1011, 934, 781; ^1^H NMR (600 MHz, CDCl_3_): *δ* 7.49 (d, *J* = 8.4 Hz, 2H), 7.34–7.30 (m, 4H), 7.28–7.24 (m, 3H), 7.21 (t, *J* = 7.2 Hz, 2H), 7.16 (t, *J* = 7.2 Hz, 2H), 7.11 (t, *J* = 7.2 Hz, 1H), 5.68 (d, *J* = 1.2 Hz, 1H), 5.67 (d, *J* = 1.2 Hz, 1H), 5.41 (s, 1H), 5.04 (s, 1H), 2.40 (s, 3H), 2.25 (t, *J* = 7.8 Hz, 2H), 2.05 (t, *J* = 7.8 Hz, 2H), 1.95 (s, 3H). ^13^C{^1^H} NMR (150 MHz, CDCl_3_): *δ* 164.5, 155.1, 145.1, 140.3, 138.4, 136.3, 133.4, 130.0, 129.5, 129.3, 127.8, 127.8, 127.1, 126.9, 124.3, 118.7, 117.8, 114.1, 31.2, 20.9, 15.3. HRMS (ESI-TOF, *m*/*z*): calcd for C_29_H_28_N_2_NaOS^+^, [M + Na]^+^, 475.1815, found 475.1817.

### 2-(4-Methoxyphenyl)-1-(4-(methylthio)but-1-*en*-2-yl)-5-phenyl-4-(1-phenylvinyl)-1,2-dihydro-3H-pyrazol-3-one (3ab)

The reaction was carried out on a 0.2 mmol scale following the General procedure. The product 3ab was purified through column chromatography (PE/EtOAc: from 6 : 1 to 4 : 1) and obtained as a foam solid (56 mg, 60% yield). IR *ν*_max_ (neat)/cm^−1^: 3030, 2944, 1677, 1589, 1491, 1270, 1086, 933, 812; ^1^H NMR (600 MHz, CDCl_3_): *δ* 7.50 (d, *J* = 9.0 Hz, 2H), 7.32–7.30 (m, 4H), 7.25 (t, *J* = 7.2 Hz, 1H), 7.20 (t, *J* = 7.2 Hz, 2H), 7.15 (t, *J* = 7.2 Hz, 2H), 7.11 (t, *J* = 7.2 Hz, 1H), 6.99 (d, *J* = 9.0 Hz, 2H), 5.67 (s, 1H), 5.66 (s, 1H), 5.39 (s, 1H), 5.05 (s, 1H), 3.84 (s, 3H), 2.25 (t, *J* = 7.8 Hz, 2H), 2.04 (t, *J* = 7.8 Hz, 2H), 1.95 (s, 3H); ^13^C{^1^H} NMR (150 MHz, CDCl_3_): *δ* 164.7, 158.5, 154.7, 145.1, 140.4, 138.5, 130.0, 129.5, 129.4, 128.9, 127.9, 127.8, 127.2, 127.0, 126.3, 118.7, 118.0, 114.3, 114.0, 55.5, 31.3, 15.3. HRMS (ESI-TOF, *m*/*z*): calcd for C_29_H_29_N_2_OS^+^, [M + H]^+^, 469.1944, found 469.1946.

### 2-(4-Fluorophenyl)-1-(4-(methylthio)but-1-*en*-2-yl)-5-phenyl-4-(1-phenylvinyl)-1,2-dihydro-3*H*-pyrazol-3-one (3ac)

The reaction was carried out on a 0.2 mmol scale following the General procedure. The product 3ac was purified through column chromatography (PE/EtOAc: from 6 : 1 to 4 : 1) and obtained as a foam solid (67 mg, 73% yield). IR *ν*_max_ (neat)/cm^−1^: 3037, 2975, 1658, 1589, 1452, 1358, 1284, 1031, 982; ^1^H NMR (600 MHz, CDCl_3_): *δ* 7.60–7.57 (m, 2H), 7.32 (dd, *J* = 7.2, 8.4 Hz, 2H), 7.29 (t, *J* = 6.6 Hz, 2H), 7.26 (d, *J* = 7.2 Hz, 1H), 7.22 (t, *J* = 7.2 Hz, 2H), 7.18–7.15 (m, 4H), 7.13 (t, *J* = 7.2 Hz, 1H), 5.68 (d, *J* = 1.2 Hz, 1H), 5.64 (d, *J* = 1.2 Hz, 1H), 5.42 (s, 1H), 5.07 (s, 1H), 2.26 (t, *J* = 7.8 Hz, 2H), 2.04 (t, *J* = 7.8 Hz, 2H), 1.95 (s, 3H); ^13^C{^1^H} NMR (150 MHz, CDCl_3_): *δ* 164.8, 161.9, 160.3, 155.7, 145.2, 140.2, 138.4, 132.1, 130.0, 129.7, 129.2, 128.0, 127.3, 126.9, 126.1, 126.1, 119.0, 118.0, 115.8, 115.6, 114.3, 31.4, 31.1, 15.4. ^19^F NMR (600 MHz, CDCl_3_): *δ* −114.98. HRMS (ESI-TOF, *m*/*z*): calcd for C_28_H_26_FN_2_OS^+^, [M + H]^+^, 457.1744, found 457.1746.

### 2-(4-Bromophenyl)-5-(4-methoxyphenyl)-1-(4-(methylthio)but-1-*en*-2-yl)-4-(1-phenylvinyl)-1,2-dihydro-3*H*-pyrazol-3-one (3ad)

The reaction was carried out on a 0.2 mmol scale following the General procedure. The product 3ad was purified through column chromatography (PE/EtOAc: from 6 : 1 to 4 : 1) and obtained as a foam solid (68 mg, 66% yield). IR *ν*_max_ (neat)/cm^−1^: 3033, 2989, 1668, 1565, 1487, 1312, 1220, 1101, 979, 856; ^1^H NMR (600 MHz, CDCl_3_): *δ* 7.59 (d, *J* = 7.8 Hz, 2H), 7.52 (d, *J* = 8.4 Hz, 2H), 7.33 (d, *J* = 7.2 Hz, 2H), 7.30 (d, *J* = 7.2 Hz, 2H), 7.26 (d, *J* = 7.2 Hz, 1H), 7.23 (t, *J* = 7.2 Hz, 2H), 7.17 (t, *J* = 7.2 Hz, 2H), 7.13 (t, *J* = 7.2 Hz, 1H), 5.68 (d, *J* = 0.6 Hz, 1H), 5.62 (d, *J* = 0.6 Hz, 1H), 5.44 (s, 1H), 5.08 (s, 1H), 2.26 (t, *J* = 7.8 Hz, 2H), 2.04 (t, *J* = 7.8 Hz, 2H), 1.95 (s, 3H). ^13^C{^1^H} NMR (150 MHz, CDCl_3_): *δ* 164.5, 156.1, 145.2, 140.1, 138.2, 135.1, 131.9, 130.0, 129.8, 129.7, 129.0, 127.9, 127.3, 126.9, 125.3, 119.8, 119.0, 118.0, 114.5, 31.4, 30.9, 15.4. HRMS (ESI-TOF, *m*/*z*): calcd for C_28_H_25_BrN_2_NaOS^+^, [M + Na]^+^, 539.0763, found 539.0763.

### 2-(2-Ethylphenyl)-1-(4-(methylthio)but-1-*en*-2-yl)-5-phenyl-4-(1-phenylvinyl)-1,2-dihydro-3*H*-pyrazol-3-one (3ae)

The reaction was carried out on a 0.2 mmol scale following the General procedure. The product 3ae was purified through column chromatography (PE/EtOAc: from 6 : 1 to 4 : 1) and obtained as a foam solid (47 mg, 50% yield). IR *ν*_max_ (neat)/cm^−1^: 3038, 2933, 1681, 157, 1392, 1267, 1083, 962, 828; ^1^H NMR (600 MHz, CDCl_3_): *δ* 7.42 (d, *J* = 6.6 Hz, 1H), 7.39 (dt, *J* = 1.2, 7.2 Hz, 1H), 7.35 (d, *J* = 7.2 Hz, 1H), 7.32–7.29 (m, 5H), 7.23 (d, *J* = 7.2 Hz, 1H), 7.19 (t, *J* = 7.2 Hz, 2H), 7.13 (t, *J* = 7.2 Hz, 2H), 7.09 (t, *J* = 7.2 Hz, 1H), 5.74 (d, *J* = 1.2 Hz, 1H), 5.72 (d, *J* = 1.2 Hz, 1H), 5.20 (s, 1H), 4.99 (s, 1H), 2.91–2.84 (m, 1H), 2.73–2.67 (m, 1H), 2.27–2.24 (m, 2H), 2.11–2.07 (m, 2H), 1.94 (s, 3H), 1.34 (t, *J* = 7.2 Hz, 3H). ^13^C{^1^H} NMR (150 MHz, CDCl_3_): *δ* 165.6, 154.1, 144.8, 142.8, 140.5, 138.5, 134.4, 129.8, 129.5, 129.4, 129.0, 127.9, 127.8, 127.1, 127.0, 126.2, 118.8, 118.0, 113.0, 32.1, 31.1, 23.9, 15.4, 14.0. HRMS (ESI-TOF, *m*/*z*): calcd for C_30_H_30_N_2_NaOS^+^, [M + Na]^+^, 489.1971, found 489.1972.

### 2-(2-Fluorophenyl)-1-(4-(methylthio)but-1-*en*-2-yl)-5-phenyl-4-(1-phenylvinyl)-1,2-dihydro-3*H*-pyrazol-3-one (3af)

The reaction was carried out on a 0.2 mmol scale following the General procedure. The product 3af was purified through column chromatography (PE/EtOAc: from 6 : 1 to 4 : 1) and obtained as a foam solid (50 mg, 55% yield). IR *ν*_max_ (neat)/cm^−1^: 3062, 2983, 1668, 1549, 1433, 1327, 1252, 1098, 972, 819; ^1^H NMR (600 MHz, CDCl_3_): *δ* 7.51 (t, *J* = 6.0 Hz, 1H), 7.41–7.37 (m, 1H), 7.33–7.25 (m, 4H), 7.23 (d, *J* = 7.8 Hz, 3H), 7.20 (t, *J* = 7.2 Hz, 2H), 7.13 (t, *J* = 7.2 Hz, 2H), 7.09 (t, *J* = 7.2 Hz, 1H), 5.74 (d, *J* = 1.2 Hz, 1H), 5.70 (d, *J* = 1.2 Hz, 1H), 5.39 (s, 1H), 5.05 (s, 1H), 2.27 (t, *J* = 7.8 Hz, 2H), 2.08 (t, *J* = 7.8 Hz, 2H), 1.94 (s, 3H); ^13^C{^1^H} NMR (150 MHz, CDCl_3_): *δ* 165.2, 158.8, 157.2, 155.2, 144.8, 140.3, 138.3, 130.1, 130.0, 129.8, 129.6, 129.1, 127.9, 127.8, 127.1, 127.0, 124.3, 118.8, 118.4, 116.8, 116.7, 113.1, 31.5, 31.2, 15.3. ^19^F NMR (600 MHz, CDCl_3_): *δ* −118.90. HRMS (ESI-TOF, *m*/*z*): calcd for C_28_H_25_FN_2_NaOS^+^, [M + Na]^+^, 479.1564, found 479.1566.

### 1-(4-(Methylthio)but-1-*en*-2-yl)-2-(perfluorophenyl)-5-phenyl-4-(1-phenylvinyl)-1,2-dihydro-3*H*-pyrazol-3-one (3ag)

The reaction was carried out on a 0.2 mmol scale following the General procedure. The product 3ag was purified through column chromatography (PE/EtOAc: from 6 : 1 to 4 : 1) and obtained as a foam solid (74 mg, 70% yield). IR *ν*_max_ (neat)/cm^−1^: 3066, 2952, 1561, 1445, 1270, 1184, 1077, 977, 835; ^1^H NMR (600 MHz, CDCl_3_): *δ* 7.32 (d, *J* = 6.6 Hz, 2H), 7.28–7.26 (m, 3H), 7.20 (t, *J* = 7.2 Hz, 2H), 7.14 (dd, *J* = 6.0, 7.8 Hz, 2H), 7.10 (t, *J* = 7.2 Hz, 1H), 5.75 (d, *J* = 1.2 Hz, 1H), 5.71 (d, *J* = 1.2 Hz, 1H), 5.40 (s, 1H), 5.14 (s, 1H), 2.33 (t, *J* = 7.8 Hz, 2H), 2.11 (t, *J* = 7.8 Hz, 2H), 1.96 (s, 3H); ^13^C{^1^H} NMR (150 MHz, CDCl_3_): *δ* 165.9, 157.2, 145.9, 145.3, 144.2, 141.3, 139.9, 138.9, 137.8, 137.2, 130.2, 130.0, 129.7, 129.6, 128.6, 128.1, 128.0, 127.4, 126.9, 119.3, 118.5, 113.1, 111.8, 31.1, 31.1, 15.3. ^19^F NMR (600 MHz, CDCl_3_): *δ* −142.99, −143.02, −151.13, −151.17, −151.21, −161.11, −161.14, −161.14, −161.18. HRMS (ESI-TOF, *m*/*z*): calcd for C_28_H_21_F_5_N_2_NaOS^+^, [M + Na]^+^, 551.1187, found 551.1188.

### 5-(4-Fluorophenyl)-1-(4-(methylthio)but-1-*en*-2-yl)-2-phenyl-4-(1-phenylvinyl)-1,2-dihydro-3*H*-pyrazol-3-one (3ah)

The reaction was carried out on a 0.2 mmol scale following the General procedure. The product 3ah was purified through column chromatography (PE/EtOAc: from 6 : 1 to 4 : 1) and obtained as a foam solid (47 mg, 51% yield). IR *ν*_max_ (neat)/cm^−1^: 3036, 2942, 1670, 1589, 1433, 1362, 1098, 937, 828; ^1^H NMR (600 MHz, CDCl_3_): *δ* 7.61 (d, *J* = 7.8 Hz, 2H), 7.47 (t, *J* = 7.8 Hz, 2H), 7.32–7.30 (m, 3H), 7.27 (dd, *J* = 1.2, 7.8 Hz, 2H), 7.17–7.13 (m, 3H), 6.90 (t, *J* = 9.0 Hz, 2H), 5.72 (d, *J* = 1.2 Hz, 1H), 5.70 (d, *J* = 1.2 Hz, 1H), 5.44 (s, 1H), 5.08 (s, 1H), 2.24 (t, *J* = 7.8 Hz, 2H), 2.03 (t, *J* = 7.8 Hz, 2H), 1.95 (s, 3H); ^13^C{^1^H} NMR (150 MHz, CDCl_3_): *δ* 164.5, 164.2, 162.5, 154.3, 145.2, 140.2, 138.2, 135.9, 132.0, 132.0, 128.8, 128.0, 127.4, 127.0, 126.5, 125.3, 124.2, 119.1, 118.2, 115.2, 115.1, 114.5, 31.3, 31.0, 15.4. ^19^F NMR (600 MHz, CDCl_3_): *δ* −110.07. HRMS (ESI-TOF, *m*/*z*): calcd for C_28_H_26_FN_2_OS^+^, [M + H]^+^, 457.1744, found 457.1744.

### 5-(4-Methoxyphenyl)-1-(4-(methylthio)but-1-*en*-2-yl)-4-(1-phenylvinyl)-2-(*p*-tolyl)-1,2-dihydro-3*H*-pyrazol-3-one (3ai)

The reaction was carried out on a 0.2 mmol scale following the General procedure. The product 3ai was purified through column chromatography (PE/EtOAc: from 6 : 1 to 4 : 1) and obtained as a foam solid (69 mg, 71% yield). IR *ν*_max_ (neat)/cm^−1^: 3052, 2989, 1671, 1489, 1345, 1287, 1176, 1044, 929, 817; ^1^H NMR (600 MHz, CDCl_3_): *δ* 7.48 (d, *J* = 8.4 Hz, 2H), 7.33 (d, *J* = 7.2 Hz, 2H), 7.27 (t, *J* = 8.4 Hz, 4H), 7.17 (t, *J* = 7.2 Hz, 2H), 7.13 (t, *J* = 7.2 Hz, 1H), 6.73 (d, *J* = 8.4 Hz, 2H), 5.70 (d, *J* = 1.2 Hz, 1H), 5.64 (s, 1H), 5.43 (s, 1H), 5.06 (s, 1H), 3.75 (s, 3H), 2.39 (s, 3H), 2.25 (t, *J* = 7.8 Hz, 2H), 2.04 (t, *J* = 7.8 Hz, 2H), 1.95 (s, 3H); ^13^C{^1^H} NMR (150 MHz, CDCl_3_): *δ* 164.8, 160.6, 155.1, 145.4, 140.2, 138.5, 136.2, 133.5, 131.4, 129.3, 127.8, 127.1, 126.8, 124.3, 121.4, 118.5, 117.9, 113.4, 55.1, 31.3, 31.0, 20.9, 15.3. HRMS (ESI-TOF, *m*/*z*): calcd for C_30_H_30_N_2_NaO_2_S^+^, [M + Na]^+^, 505.1920, found 505.1920.

### 5-(4-Bromophenyl)-1-(4-(methylthio)but-1-*en*-2-yl)-4-(1-phenylvinyl)-2-(*p*-tolyl)-1,2-dihydro-3*H*-pyrazol-3-one (3aj)

The reaction was carried out on a 0.2 mmol scale following the General procedure. The product 3aj was purified through column chromatography (PE/EtOAc: from 6 : 1 to 4 : 1) and obtained as a foam solid (53 mg, 50% yield). IR *ν*_max_ (neat)/cm^−1^: 3039, 2962, 1670, 1527, 1363, 1299, 1108, 1036, 898; ^1^H NMR (600 MHz, CDCl_3_): *δ* 7.46 (d, *J* = 8.4 Hz, 2H), 7.35 (d, *J* = 8.4 Hz, 2H), 7.28–7.26 (m, 4H), 7.19–7.16 (m, 5H), 5.69 (s, 1H), 5.68 (s, 1H), 5.40 (s, 1H), 5.07 (s, 1H), 2.40 (s, 3H), 2.25 (t, *J* = 7.8 Hz, 2H), 2.02 (t, *J* = 7.8 Hz, 2H), 1.96 (s, 3H). ^13^C{^1^H} NMR (150 MHz, CDCl_3_): *δ* 164.4, 153.7, 145.2, 140.3, 138.3, 136.6, 133.3, 132.0, 131.5, 131.2, 131.1, 129.5, 128.3, 128.1, 127.4, 127.0, 124.4, 124.2, 124.1, 119.1, 118.8, 118.1, 114.8, 31.4, 31.2, 21.0, 15.4. HRMS (ESI-TOF, *m*/*z*): calcd for C_29_H_28_BrN_2_OS^+^, [M + H]^+^, 531.1100, found 531.1103.

### 5-Methyl-1-(4-(methylthio)but-1-*en*-2-yl)-2-phenyl-4-(1-phenylvinyl)-1,2-dihydro-3*H*-pyrazol-3-one (3ak)

The reaction was carried out on a 0.2 mmol scale following the General procedure. The product 3ak was purified through column chromatography (PE/EtOAc: from 6 : 1 to 4 : 1) and obtained as a foam solid (62 mg, 83% yield). IR *ν*_max_ (neat)/cm^−1^: 3072, 2916, 1667, 1539, 1420, 1331, 1298, 1032, 919, 827; ^1^H NMR (600 MHz, CDCl_3_): *δ* 7.49 (d, *J* = 7.2 Hz, 2H), 7.49–7.40 (m, 2H), 7.34 (t, *J* = 7.2 Hz, 2H), 7.29 (t, *J* = 7.2 Hz, 1H), 7.25 (t, *J* = 7.2 Hz, 1H), 5.73 (d, *J* = 1.2 Hz, 1H), 5.66 (d, *J* = 1.2 Hz, 1H), 5.38 (s, 1H), 5.27 (s, 1H), 2.36 (t, *J* = 7.2 Hz, 2H), 2.23 (t, *J* = 7.2 Hz, 2H), 2.03 (s, 3H), 1.98 (s, 3H); ^13^C{^1^H} NMR (150 MHz, CDCl_3_): *δ* 164.8, 152.1, 143.7, 141.0, 138.6, 136.4, 128.7, 128.3, 127.5, 126.9, 126.0, 123.2, 117.9, 115.4, 110.8, 32.6, 30.9, 15.4, 13.0. HRMS (ESI-TOF, *m*/*z*): calcd for C_23_H_25_N_2_OS^+^, [M + H]^+^, 377.1682, found 377.1686.

### 5-Methyl-1-(4-(methylthio)but-1-*en*-2-yl)-2-phenyl-4-(prop-1-*en*-2-yl)-1,2-dihydro-3*H*-pyrazol-3-one (3al)

The reaction was carried out on a 0.2 mmol scale following the General procedure. The product 3al was purified through column chromatography (PE/EtOAc: from 6 : 1 to 4 : 1) and obtained as a foam solid (51 mg, 81% yield). IR *ν*_max_ (neat)/cm^−1^: 3046, 2982, 1670, 1533, 1478, 1324, 1278, 1109, 1082, 952; ^1^H NMR (600 MHz, CDCl_3_): *δ* 7.43–7.39 (m, 4H), 7.25–7.22 (m, 1H), 5.38 (s, 1H), 5.27 (s, 1H), 5.20 (d, *J* = 1.2 Hz, 1H), 5.09 (d, *J* = 1.2 Hz, 1H), 2.32 (s, 3H), 2.31 (t, *J* = 7.8 Hz, 2H), 2.18 (t, *J* = 7.8 Hz, 2H), 2.14 (s, 3H), 2.00 (s, 3H); ^13^C{^1^H} NMR (150 MHz, CDCl_3_): *δ* 164.7, 150.4, 144.0, 136.4, 135.9, 128.7, 126.0, 123.3, 115.8, 115.3, 112.5, 32.5, 31.0, 22.3, 15.4, 12.8. HRMS (ESI-TOF, *m*/*z*): calcd for C_18_H_22_N_2_NaOS^+^, [M + Na]^+^, 337.1345, found 337.1345.

### Reaction of unsaturated pyrazolones 1a with propargyl sulfide ylide 2b

To a flame-dried sealable 3-dram vial equipped with a stir bar was added unsaturated pyrazolinones 1a (68 mg, 0.2 mmol, 1.0 equiv.), NaOAc·3H_2_O (14 mg, 0.1 mmol, 0.5 equiv.) and 2b (50 mg, 0.24 mmol, 1.2 equiv.) under air. Subsequently treated CH_3_CN (2 mL, *c* = 0.1 M) was added to vial *via* syringe. The reaction mixture was stirred for 15 min at 20 °C until unsaturated pyrazolones 1a was fully consumed (monitored by TLC). Then the reaction was stirred at 0 °C for 20 h. The organic solvent was removed under reduced pressure and purified through column chromatography (eluent: petroleum ether and EtOAc) to afford the desired product 4 (23% yield, 22 mg) and 5 (47% yield, 41 mg).

### 1-(4-(Ethylthio)pent-1-*en*-2-yl)-2,5-diphenyl-4-(1-phenylvinyl)-1,2-dihydro-3*H*-pyrazol-3-one (4)

IR *ν*_max_ (neat)/cm^−1^: 3082, 2977, 2864, 1705, 1594, 1466, 1272, 1042, 967, 832; ^1^H NMR (600 MHz, CDCl_3_): *δ* 7.63 (dd, *J* = 1.2, 8.4 Hz, 2H), 7.47 (t, *J* = 7.8 Hz, 2H), 7.36 (d, *J* = 8.4 Hz, 2H), 7.32–7.25 (m, 4H), 7.22 (t, *J* = 7.2 Hz, 2H), 7.17–7.11 (m, 3H), 5.69 (d, *J* = 1.2 Hz, 1H), 5.63 (d, *J* = 1.2 Hz, 1H), 5.52 (s, 1H), 5.09 (s, 1H), 2.64–2.59 (m, 1H), 2.41 (q, *J* = 7.8 Hz, 2H), 2.19 (dd, *J* = 4.8, 15.6 Hz, 1H), 1.77 (dd, *J* = 9.6, 15.6 Hz, 1H), 1.18 (t, *J* = 7.2 Hz, 3H), 0.78 (d, *J* = 7.2 Hz, 1H); ^13^C{^1^H} NMR (150 MHz, CDCl_3_): *δ* 164.7, 155.6, 144.1, 140.3, 138.4, 136.2, 130.3, 129.7, 129.3, 128.7, 128.0, 127.9, 127.3, 127.0, 126.4, 124.2, 119.2, 118.9, 114.3, 39.2, 36.4, 24.6, 20.5, 14.7. HRMS (ESI-TOF, *m*/*z*): calcd for C_30_H_30_N_2_NaOS^+^, [M + Na]^+^, 489.1971, found 489.1972.

### (*E*)-1-(1-(ethylthio)prop-1-*en*-2-yl)-2,5-diphenyl-4-(1-phenylvinyl)-1,2-dihydro-3*H*-pyrazol-3-one (5)

IR *ν*_max_ (neat)/cm^−1^: 3057, 2973, 2824, 1698, 1572, 1376, 1269, 1187, 1028, 932; ^1^H NMR (600 MHz, CDCl_3_): *δ* 7.58 (d, *J* = 7.2 Hz, 2H), 7.46 (t, *J* = 7.8 Hz, 2H), 7.33–7.30 (m, 5H), 7.26 (t, *J* = 7.2 Hz, 1H), 7.21 (t, *J* = 7.2 Hz, 2H), 7.16 (t, *J* = 7.2 Hz, 2H), 7.12 (t, *J* = 7.2 Hz, 1H), 6.34 (s, 1H), 5.69 (d, *J* = 1.2 Hz, 1H), 5.67 (d, *J* = 1.2 Hz, 1H), 2.53 (q, *J* = 7.2 Hz, 2H), 1.38 (s, 3H), 1.03 (t, *J* = 7.2 Hz, 3H); ^13^C{^1^H} NMR (150 MHz, CDCl_3_): *δ* 164.4, 155.5, 140.4, 138.6, 135.9, 133.3, 130.5, 129.8, 129.6, 129.5, 128.7, 127.9, 127.2, 127.1, 126.5, 124.7, 118.8, 114.8, 28.1, 15.4, 13.5. HRMS (ESI-TOF, *m*/*z*): calcd for C_28_H_27_N_2_OS^+^, [M + H]^+^, 439.1839, found 439.1838.

### Reaction of unsaturated pyrazolones 1a with trimethylsilyl propargyl sulfide ylide 2c

To a flame-dried sealable 3-dram vial equipped with a stir bar was added unsaturated pyrazolinones 1a (68 mg, 0.2 mmol, 1.0 equiv.), NaOAc·3H_2_O (14 mg, 0.1 mmol, 0.5 equiv.) and 2c (61 mg, 0.24 mmol, 1.2 equiv.) under air. Subsequently treated CH_3_CN (2 mL, *c* = 0.1 M) was added to vial *via* syringe. The reaction mixture was stirred for 15 min at 20 °C until unsaturated pyrazolones 1a was fully consumed (monitored by TLC). Then the reaction was stirred at 0 °C for 6 h. The organic solvent was removed under reduced pressure and purified through column chromatography (eluent: petroleum ether and EtOAc) to afford the desired product 3a (79% yield, 70 mg).

### Reaction of unsaturated pyrazolones 1a with methyl propargyl sulfide ylide 2d

To a flame-dried sealable 3-dram vial equipped with a stir bar was added unsaturated pyrazolinones 1a (68 mg, 0.2 mmol, 1.0 equiv.), Cs_2_CO_3_ (33 mg, 0.1 mmol, 0.5 equiv.) and 2d (47 mg, 0.24 mmol, 1.2 equiv.) under air. Subsequently treated CH_3_CN (2 mL, *c* = 0.1 M) was added to vial *via* syringe. The reaction mixture was stirred for 2 h at 20 °C until unsaturated pyrazolones 1a was fully consumed (monitored by TLC). Then the reaction was stirred at 0 °C for 15 h. The organic solvent was removed under reduced pressure and purified through column chromatography (eluent: petroleum ether and EtOAc) to afford the desired product 6 (36 mg, 62% yield, conversion: 74%).

### 1-(3-Methyl-4-(methylthio)but-1-*en*-2-yl)-2,5-diphenyl-4-(1-phenylvinyl)-1,2-dihydro-3*H*-pyrazol-3-one (6)

The reaction was carried out on a 0.2 mmol scale following the General procedure. The product 6 was purified through column chromatography (PE/EtOAc: from 6 : 1 to 4 : 1) and obtained as a foam solid (56 mg, 62% yield). IR *ν*_max_ (neat)/cm^−1^: 3062, 2933, 1668, 1574, 1379, 1259, 1149, 1028, 932, 834; ^1^H NMR (600 MHz, CDCl_3_): *δ* 7.63 (d, *J* = 7.2 Hz, 2H), 7.47 (t, *J* = 7.8 Hz, 2H), 7.36 (t, *J* = 7.2 Hz, 2H), 7.32 (t, *J* = 7.2 Hz, 2H), 7.29–7.22 (m, 4H), 7.17 (t, *J* = 7.2 Hz, 2H), 7.13 (t, *J* = 7.2 Hz, 1H), 5.68 (d, *J* = 1.2 Hz, 1H), 5.60 (d, *J* = 1.2 Hz, 1H), 5.51 (s, 1H), 5.10 (s, 1H), 2.19 (dd, *J* = 2.4, 13.2 Hz, 1H), 2.13–2.10 (m, 1H), 1.97 (dd, *J* = 10.2, 13.2 Hz, 1H), 1.89 (s, 3H), 0.81 (d, *J* = 6.6 Hz, 3H); ^13^C{^1^H} NMR (150 MHz, CDCl_3_): *δ* 164.8, 155.6, 150.6, 140.3, 138.4, 136.2, 130.4, 129.6, 129.3, 128.7, 128.0, 127.9, 127.3, 126.9, 126.3, 124.1, 123.2, 118.8, 117.0, 114.0, 40.4, 35.1, 18.8, 15.9. HRMS (ESI-TOF, *m*/*z*): calcd for C_29_H_28_N_2_NaOS^+^, [M + Na]^+^, 475.1815, found 475.1818.

### Reaction of unsaturated pyrazolones 1d with methyl propargyl sulfide ylide 2d

To a flame-dried sealable 3-dram vial equipped with a stir bar was added unsaturated pyrazolinones 1d (72 mg, 0.2 mmol, 1.0 equiv.), Cs_2_CO_3_ (33 mg, 0.1 mmol, 0.5 equiv.) under air. Subsequently treated CH_3_CN (2 mL, *c* = 0.1 M) was added to vial *via* syringe. The reaction mixture was stirred for 2 h at 20 °C until unsaturated pyrazolones 1d was fully consumed (monitored by TLC). Then the reaction was stirred at 0 °C for 15 h. The organic solvent was removed under reduced pressure and purified through column chromatography (eluent: petroleum ether and EtOAc) to afford the desired product 7 (65 mg, 68% yield, conversion: 81%).

### 4-(1-(4-Chlorophenyl)vinyl)-1-(3-methyl-4-(methylthio)but-1-*en*-2-yl)-2,5-diphenyl-1,2-dihydro-3*H*-pyrazol-3-one (7)

The reaction was carried out on a 0.2 mmol scale. The product 7 was purified through column chromatography (PE/EtOAc: from 6 : 1 to 4 : 1) and obtained as a foam solid (64 mg, 66% yield). IR *ν*_max_ (neat)/cm^−1^: 3086, 2982, 1688, 1559, 1437, 1322, 1251, 1067, 972, 853; ^1^H NMR (600 MHz, CDCl_3_): *δ* 7.61 (d, *J* = 7.8 Hz, 2H), 7.47 (t, *J* = 7.8 Hz, 2H), 7.33 (d, *J* = 7.2 Hz, 2H), 7.32–7.30 (m, 2H), 7.25 (d, *J* = 7.2 Hz, 2H), 7.22 (t, *J* = 7.2 Hz, 2H), 7.12 (d, *J* = 8.4 Hz, 2H), 5.65 (s, 1H), 5.64 (s, 1H), 5.50 (s, 1H), 5.10 (s, 1H), 2.18 (dd, *J* = 1.8, 7.2 Hz, 1H), 2.12–2.08 (m, 1H), 1.97 (dd, *J* = 10.8, 13.2 Hz, 1H), 1.88 (s, 3H), 0.81 (t, *J* = 6.6 Hz, 3H); ^13^C{^1^H} NMR (150 MHz, CDCl_3_): *δ* 164.5, 155.5, 150.4, 138.9, 137.4, 136.1, 133.2, 130.3, 129.8, 129.1, 128.8, 128.3, 128.1, 128.0, 126.5, 124.1, 119.3, 117.1, 113.3, 40.4, 35.3, 18.7, 15.9. HRMS (ESI-TOF, *m*/*z*): calcd for C_29_H_27_ClN_2_NaOS^+^, [M + Na]^+^, 509.1425, found 509.1425.

### Gram-scale synthesis of 3a

To a flame-dried sealable 3-dram vial equipped with a stir bar was added unsaturated pyrazolinones 1a (1.01 g, 3.0 mmol, 1.0 equiv.), NaOAc·3H_2_O (204 mg, 1.5 mmol, 0.5 equiv.) and 2a (0.65 g, 3.6 mmol, 1.2 equiv.) under air. Subsequently treated CH_3_CN (25 mL, *c* = 0.12 M) was added to vial *via* syringe. The reaction mixture was stirred for 20 min at 20 °C until unsaturated pyrazolones 1a was fully consumed (monitored by TLC). Then the reaction was stirred at 0 °C for 10 h. The organic solvent was removed under reduced pressure and purified through column chromatography (eluent: petroleum ether and EtOAc) to afford the desired product 3a with a yield of 79% (1.01 g).

### Oxidation of 3a with *m*-CPBA

To a flame-dried sealable 2-dram vial equipped with a stir bar was added pyrazolones 3a (302 mg, 0.68 mmol, 1.0 equiv.), Na_2_CO_3_ (145 mg, 1.36 mmol, 3 equiv.) and CH_2_Cl_2_ (5 mL, *c* = 0.14 M). After stirred at 0 °C for 10 min, *m*-CPBA (313 mg, 1.36 mmol, 75%, 2.0 equiv.) was added to the mixture slowly. The reaction mixture was kept stirring for 1 min at 0 °C until unsaturated pyrazolones 3a was fully consumed (monitored by TLC). The reaction was quenched with aqueous Na_2_CO_3_ and extracted with CH_2_Cl_2_ (5 mL × 3). The combined organic solvent was dried with anhydrous Na_2_SO_4_, removed under reduced pressure and purified through column chromatography (eluent: petroleum ether and EtOAc) to afford the desired product 8 (101 mg, 33%) and 9 (125 mg, 39%).

### 1-(4-(Methylsulfinyl)but-1-*en*-2-yl)-2,5-diphenyl-4-(1-phenylvinyl)-1,2-dihydro-3*H*-pyrazol-3-one (8)

IR *ν*_max_ (neat)/cm^−1^: 3162, 2958, 1672, 1587, 1421, 1356, 1151, 1077, 946; ^1^H NMR (600 MHz, CDCl_3_): *δ* 7.60 (d, *J* = 7.8 Hz, 2H), 7.46 (t, *J* = 7.2 Hz, 2H), 7.31 (d, *J* = 7.2 Hz, 3H), 7.29–7.24 (m, 3H), 7.21 (t, *J* = 7.2 Hz, 2H), 7.13 (t, *J* = 7.2 Hz, 2H), 7.10 (t, *J* = 7.2 Hz, 1H), 5.68 (s, 2H), 5.52 (s, 1H), 5.11 (s, 1H), 2.41 (t, *J* = 6.6 Hz, 2H), 2.36 (s, 3H), 2.22 (t, *J* = 6.6 Hz, 2H); ^13^C{^1^H} NMR (150 MHz, CDCl_3_): *δ* 164.6, 155.5, 143.9, 140.1, 138.2, 135.8, 129.9, 129.8, 128.8, 128.1, 128.0, 127.9, 127.2, 126.9, 126.5, 124.1, 119.1, 114.5, 51.5, 38.4, 23.9. HRMS (ESI-TOF, *m*/*z*): calcd for C_28_H_26_N_2_NaO_2_S^+^, [M + Na]^+^, 477.1607, found 477.1609.

### 1-(4-(Methylsulfonyl)but-1-*en*-2-yl)-2,5-diphenyl-4-(1-phenylvinyl)-1,2-dihydro-3*H*-pyrazol-3-one (9)

IR *ν*_max_ (neat)/cm^−1^: 3055, 2985, 1663, 1578, 1412, 1365, 1155, 1062, 964; ^1^H NMR (600 MHz, CDCl_3_): *δ* 7.61 (d, *J* = 1.2 Hz, 2H), 7.60 (d, *J* = 1.2 Hz, 2H), 7.48 (t, *J* = 7.8 Hz, 3H), 7.34–7.27 (m, 3H), 7.23 (t, *J* = 7.2 Hz, 2H), 7.15 (t, *J* = 7.2 Hz, 2H), 7.12 (t, *J* = 7.2 Hz, 1H), 5.70 (s, 2H), 5.54 (s, 1H), 5.09 (d, *J* = 0.6 Hz, 1H), 2.73 (t, *J* = 8.4 Hz, 2H), 2.68 (s, 3H), 2.29 (t, *J* = 8.4 Hz, 2H); ^13^C{^1^H} NMR (150 MHz, CDCl_3_): *δ* 164.7, 155.5, 143.2, 140.1, 138.1, 135.8, 130.0, 129.0, 128.2, 128.0, 127.4, 127.0, 126.8, 124.1, 119.3, 115.1, 52.4, 40.5, 24.0. HRMS (ESI-TOF, *m*/*z*): calcd for C_28_H_26_N_2_NaO_3_S^+^, [M + Na]^+^, 493.1556, found 493.1558.

## Conflicts of interest

The authors declare no competing financial interest.

## Supplementary Material

RA-009-C9RA07610G-s001

RA-009-C9RA07610G-s002
