# Supplementary material for: Combined inorganic base promoted N-addition/[2,3]-sigmatropic rearrangement to construct homoallyl sulfur-containing pyrazolones
Source: RSC Adv. 2019 Oct 29;9(60):34912–25. doi: 10.1039/c9ra07610g (PMC9082452; doi:10.1039/c9ra07610g)
Supplement: RA-009-C9RA07610G-s001 [file RA-009-C9RA07610G-s001.pdf]

Electronic Supplementary Material (ESI) for RSC Advances.
This journal is © The Royal Society of Chemistry 2019

Combined Inorganic Bases Promoted N-Addition/[2,3]-Sigmatropic Rearrangement to Construct Homoallyl Sulfur-Containing Pyrazolones

Shou-Jie Shen,†*[a] Xiao-Li Du,†[a] Xiao-Li Xu,‡[b] Yue-Hua Wu,‡[b] Ming-gang Zhao,†[a] and Jin-Yan Liang‡*[b]

†*Key Laboratory of Magnetic Molecules, Magnetic Information Materials Ministry of Education, The School of Chemical and Material Science, Shanxi Normal University, Linfen, 041004, China*

‡College of Life Science, Shanxi Normal University, Linfen, 041004, China

E-mail: shoujie_shen@outlook.com, jinyan_liang@outlook.com

Supporting Material

A. X-ray Structures of 3a……………………………………………………………S2-S3

B. NMR Spectra………………………………………………………………..………S4-S45

A. X-Ray of 3a

Crystal of compound **3a** was obtained by dissolving product in *n*-Hexane and Ethyl acetate (v/v 5:1) and allowing the solvent to slowly evaporate at room temperature. The single crystal X-ray diffraction study at room temperature revealed that compound **3a** ($C_{28}H_{26}N_2OS$) crystallizes as the centrosymmetric monoclinic space group Pbca (Z = 8) and the crystal structure consists of one crystallographically independent formula unit in the unit cell (Figure S1).

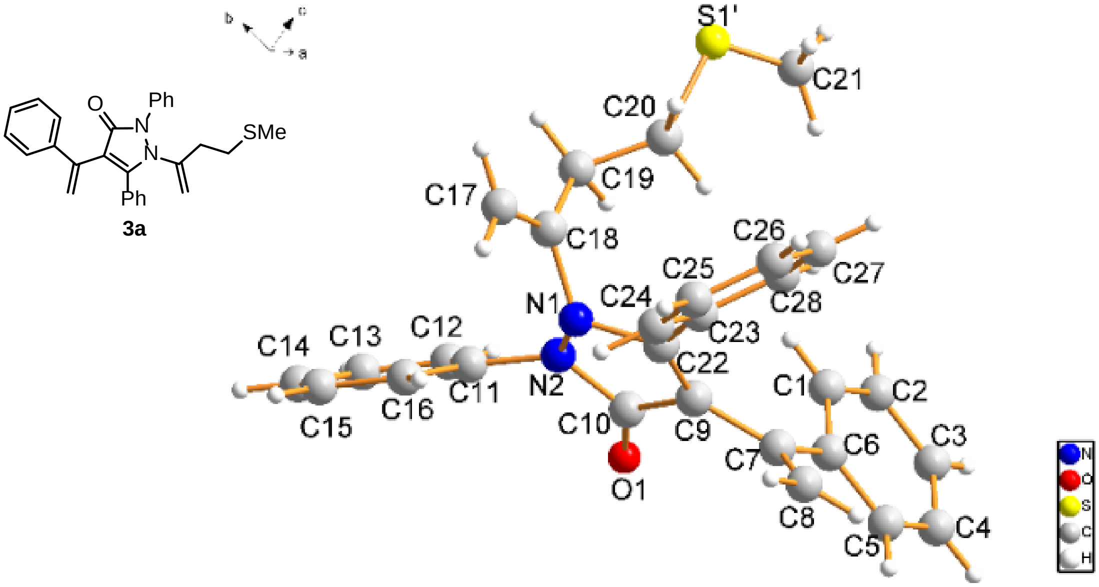

Figure S1. X-ray crystallography structure of **3a**. Drawn with ellipsoids at 30% probability and hydrogen atoms omitted for clarity.

Table S1. X-ray crystallography data of **3a**

Moiety formula	$C_{28}H_{26}N_2OS$
Mr	438.57
Dx	1.219 g/cm^{-3}
Wavelength	0.71000
Cell	a = 12.3067 (3) alpha = 90<br>b = 18.4393 (4) beta = 90<br>c = 21.0597 (5) gamma = 90
Temperature	293 K
Volume	4779.00 (19)
Space group	Pbca
Hall group	-P 2ac 2ab
Z	8
Mu	0.158/mm^{-1}
F000/F000’	1856.0/1857.64
T_{min}, T_{max}, $T_{min'}$	0.940, 0.947, 0.939
Data completeness	0.999
Theta (max)	25.998
R (reflections)	0.0669 (3339)
wR_2 (reflections)	0.2060 (4705)
S	1.057
Npar	317

B. NMR Spectra

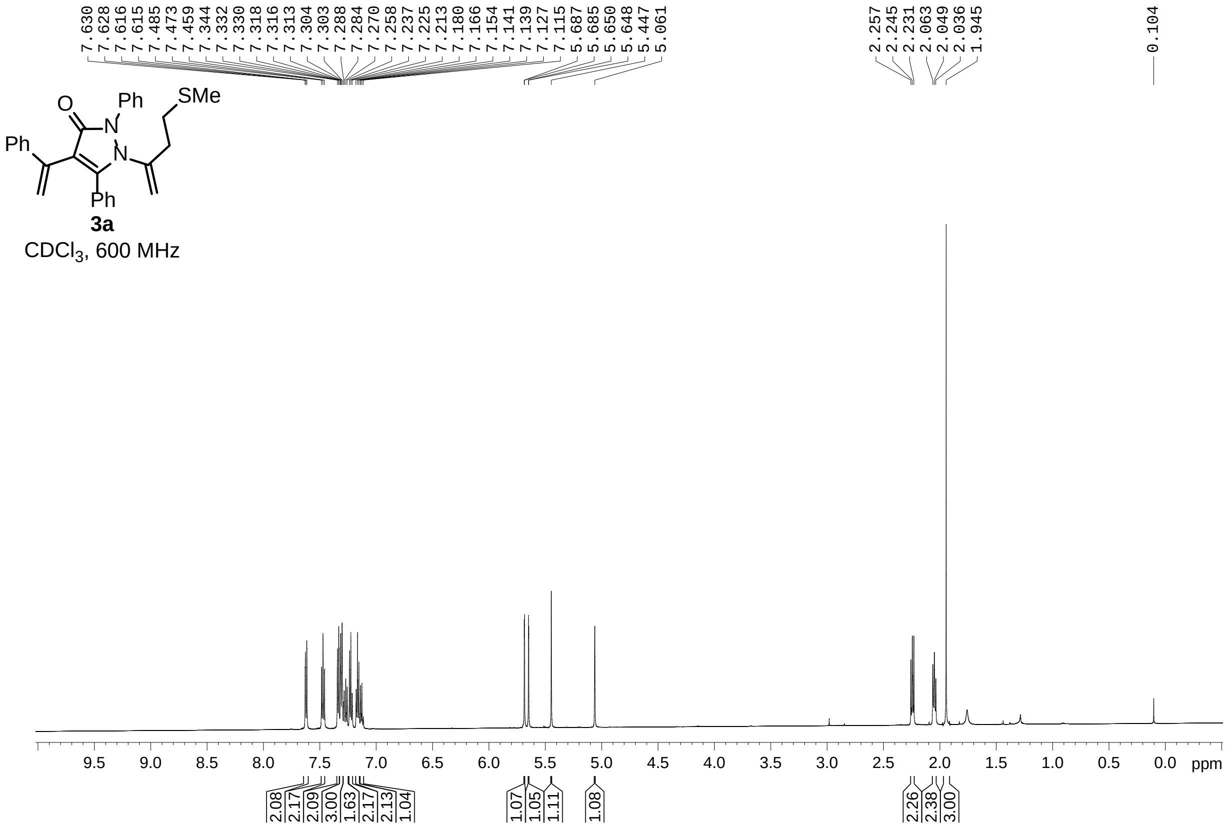

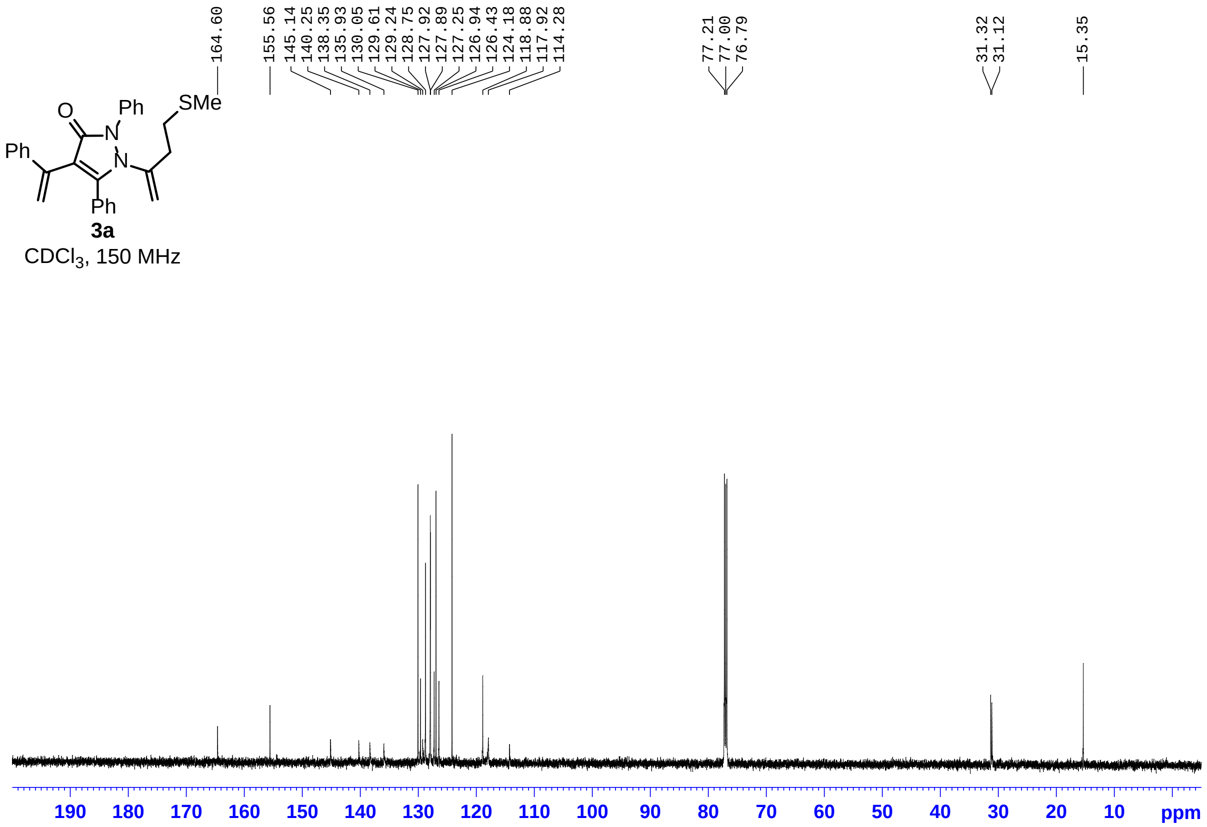

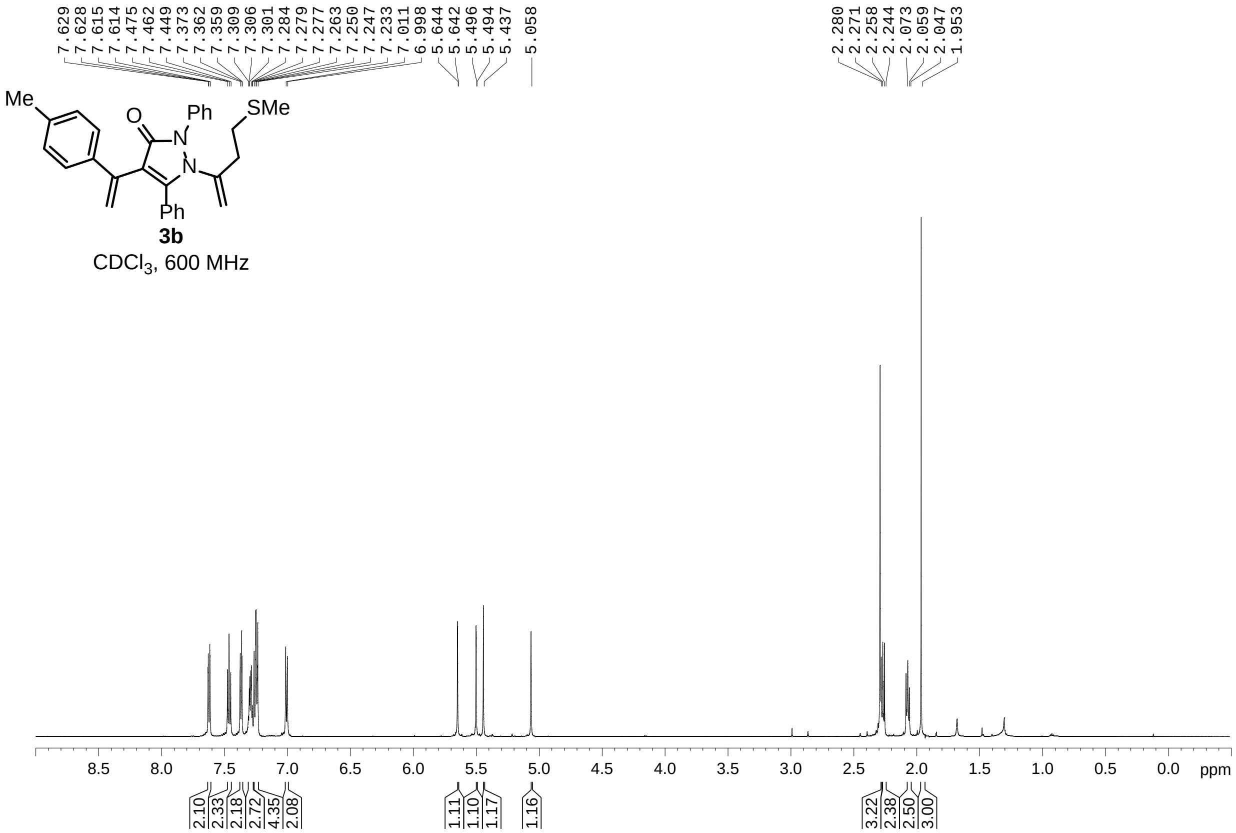
7.629 7.628 7.615 7.614 7.475 7.462 7.449 7.373 7.362 7.359 7.309 7.306 7.301 7.284 7.279 7.277 7.263 7.250 7.247 7.233 7.011 6.998 5.644 5.642 5.496 5.494 5.437 5.058
2.280 2.271 2.258 2.244 2.073 2.059 2.047 1.953
Me
O
N
Ph
SMe
N
Ph
3b
CDCl3, 600 MHz
8.5 8.0 7.5 7.0 6.5 6.0 5.5 5.0 4.5 4.0 3.5 3.0 2.5 2.0 1.5 1.0 0.5 0.0 ppm
2.10 2.33 2.18 2.72 4.35 2.08 1.11 1.10 1.17 1.16 3.22 2.38 2.50 3.00

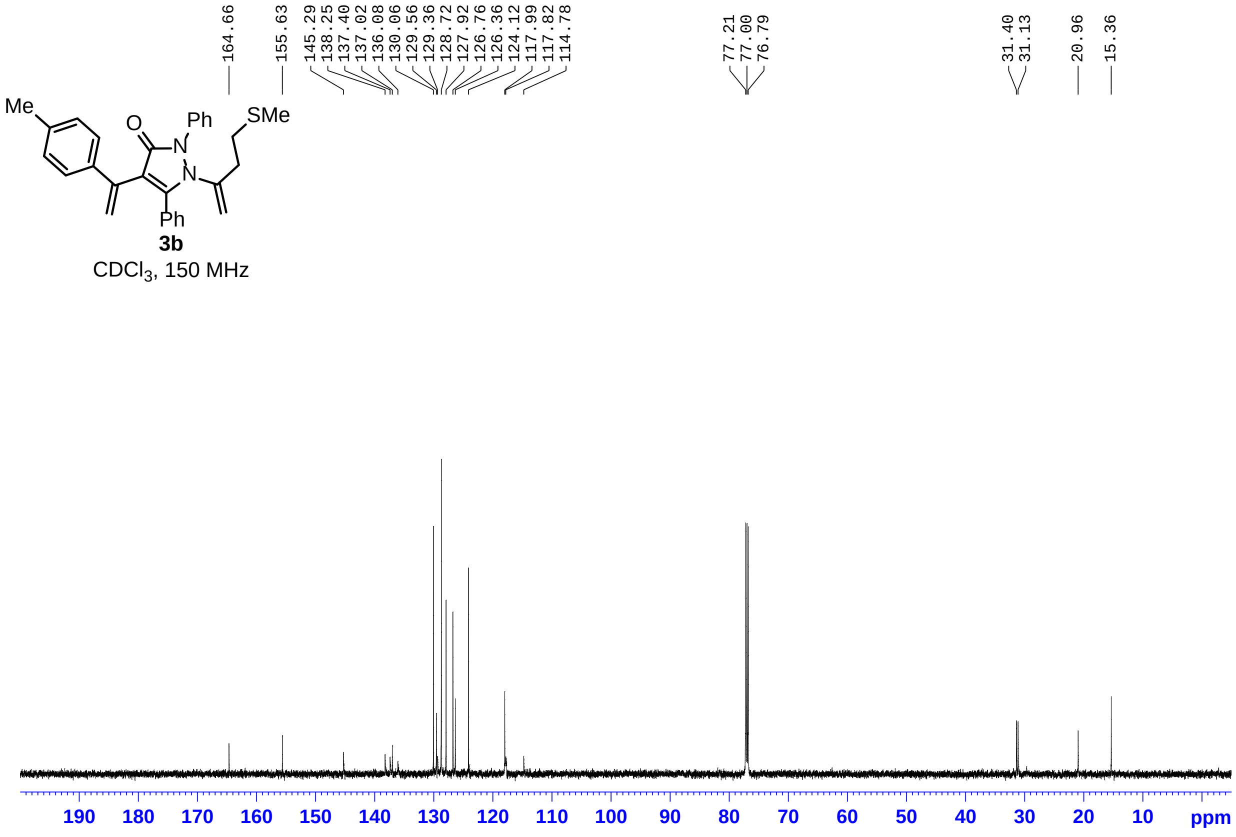
164.66 155.63 145.29 138.25 137.40 137.02 136.08 130.06 129.56 129.36 128.72 127.92 126.76 126.36 124.12 117.99 117.82 114.78
77.21 77.00 76.79
31.40 31.13 20.96 15.36
Me
O
N
Ph
SMe
N
Ph
3b
CDCl3, 150 MHz
190 180 170 160 150 140 130 120 110 100 90 80 70 60 50 40 30 20 10 ppm

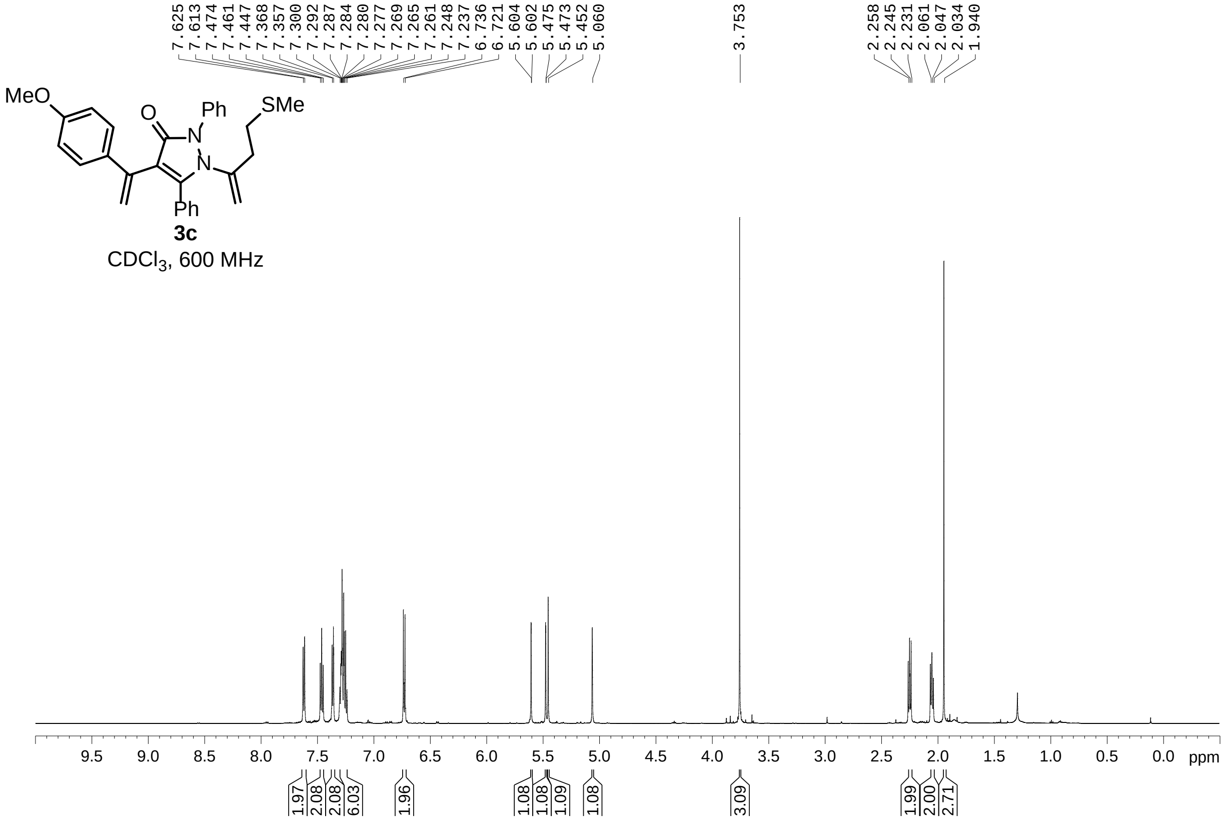

3c

$CDCl_3$, 600 MHz

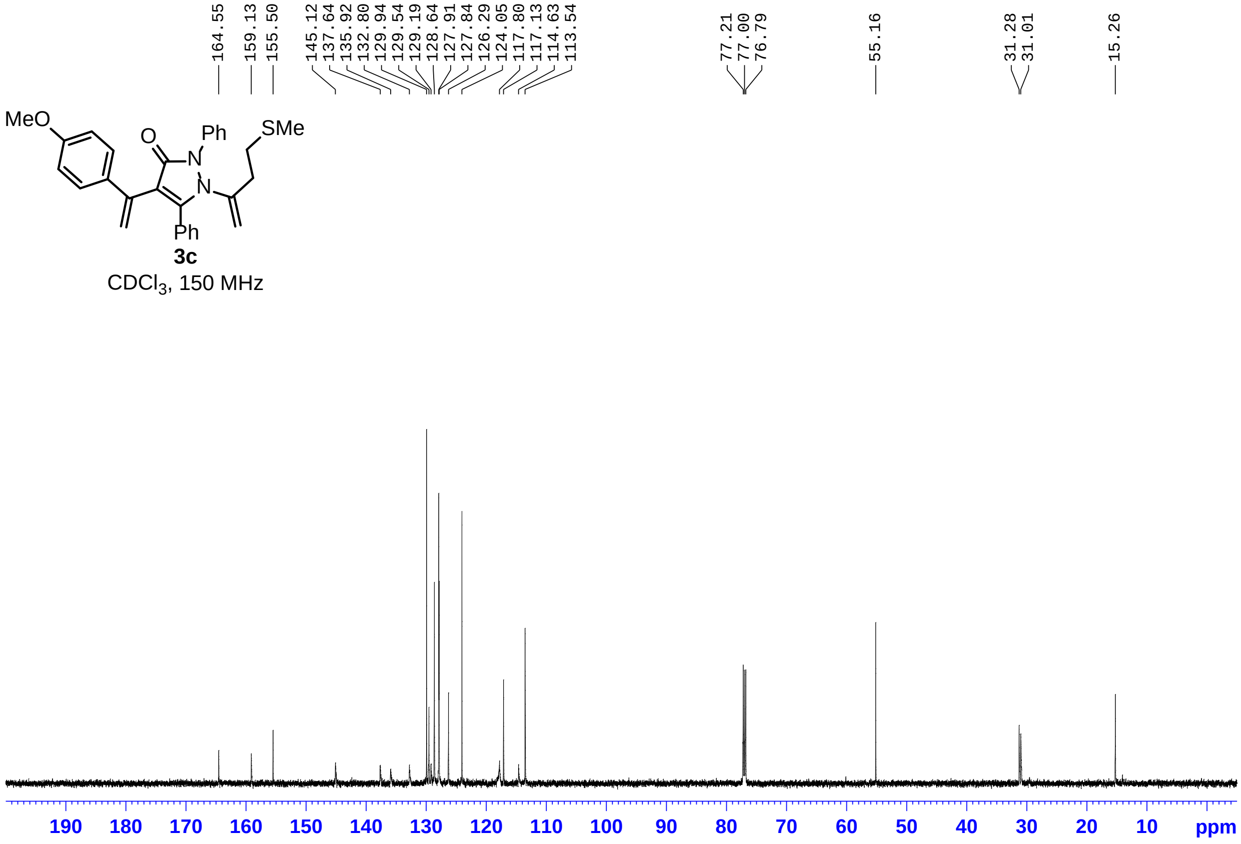

3c

$CDCl_3$, 150 MHz

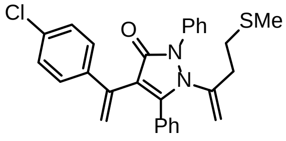

3d

$CDCl_3$, 600 MHz

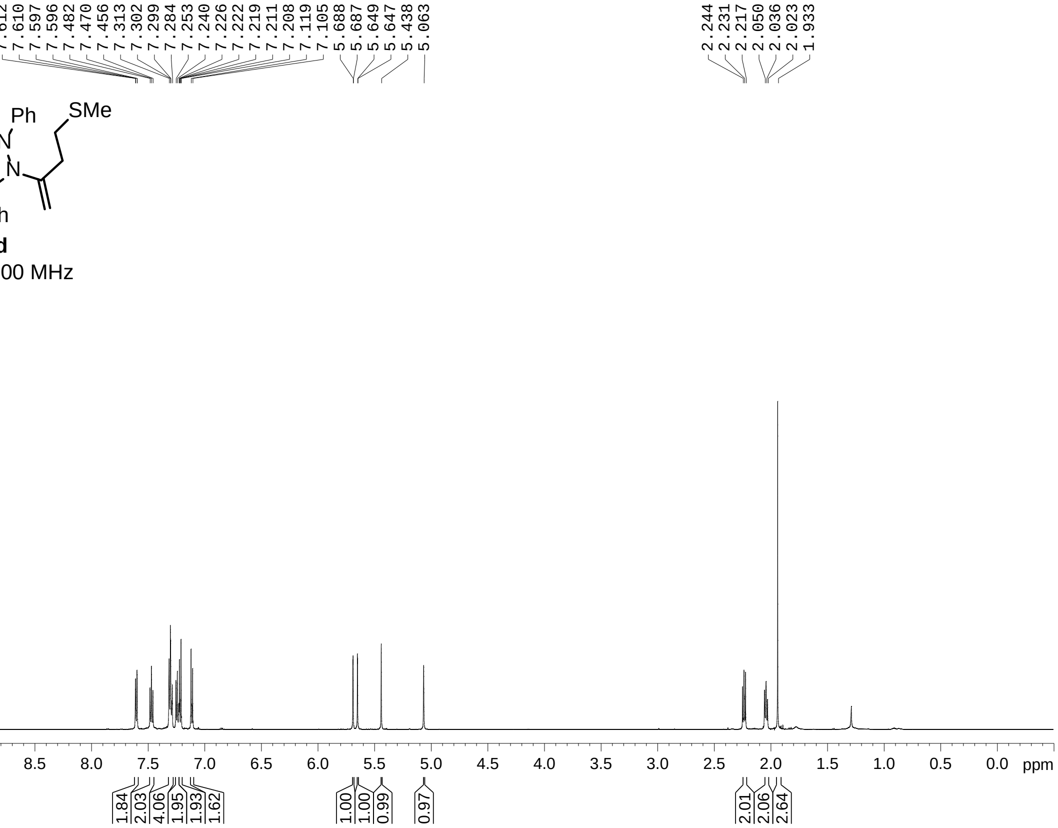

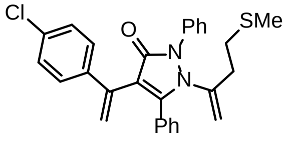

3d

$CDCl_3$, 150 MHz

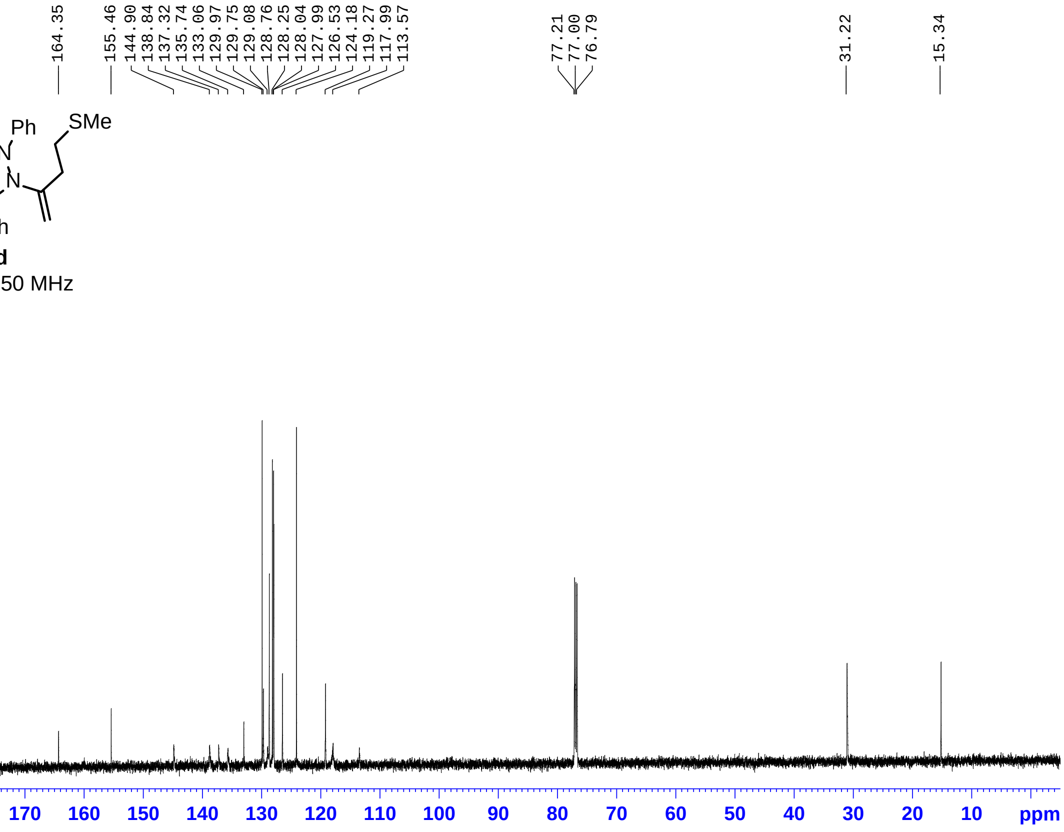

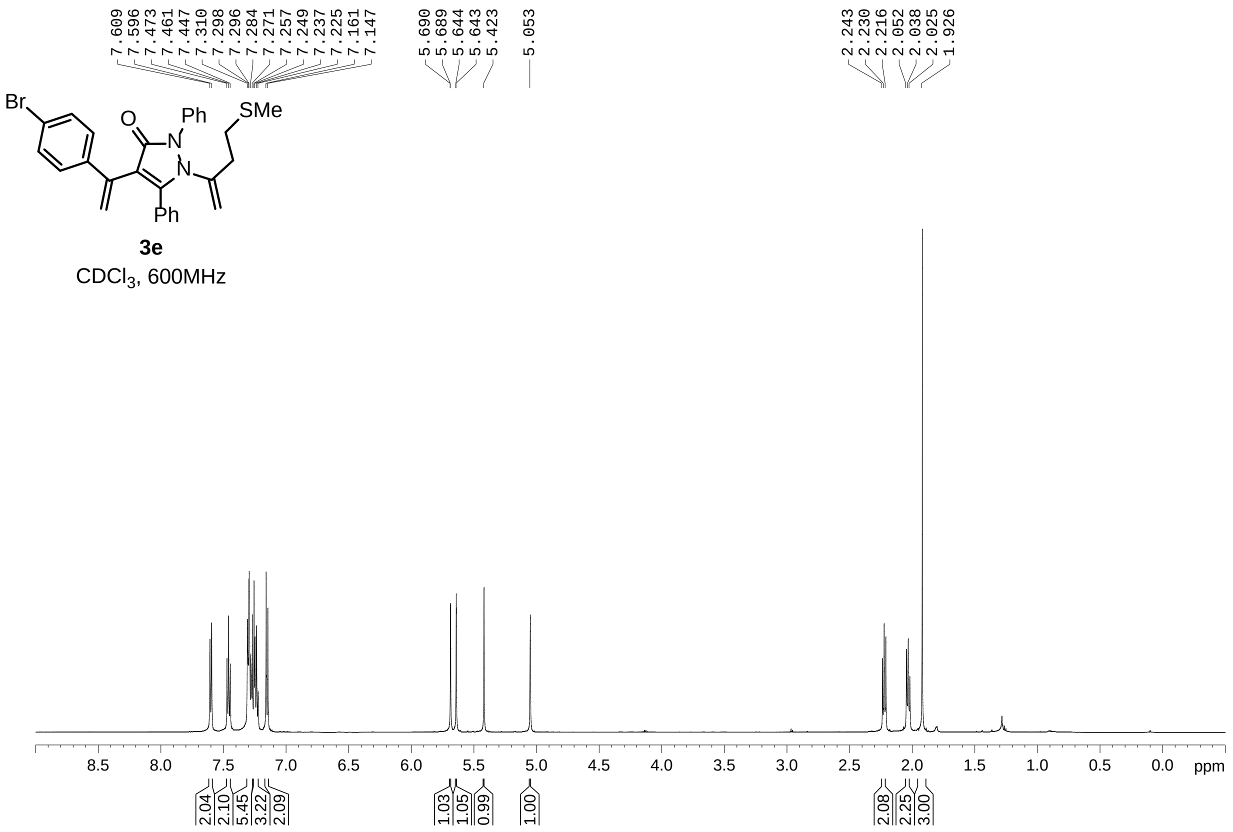

3e

$CDCl_3$, 600MHz

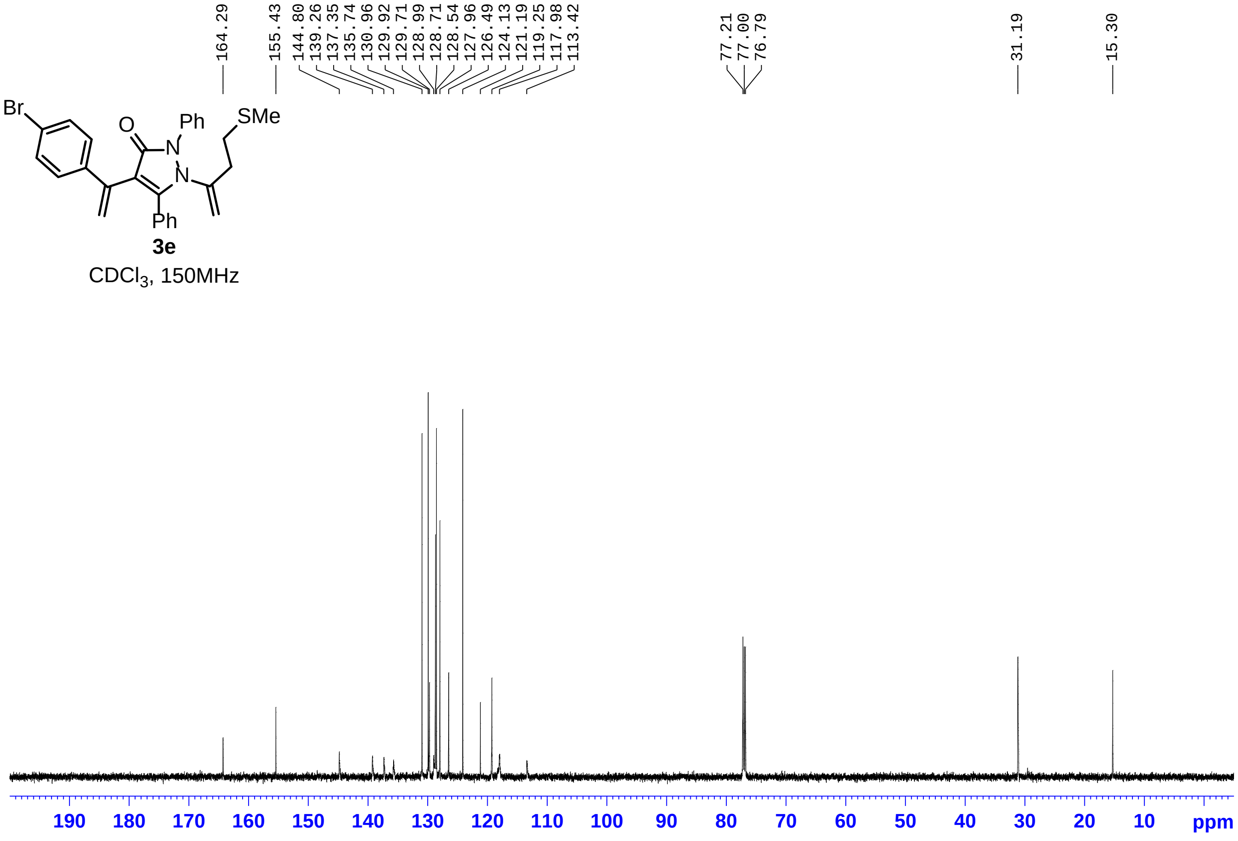

3e

$CDCl_3$, 150MHz

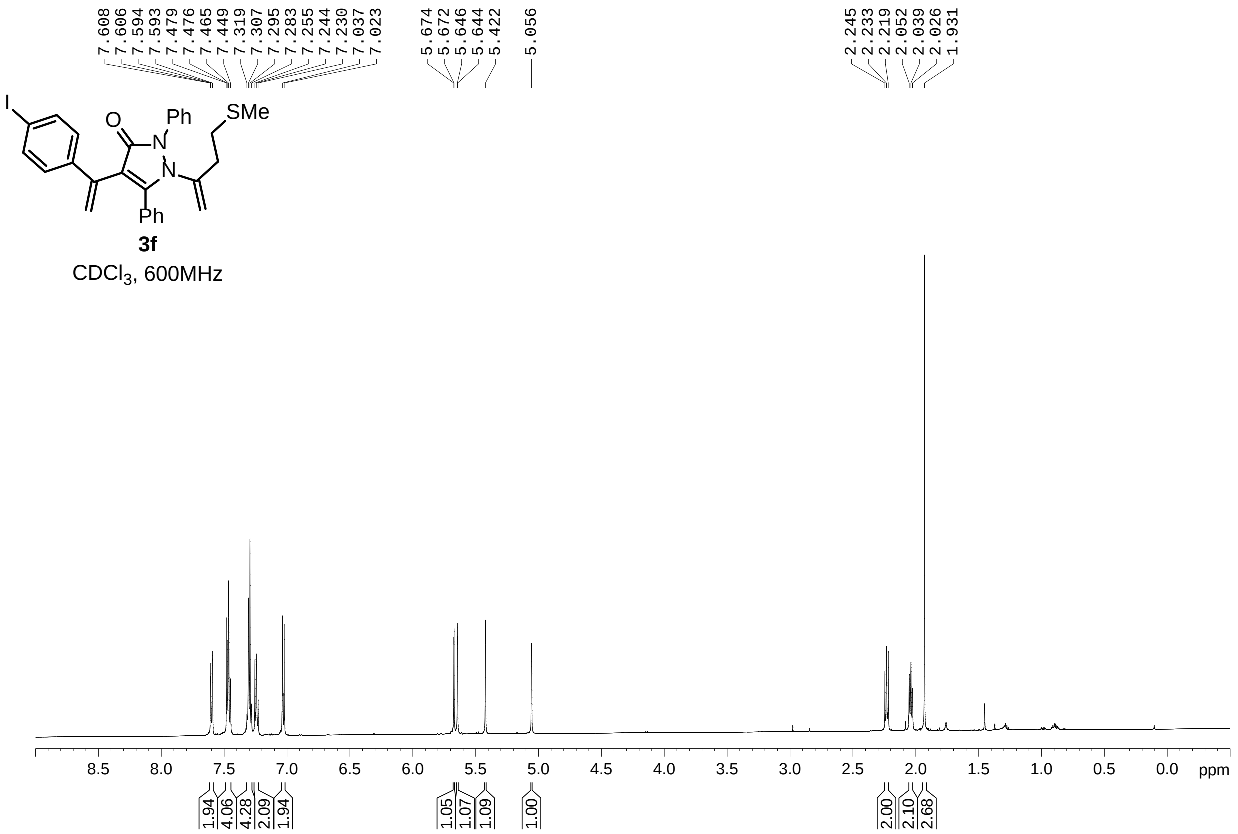
7.608
7.606
7.594
7.593
7.479
7.476
7.465
7.449
7.319
7.307
7.295
7.283
7.255
7.244
7.230
7.037
7.023
5.674
5.672
5.646
5.644
5.422
5.056
2.245
2.233
2.219
2.052
2.039
2.026
1.931
I
O
Ph
N
N
SMe
Ph
3f
CDCl3, 600MHz
8.5
8.0
7.5
7.0
6.5
6.0
5.5
5.0
4.5
4.0
3.5
3.0
2.5
2.0
1.5
1.0
0.5
0.0
ppm
1.94
4.06
4.28
2.09
1.94
1.05
1.07
1.09
1.00
2.00
2.10
2.68

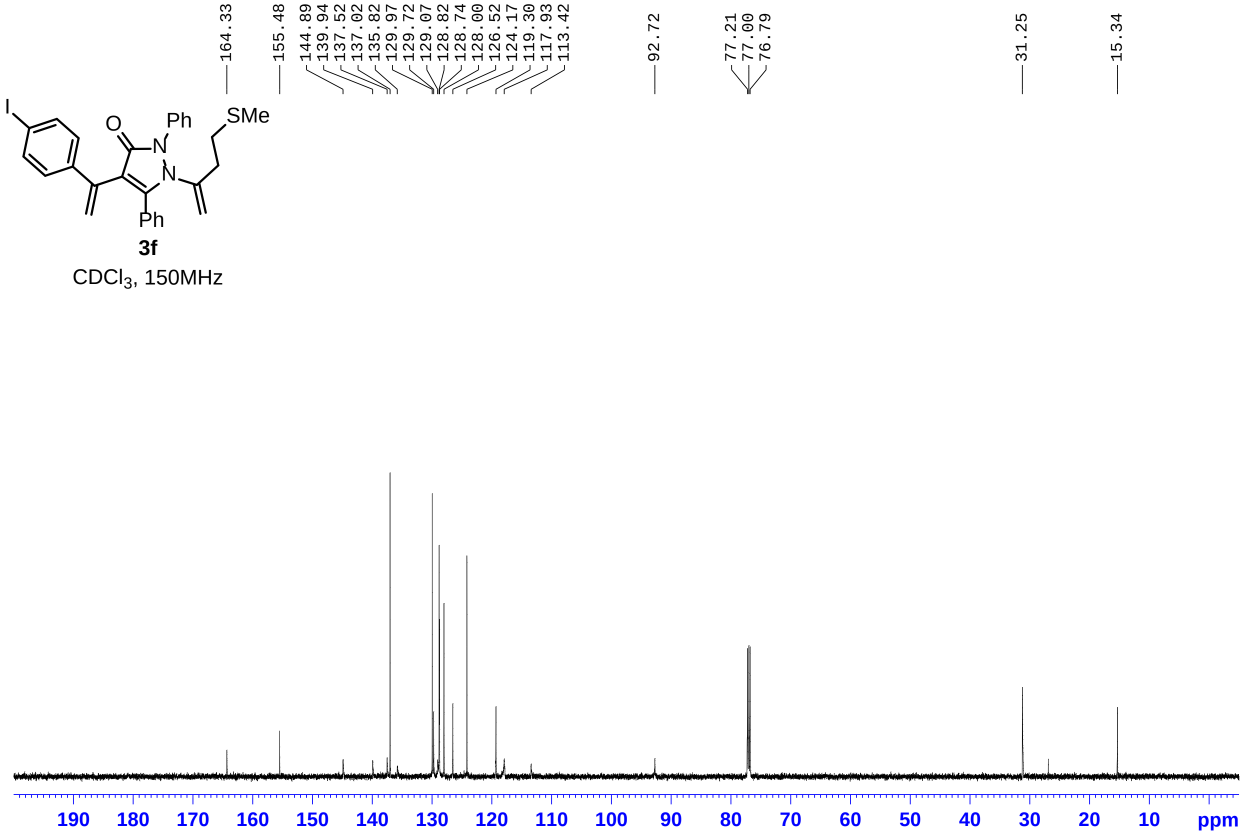
164.33
155.48
144.89
139.94
137.52
137.02
135.82
129.97
129.72
129.07
128.82
128.74
128.00
126.52
124.17
119.30
117.93
113.42
92.72
77.21
77.00
76.79
31.25
15.34
I
O
Ph
N
N
SMe
Ph
3f
CDCl3, 150MHz
190
180
170
160
150
140
130
120
110
100
90
80
70
60
50
40
30
20
10
ppm

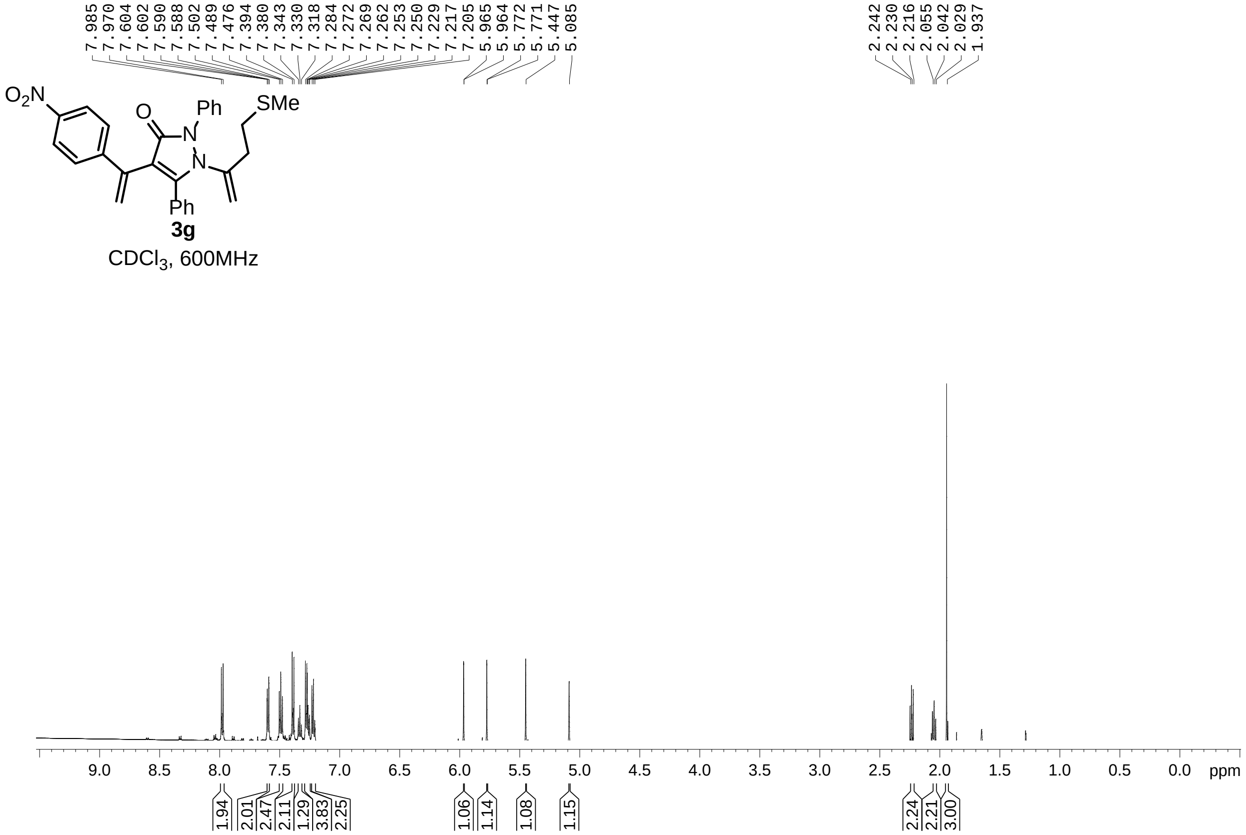
7.985 7.970 7.604 7.602 7.590 7.588 7.502 7.489 7.476 7.394 7.380 7.343 7.330 7.318 7.284 7.272 7.269 7.262 7.253 7.250 7.229 7.217 7.205 5.965 5.964 5.772 5.771 5.447 5.085
2.242 2.230 2.216 2.055 2.042 2.029 1.937
O_2N
O
N
Ph
N
SMe
Ph
3g
$CDCl_3$, 600MHz
9.0 8.5 8.0 7.5 7.0 6.5 6.0 5.5 5.0 4.5 4.0 3.5 3.0 2.5 2.0 1.5 1.0 0.5 0.0 ppm
1.94 2.01 2.47 2.11 1.29 3.83 2.25 1.06 1.14 1.08 1.15 2.24 2.21 3.00

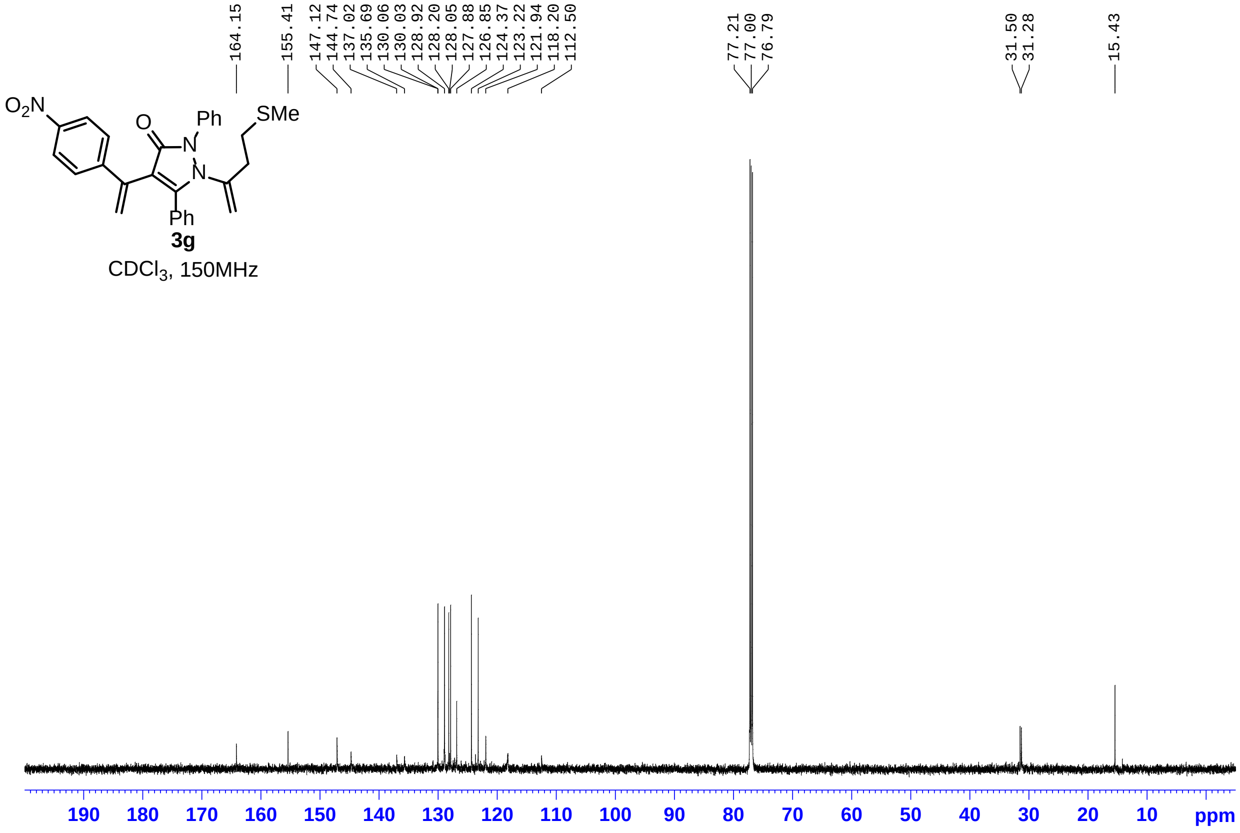
164.15 155.41 147.12 144.74 137.02 135.69 130.06 130.03 128.92 128.20 128.05 127.88 126.85 124.37 123.22 121.94 118.20 112.50
77.21 77.00 76.79
31.50 31.28
15.43
O_2N
O
N
Ph
N
SMe
Ph
3g
$CDCl_3$, 150MHz
190 180 170 160 150 140 130 120 110 100 90 80 70 60 50 40 30 20 10 ppm

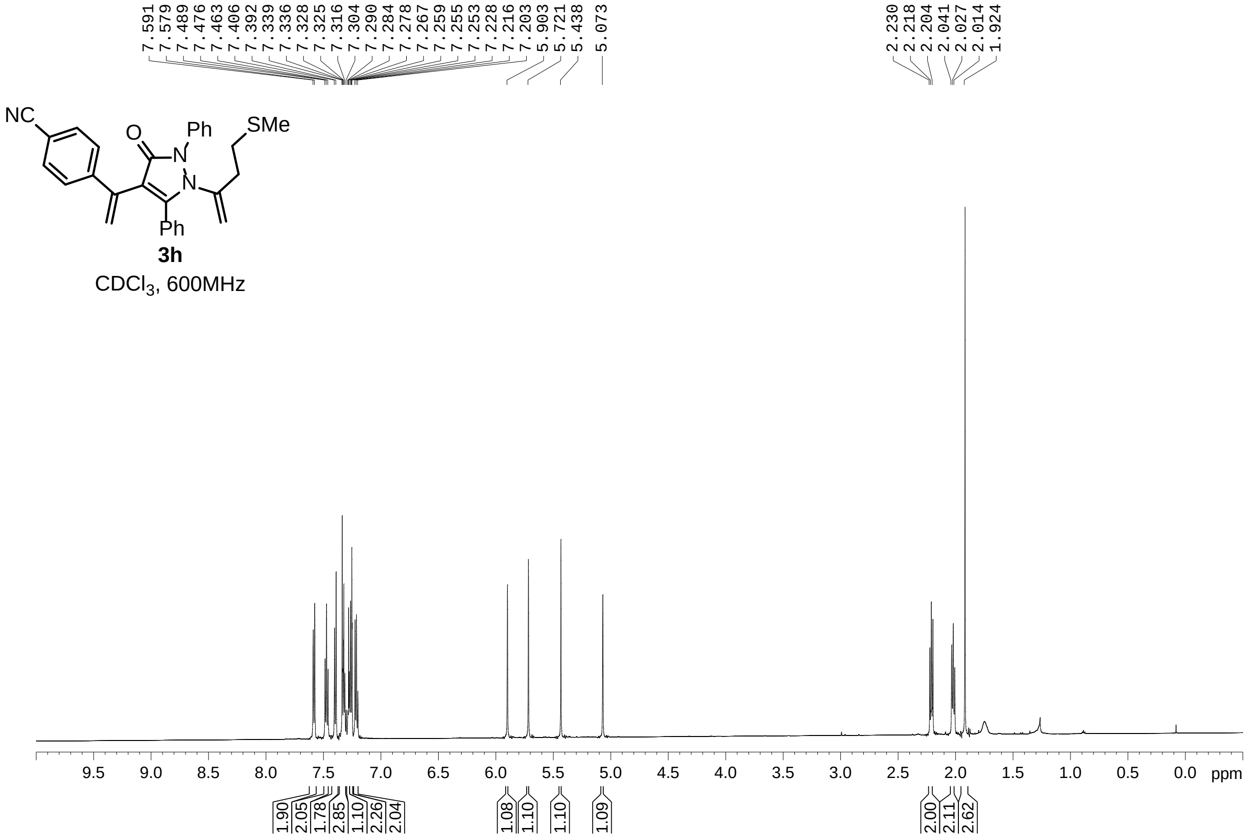

7.591
7.579
7.489
7.476
7.463
7.406
7.392
7.339
7.336
7.328
7.325
7.316
7.304
7.290
7.284
7.278
7.267
7.259
7.255
7.253
7.228
7.216
7.203
5.903
5.721
5.438
5.073
2.230
2.218
2.204
2.041
2.027
2.014
1.924
NC
O
N
Ph
SMe
N
Ph
3h
CDCl3, 600MHz
9.5 9.0 8.5 8.0 7.5 7.0 6.5 6.0 5.5 5.0 4.5 4.0 3.5 3.0 2.5 2.0 1.5 1.0 0.5 0.0 ppm
1.90
2.05
1.78
2.85
1.10
2.26
2.04
1.08
1.10
1.10
1.09
2.00
2.11
2.62

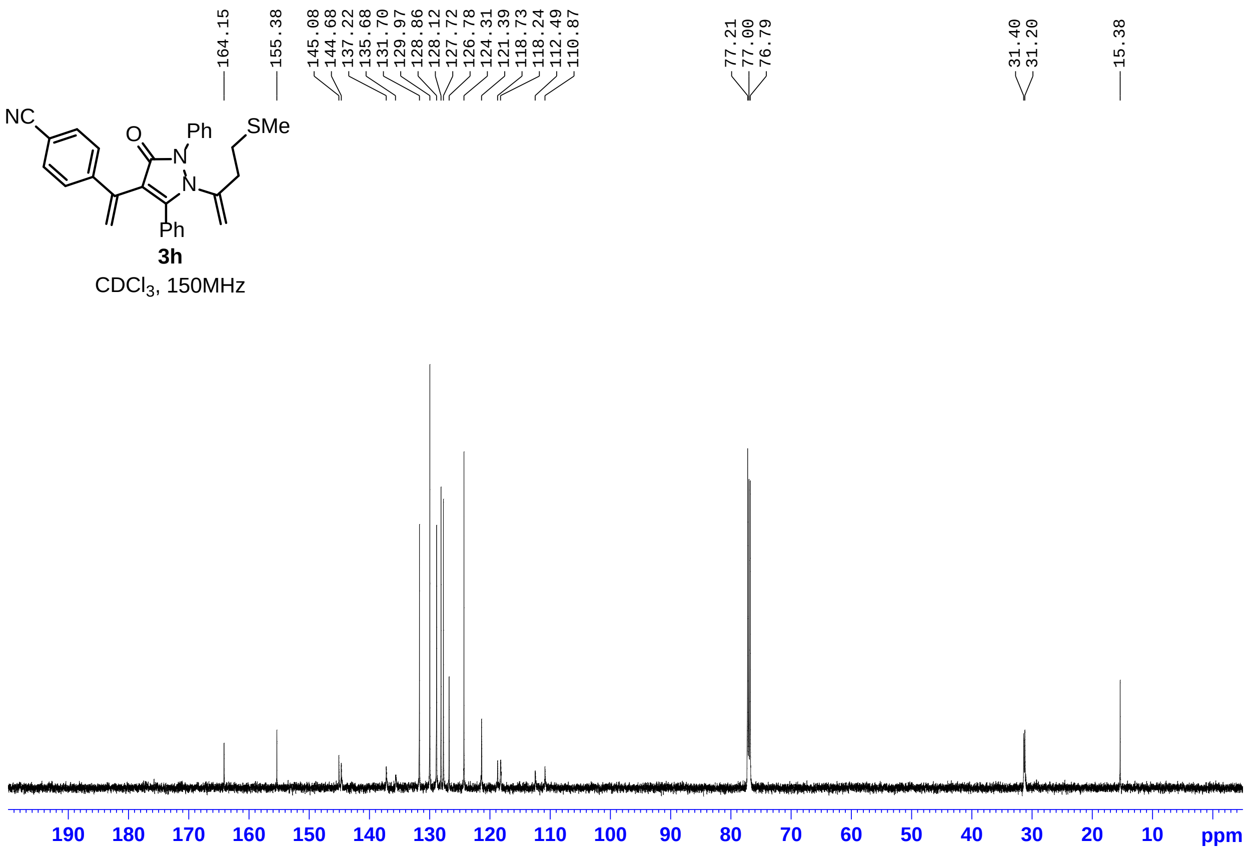

164.15
155.38
145.08
144.68
137.22
135.68
131.70
129.97
128.86
128.12
127.72
126.78
124.31
121.39
118.73
118.24
112.49
110.87
77.21
77.00
76.79
31.40
31.20
15.38
NC
O
N
Ph
SMe
N
Ph
3h
CDCl3, 150MHz
190 180 170 160 150 140 130 120 110 100 90 80 70 60 50 40 30 20 10 ppm

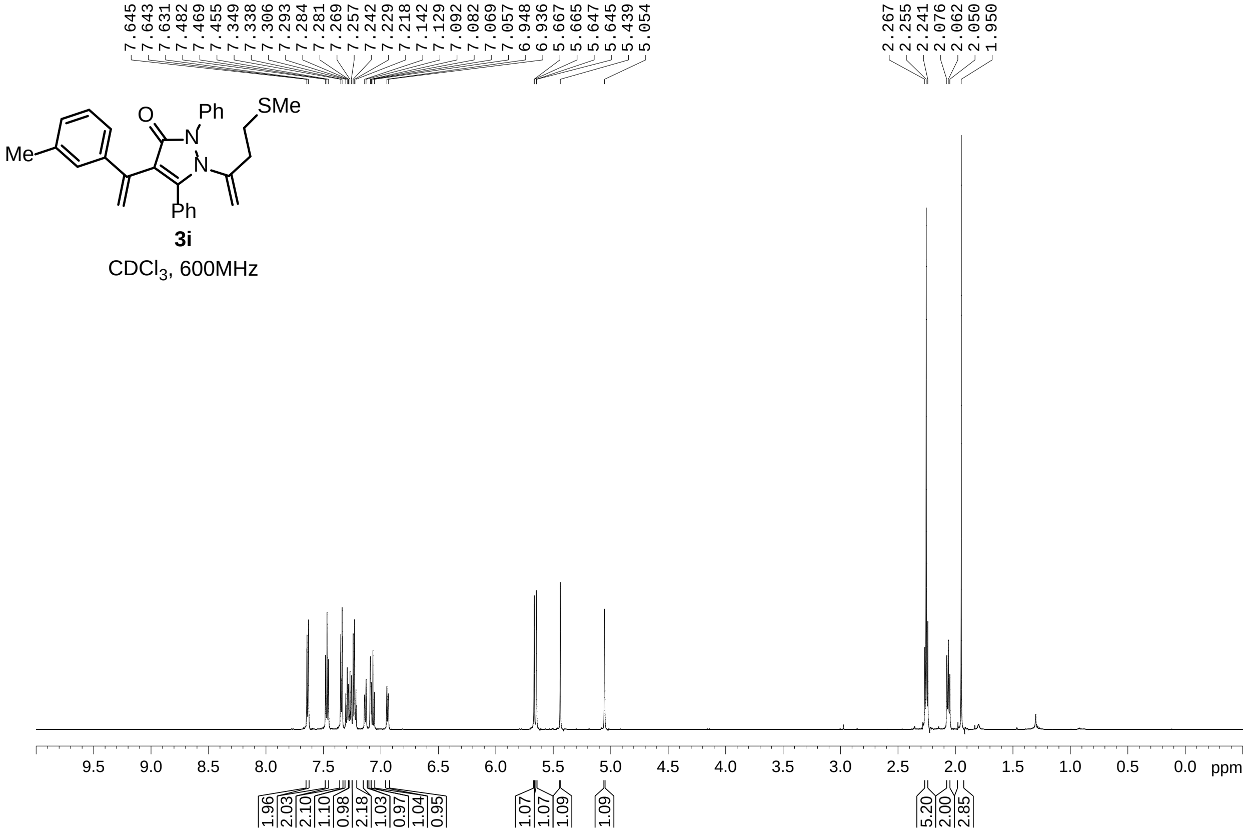
7.645 7.643 7.631 7.482 7.469 7.455 7.349 7.338 7.306 7.293 7.284 7.281 7.269 7.257 7.242 7.229 7.218 7.142 7.129 7.092 7.082 7.069 7.057 6.948 6.936 5.667 5.665 5.647 5.645 5.439 5.054
2.267 2.255 2.241 2.076 2.062 2.050 1.950
Me
O
Ph
SMe
N
N
Ph
3i
CDCl3, 600MHz
1.96 2.03 2.10 1.10 0.98 2.18 1.03 0.97 1.04 0.95
1.07 1.07 1.09
1.09
5.20 2.00 2.85
ppm

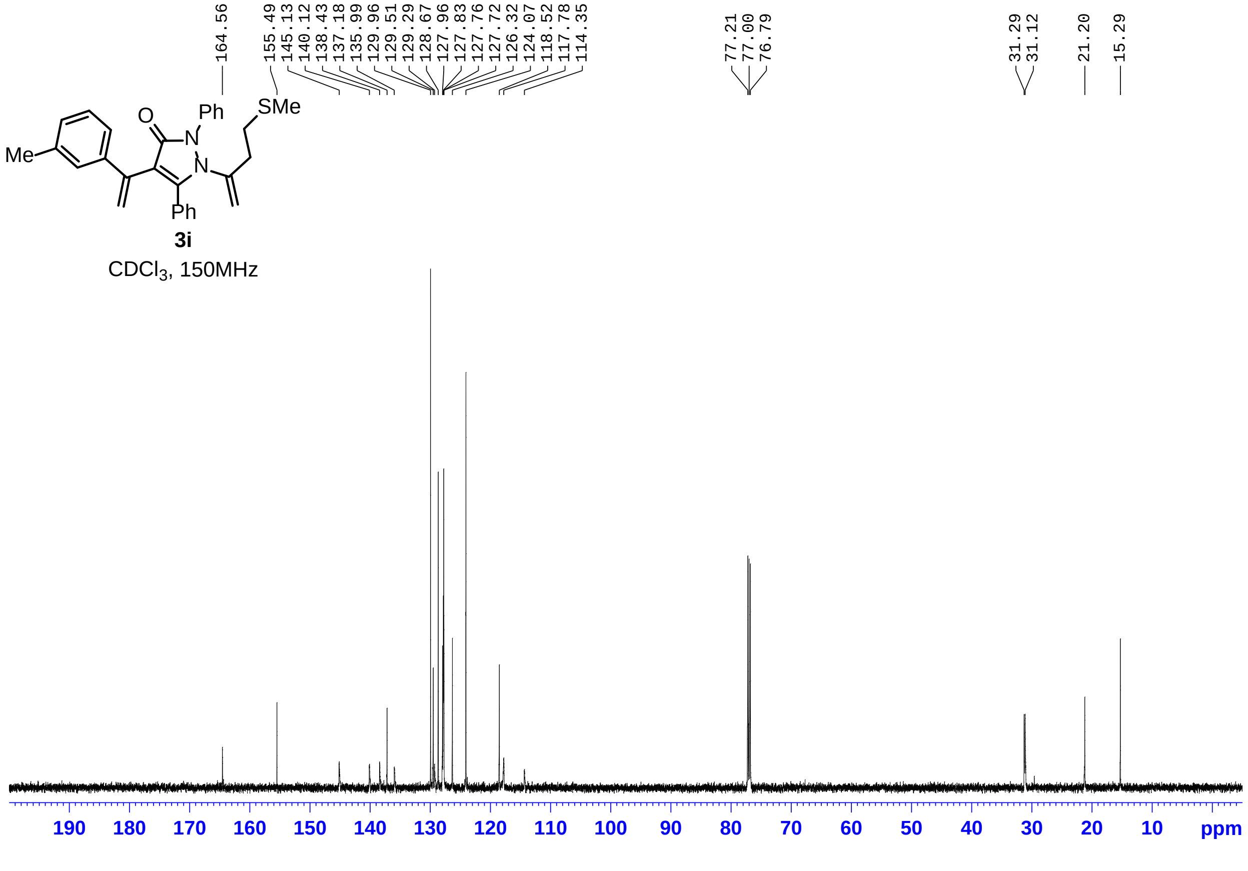
164.56
155.49 145.13 140.12 138.43 137.18 135.99 129.96 129.51 129.29 128.67 127.96 127.83 127.76 127.72 126.32 124.07 118.52 117.78 114.35
77.21 77.00 76.79
31.29 31.12
21.20
15.29
Me
O
Ph
SMe
N
N
Ph
3i
CDCl3, 150MHz
ppm

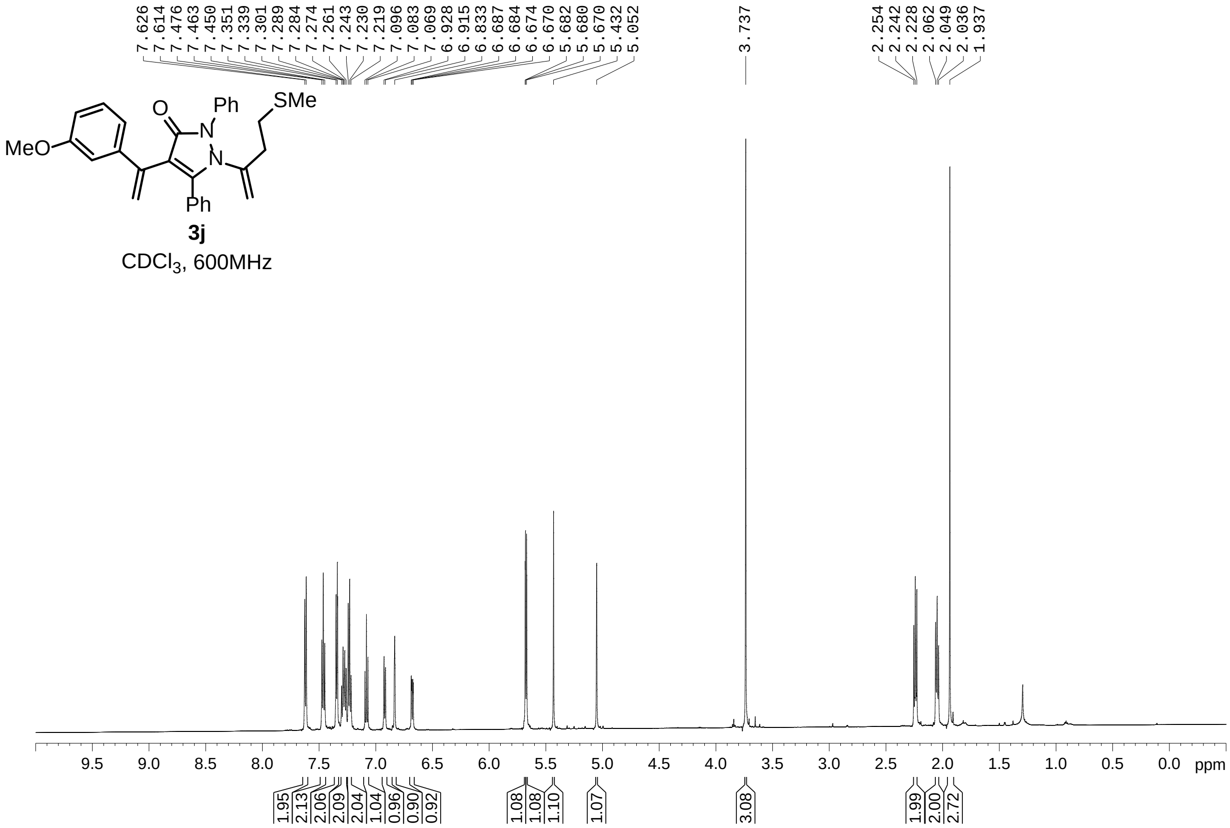
7.626 7.614 7.476 7.463 7.450 7.351 7.339 7.301 7.289 7.284 7.274 7.261 7.243 7.230 7.219 7.096 7.083 7.069 6.928 6.915 6.833 6.687 6.684 6.674 6.670 5.682 5.680 5.670 5.432 5.052
3.737
2.254 2.242 2.228 2.062 2.049 2.036 1.937
MeO
O
Ph
N
N
SMe
Ph
3j
CDCl3, 600MHz
9.5 9.0 8.5 8.0 7.5 7.0 6.5 6.0 5.5 5.0 4.5 4.0 3.5 3.0 2.5 2.0 1.5 1.0 0.5 0.0 ppm
1.95 2.13 2.06 2.09 2.04 1.04 0.96 0.90 0.92
1.08 1.08 1.10
1.07
3.08
1.99 2.00 2.72

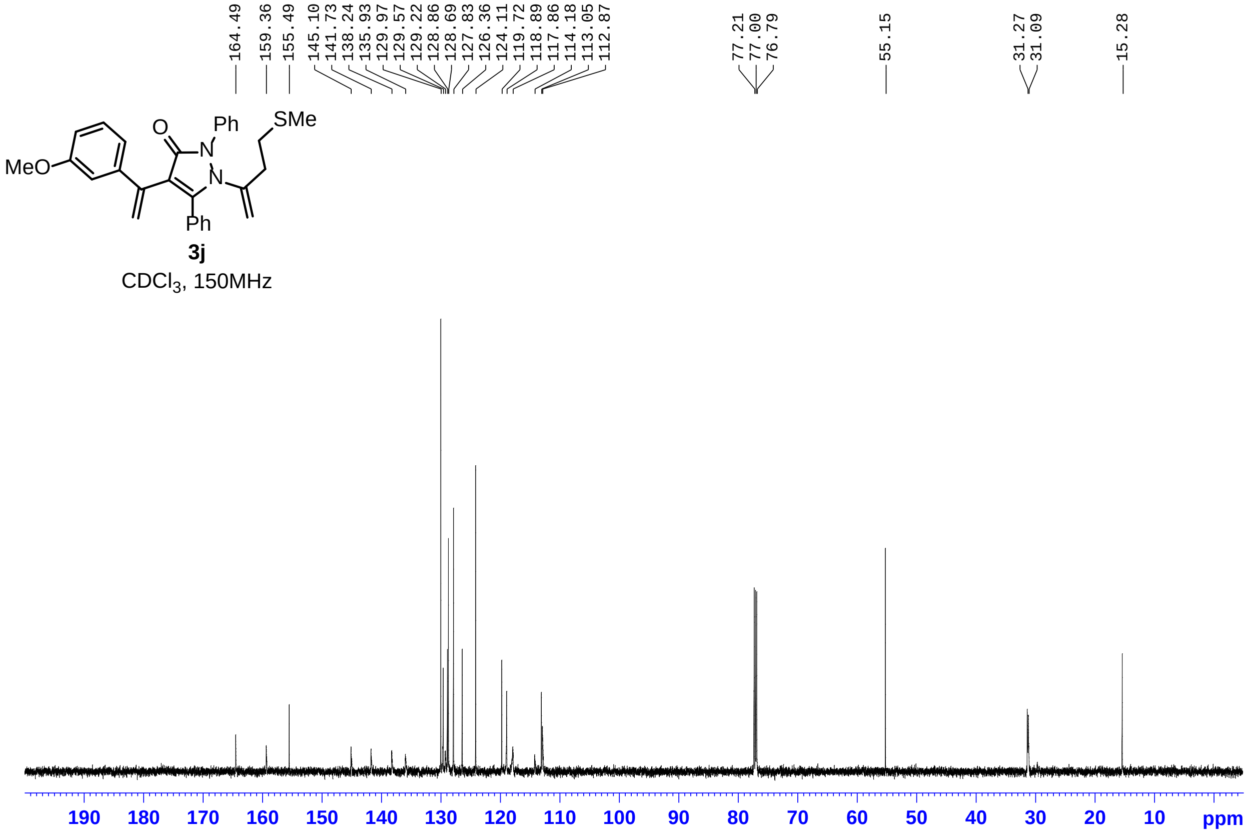
164.49 159.36 155.49 145.10 141.73 138.24 135.93 129.97 129.57 129.22 128.86 128.69 127.83 126.36 124.11 119.72 118.89 117.86 114.18 113.05 112.87
77.21 77.00 76.79
55.15
31.27 31.09
15.28
MeO
O
Ph
N
N
SMe
Ph
3j
CDCl3, 150MHz
190 180 170 160 150 140 130 120 110 100 90 80 70 60 50 40 30 20 10 ppm

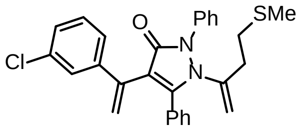

3k

$CDCl_3$, 600MHz

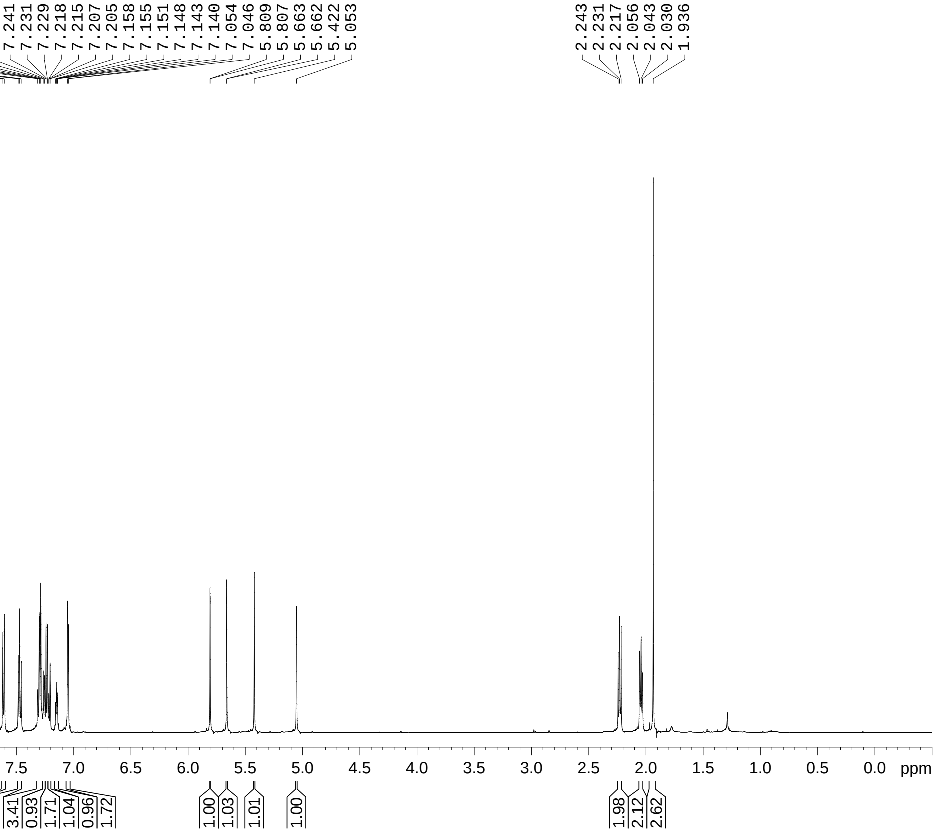

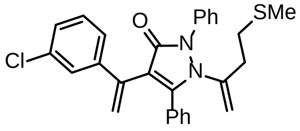

3k

$CDCl_3$, 150MHz

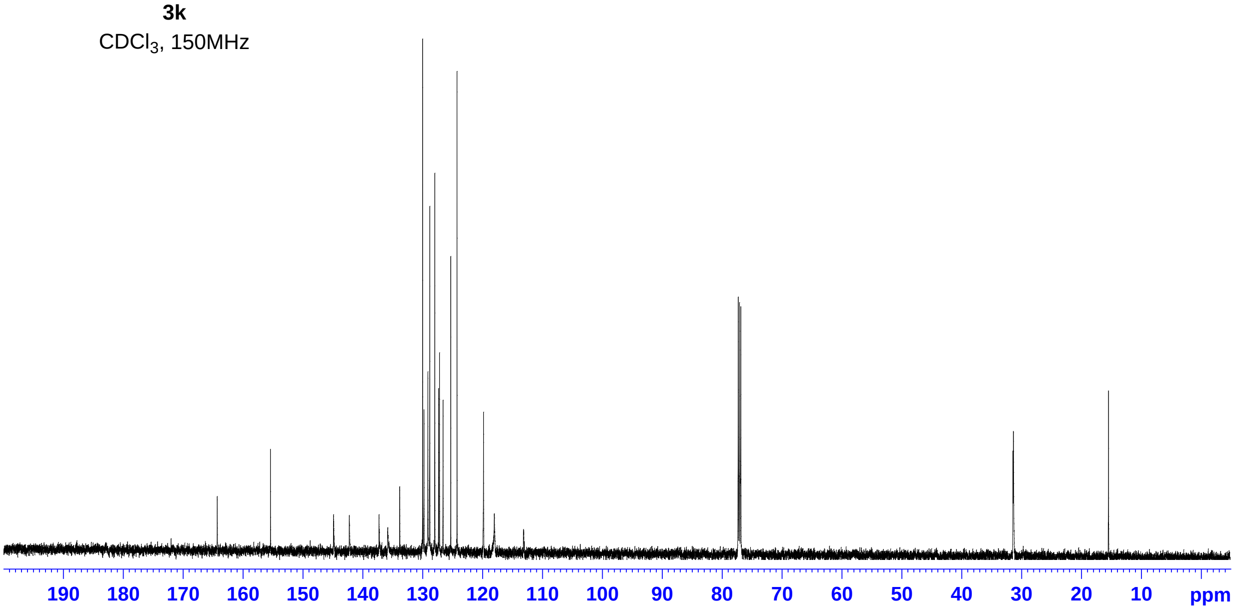

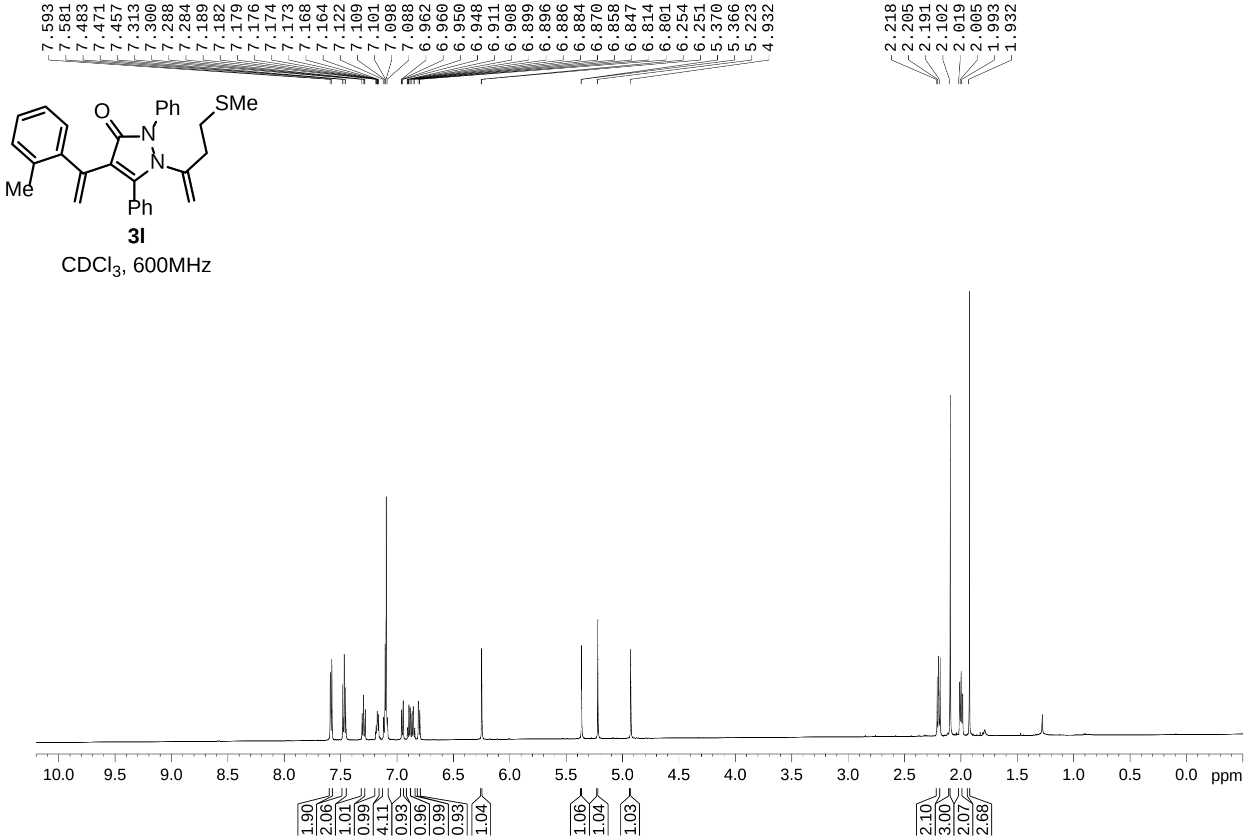
7.593 7.581 7.483 7.471 7.457 7.313 7.300 7.288 7.284 7.189 7.182 7.179 7.176 7.174 7.173 7.168 7.164 7.122 7.109 7.101 7.098 7.088 6.962 6.960 6.950 6.948 6.911 6.908 6.899 6.896 6.886 6.884 6.870 6.858 6.847 6.814 6.801 6.254 6.251 5.370 5.366 5.223 4.932
2.218 2.205 2.191 2.102 2.019 2.005 1.993 1.932
O
N
Ph
SMe
N
Me
Ph
3l
CDCl3, 600MHz
1.90 2.06 1.01 0.99 4.11 0.93 0.96 0.99 0.93 1.04 1.06 1.04 1.03 2.10 3.00 2.07 2.68
10.0 9.5 9.0 8.5 8.0 7.5 7.0 6.5 6.0 5.5 5.0 4.5 4.0 3.5 3.0 2.5 2.0 1.5 1.0 0.5 0.0 ppm

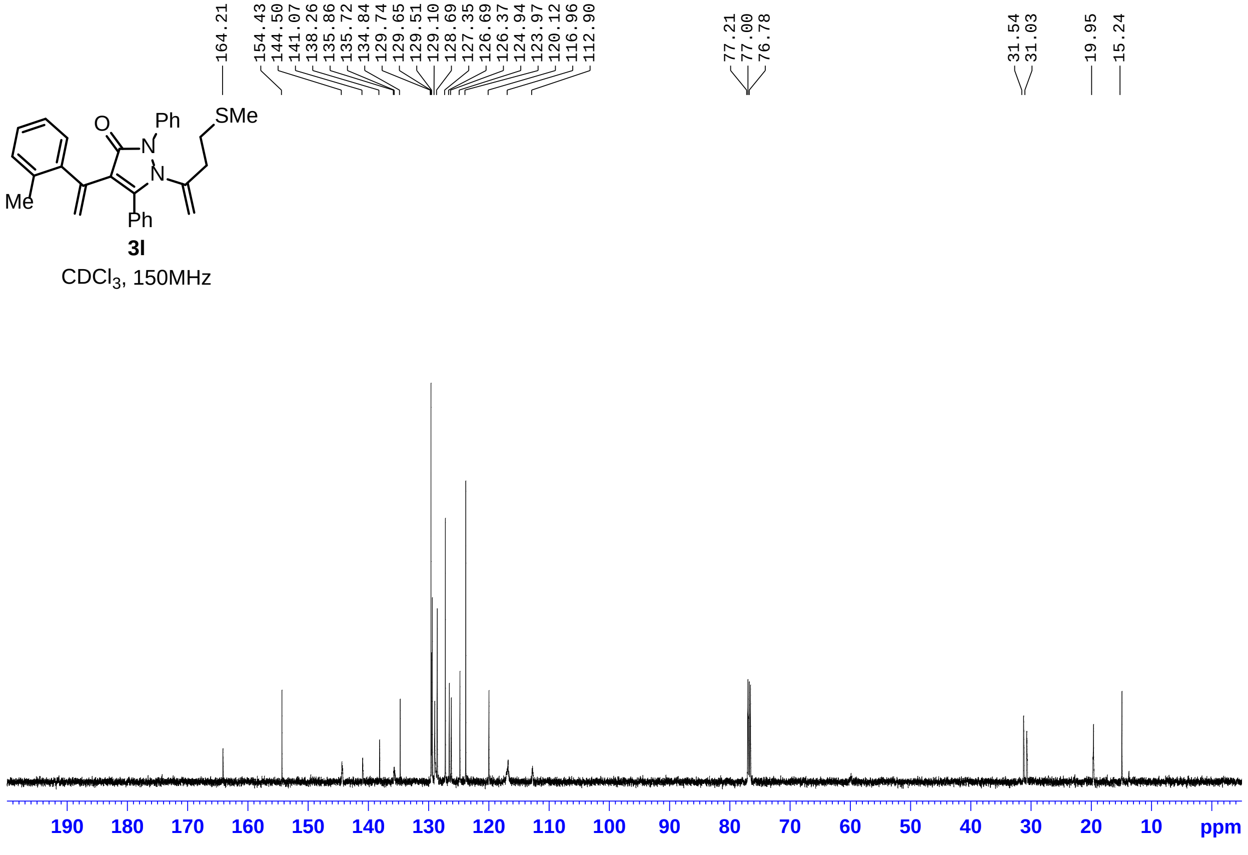
164.21
154.43 144.50 141.07 138.26 135.86 135.72 134.84 129.74 129.65 129.51 129.10 128.69 127.35 126.69 126.37 124.94 123.97 120.12 116.96 112.90
77.21 77.00 76.78
31.54 31.03
19.95
15.24
O
N
Ph
SMe
N
Me
Ph
3l
CDCl3, 150MHz
190 180 170 160 150 140 130 120 110 100 90 80 70 60 50 40 30 20 10 ppm

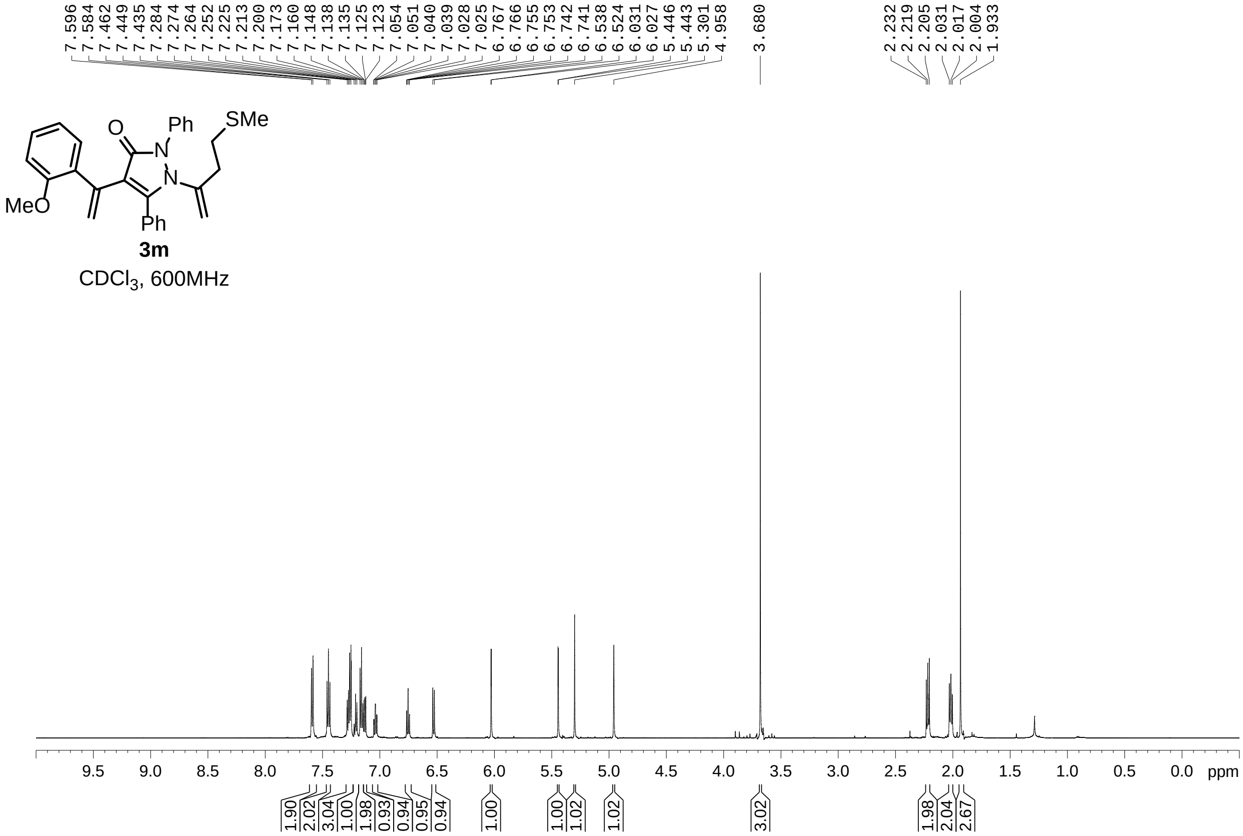

3m
$CDCl_3$, 600MHz

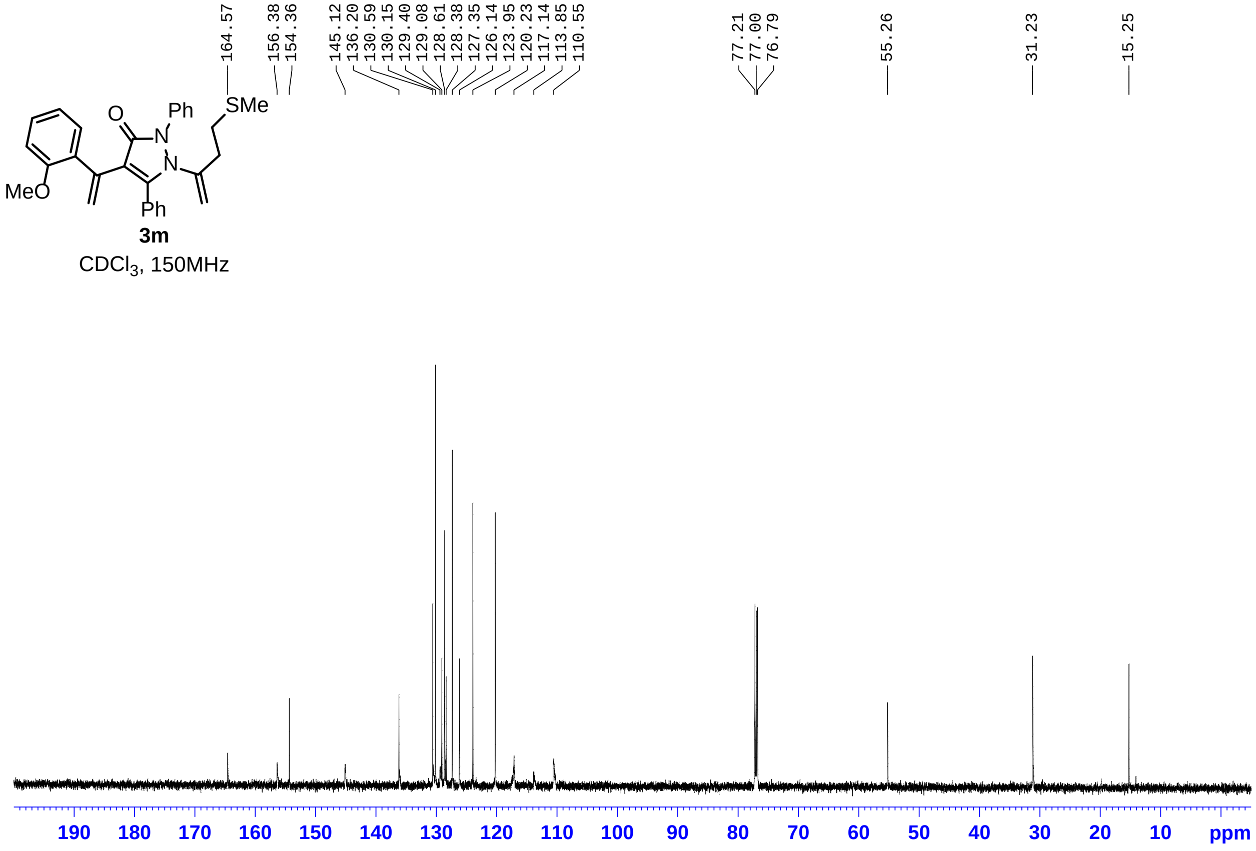

3m
$CDCl_3$, 150MHz

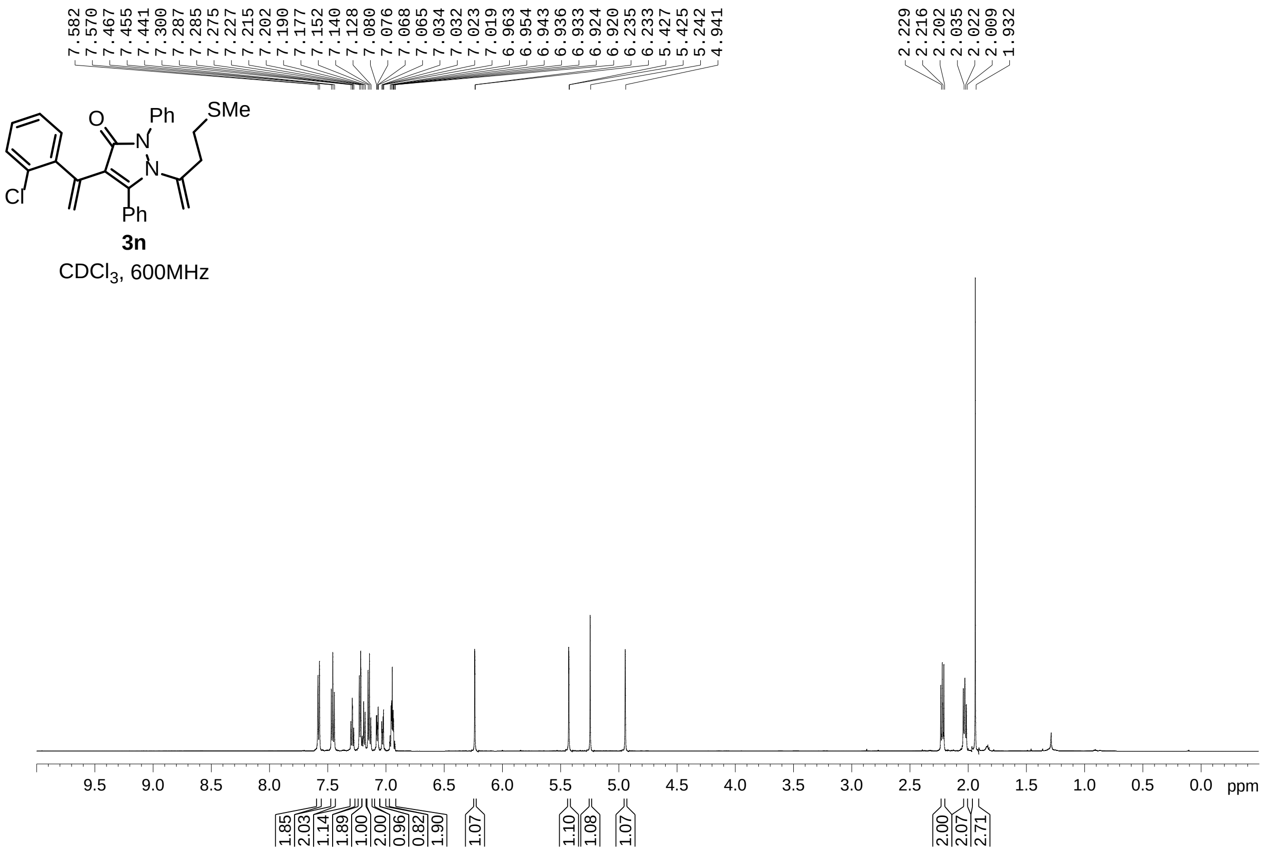
7.582 7.570 7.467 7.455 7.441 7.300 7.287 7.285 7.275 7.227 7.215 7.202 7.190 7.177 7.152 7.140 7.128 7.080 7.076 7.068 7.065 7.034 7.032 7.023 7.019 6.963 6.954 6.943 6.936 6.933 6.924 6.920 6.235 6.233 5.427 5.425 5.242 4.941
2.229 2.216 2.202 2.035 2.022 2.009 1.932
Ph
SMe
O
N
N
Cl
Ph
3n
$CDCl_3$, 600MHz
9.5 9.0 8.5 8.0 7.5 7.0 6.5 6.0 5.5 5.0 4.5 4.0 3.5 3.0 2.5 2.0 1.5 1.0 0.5 0.0 ppm
1.85 2.03 1.14 1.89 1.00 2.00 0.96 0.82 1.90 1.07 1.10 1.08 1.07 2.00 2.07 2.71

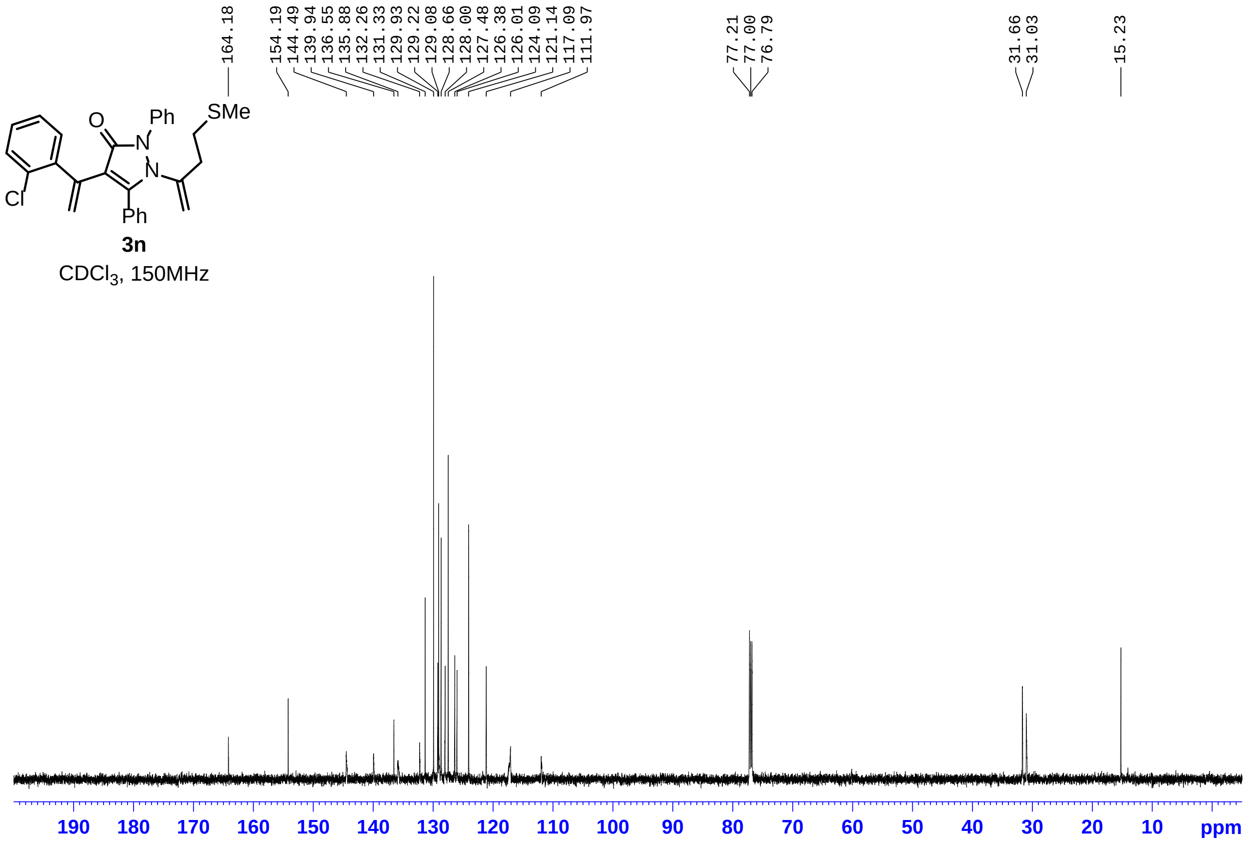
164.18
154.19 144.49 139.94 136.55 135.88 132.26 131.33 129.93 129.22 129.08 128.66 128.00 127.48 126.38 126.01 124.09 121.14 117.09 111.97
77.21 77.00 76.79
31.66 31.03
15.23
Ph
SMe
O
N
N
Cl
Ph
3n
$CDCl_3$, 150MHz
190 180 170 160 150 140 130 120 110 100 90 80 70 60 50 40 30 20 10 ppm

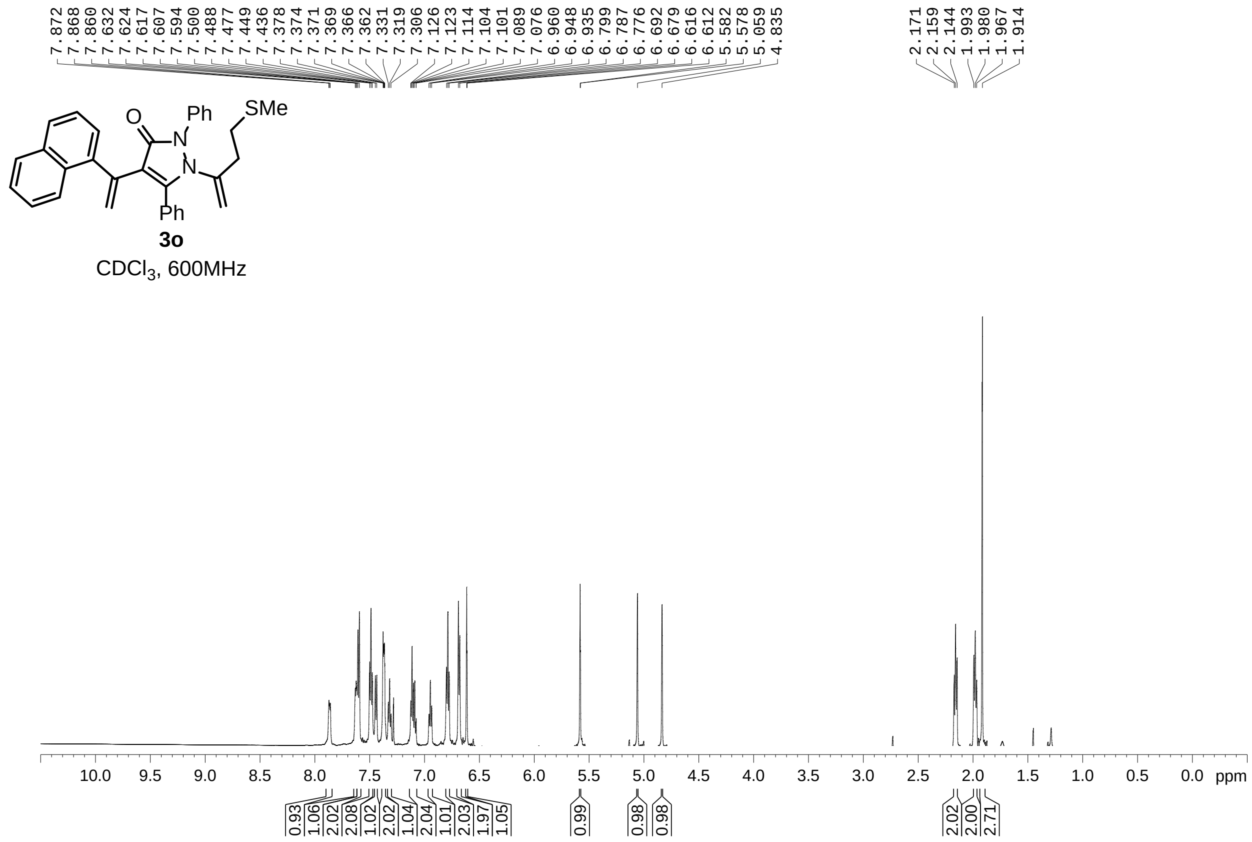
7.872 7.868 7.860 7.632 7.624 7.617 7.607 7.594 7.500 7.488 7.477 7.449 7.436 7.378 7.374 7.371 7.369 7.366 7.362 7.331 7.319 7.306 7.126 7.123 7.114 7.104 7.101 7.089 7.076 6.960 6.948 6.935 6.799 6.787 6.776 6.692 6.679 6.616 6.612 5.582 5.578 5.059 4.835
2.171 2.159 2.144 1.993 1.980 1.967 1.914
O
N
Ph
SMe
N
Ph
3o
CDCl3, 600MHz
10.0 9.5 9.0 8.5 8.0 7.5 7.0 6.5 6.0 5.5 5.0 4.5 4.0 3.5 3.0 2.5 2.0 1.5 1.0 0.5 0.0 ppm
0.93 1.06 2.02 2.08 1.02 2.02 1.04 2.04 1.01 2.03 1.97 1.05
0.99
0.98
0.98
2.02 2.00 2.71

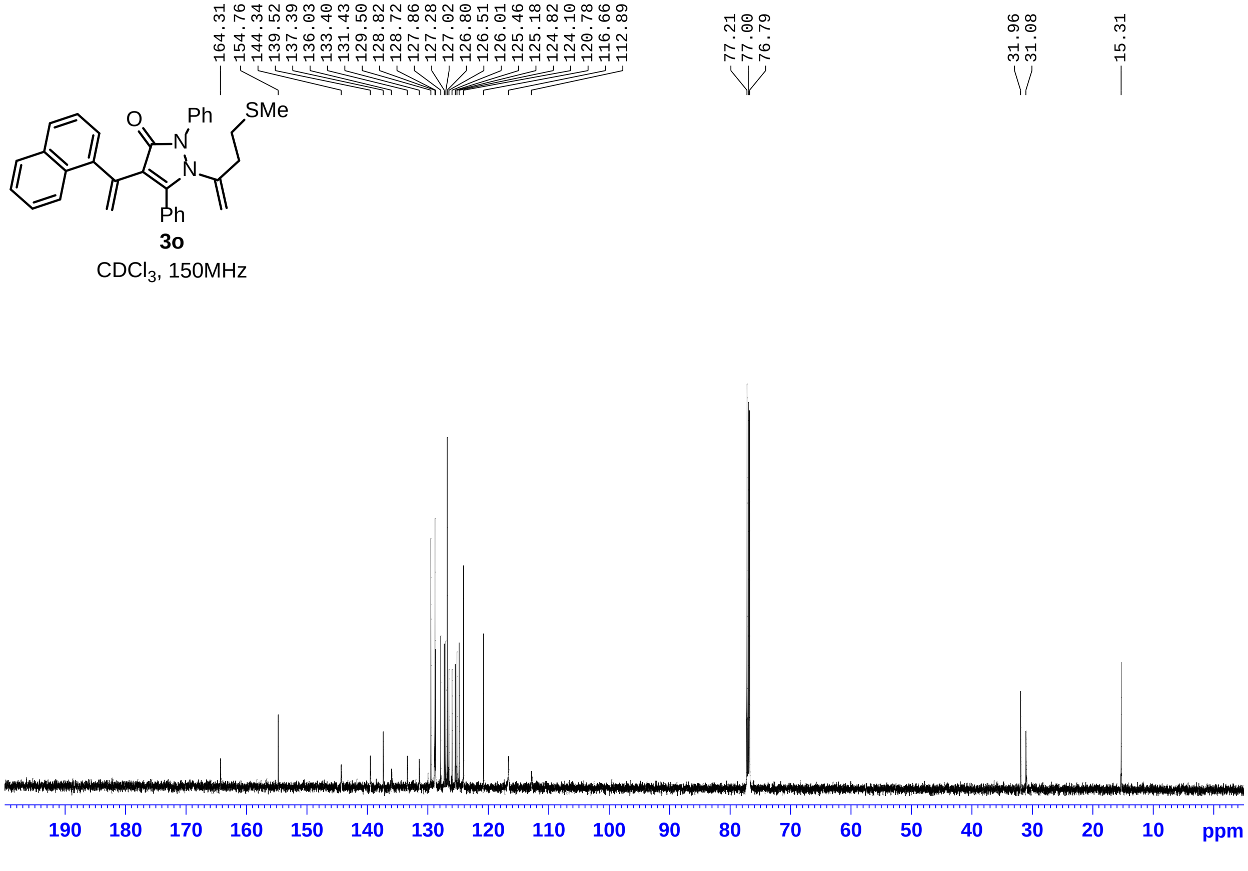
164.31 154.76 144.34 139.52 137.39 136.03 133.40 131.43 129.50 128.82 128.72 127.86 127.28 127.02 126.80 126.51 126.01 125.46 125.18 124.82 124.10 120.78 116.66 112.89
77.21 77.00 76.79
31.96 31.08
15.31
O
N
Ph
SMe
N
Ph
3o
CDCl3, 150MHz
190 180 170 160 150 140 130 120 110 100 90 80 70 60 50 40 30 20 10 ppm

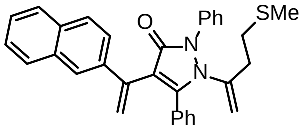

3p

$CDCl_3$, 600MHz

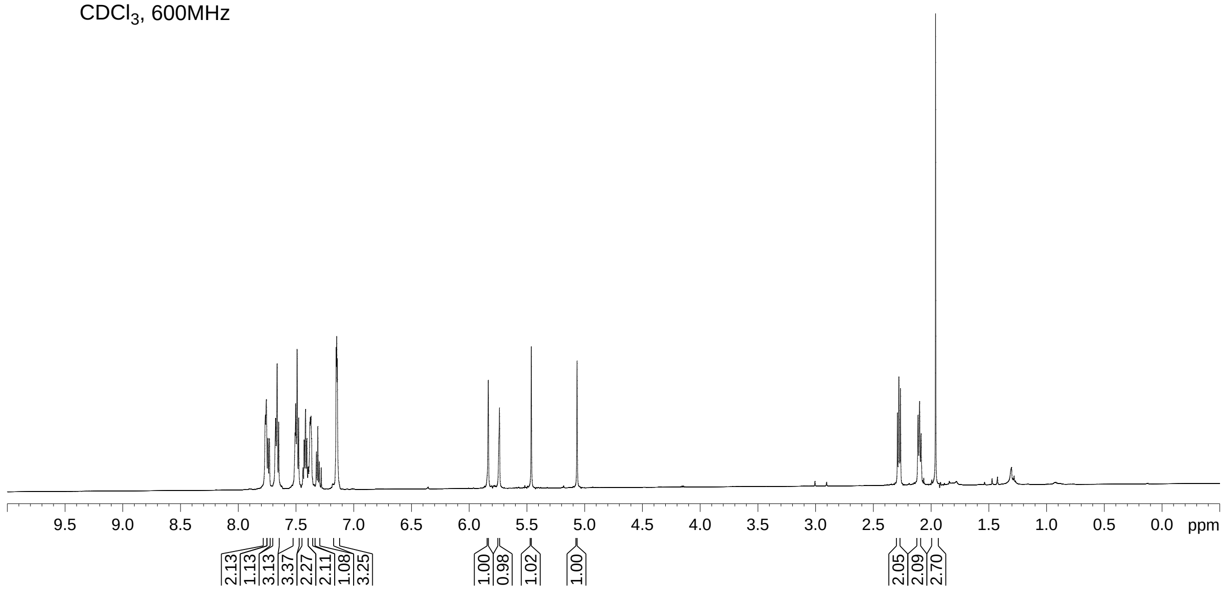

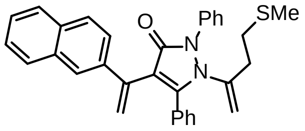

3p

$CDCl_3$, 150MHz

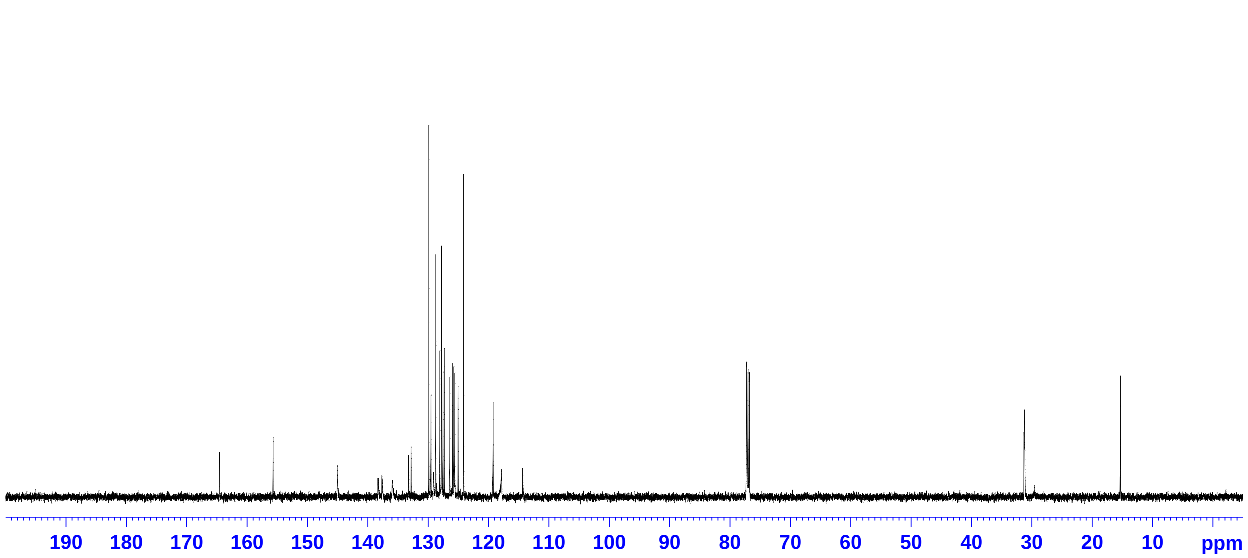

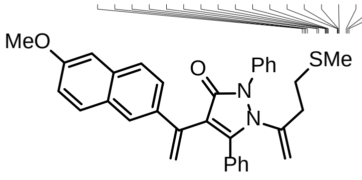

3q

CDCl$_3$, 600MHz

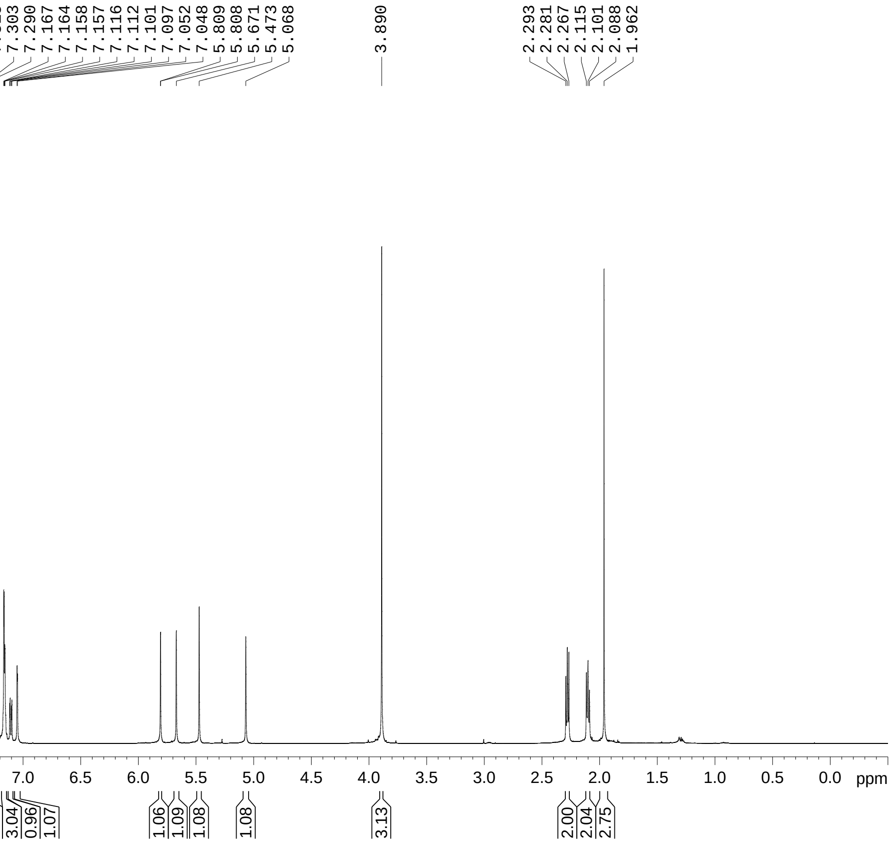

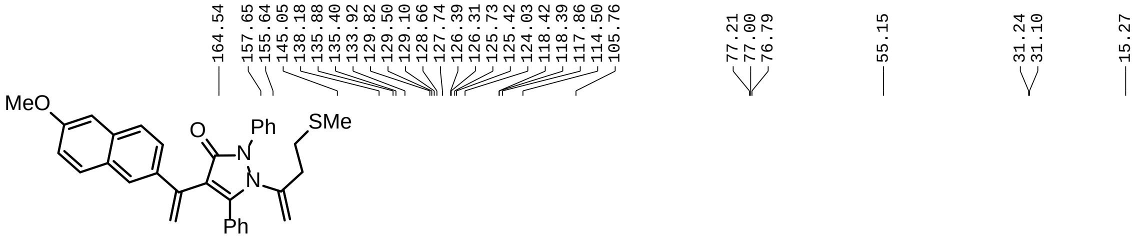

3q

CDCl$_3$, 150MHz

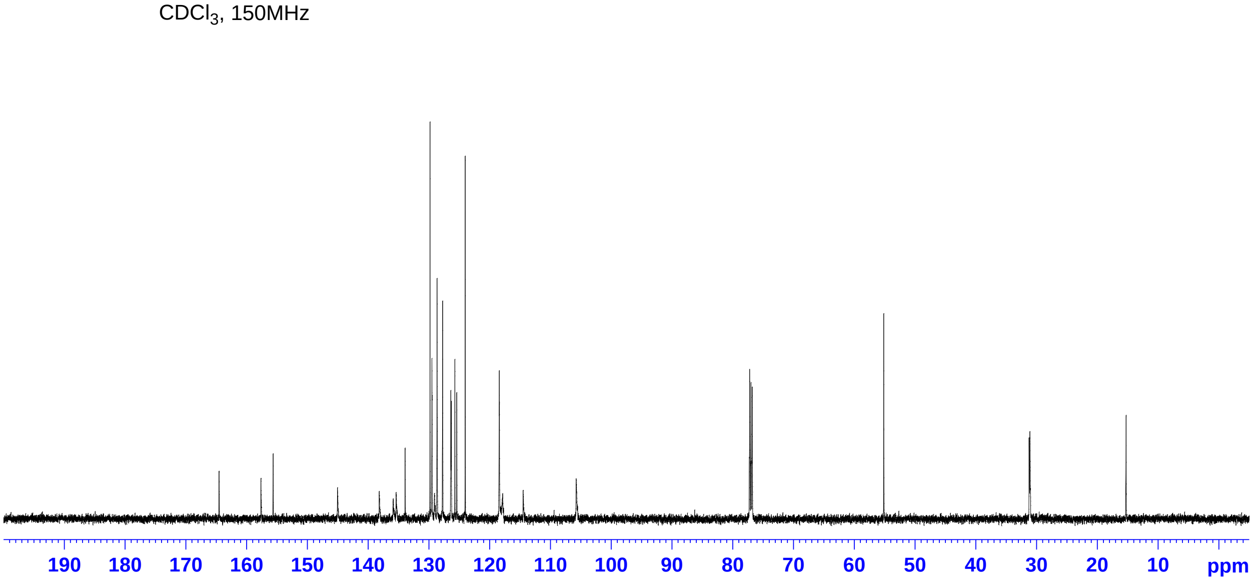

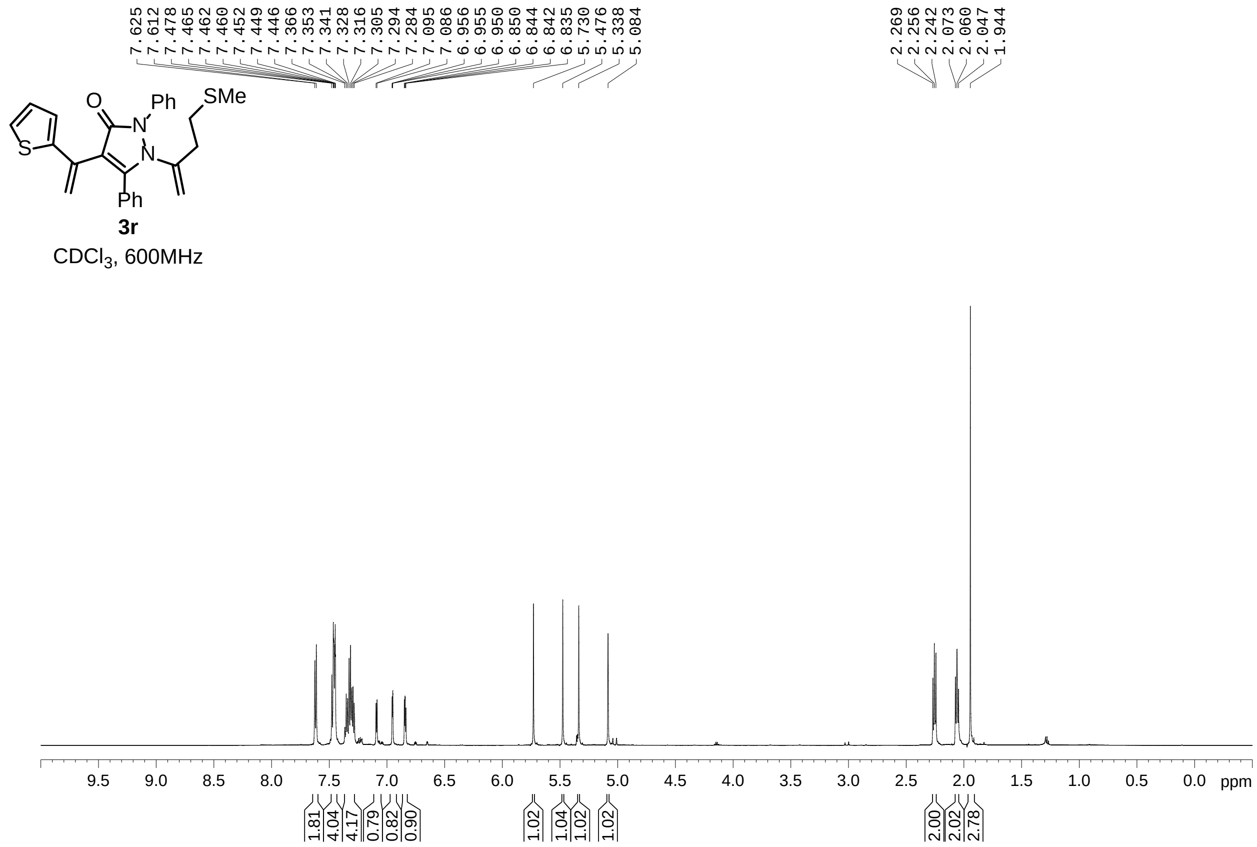
7.625
7.612
7.478
7.465
7.462
7.460
7.452
7.449
7.446
7.366
7.353
7.341
7.328
7.316
7.305
7.294
7.284
7.095
7.086
6.956
6.955
6.950
6.850
6.844
6.842
6.835
5.730
5.476
5.338
5.084
2.269
2.256
2.242
2.073
2.060
2.047
1.944
O
Ph
N
N
SMe
S
Ph
3r
CDCl3, 600MHz
9.5 9.0 8.5 8.0 7.5 7.0 6.5 6.0 5.5 5.0 4.5 4.0 3.5 3.0 2.5 2.0 1.5 1.0 0.5 0.0 ppm
1.81
4.04
4.17
0.79
0.82
0.90
1.02
1.04
1.02
1.02
2.00
2.02
2.78

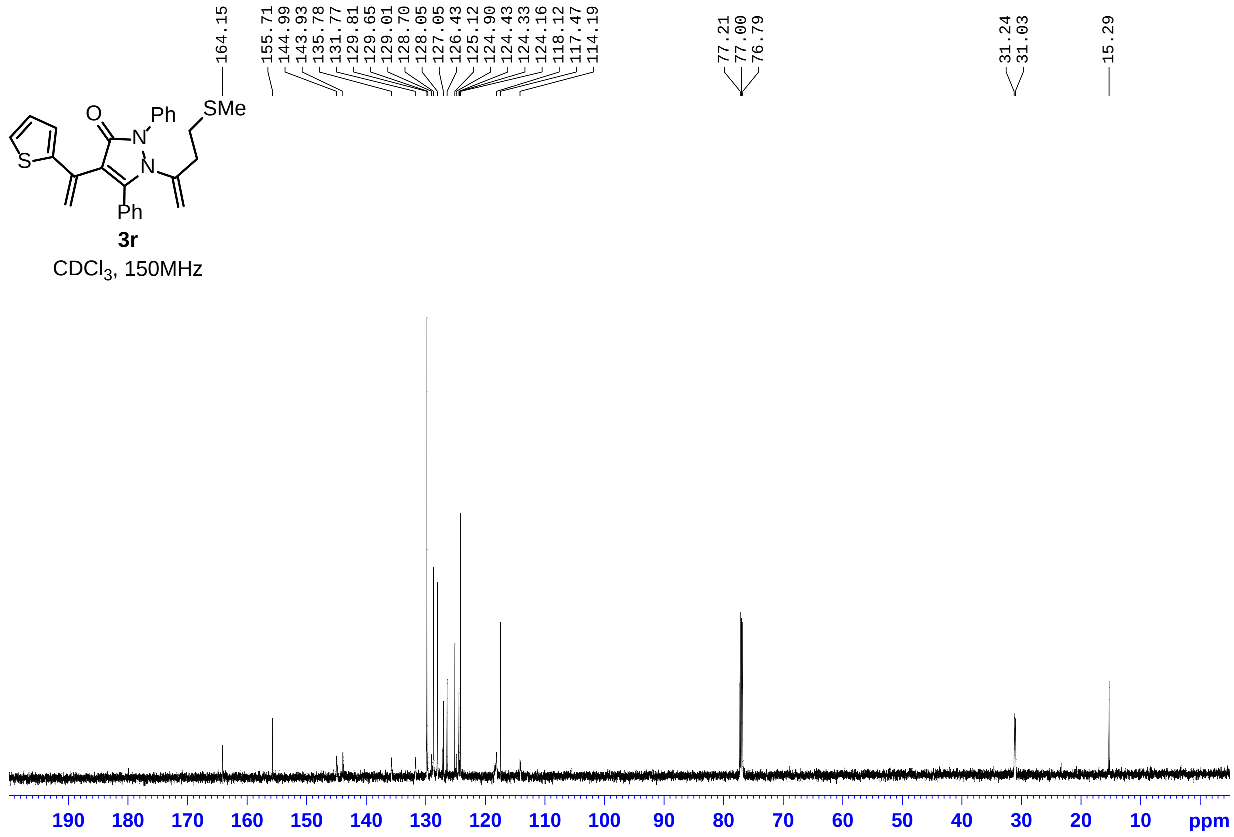
164.15
155.71
144.99
143.93
135.78
131.77
129.81
129.65
129.01
128.70
128.05
127.05
126.43
125.12
124.90
124.43
124.33
124.16
118.12
117.47
114.19
77.21
77.00
76.79
31.24
31.03
15.29
O
Ph
N
N
SMe
S
Ph
3r
CDCl3, 150MHz
190 180 170 160 150 140 130 120 110 100 90 80 70 60 50 40 30 20 10 ppm

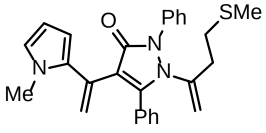

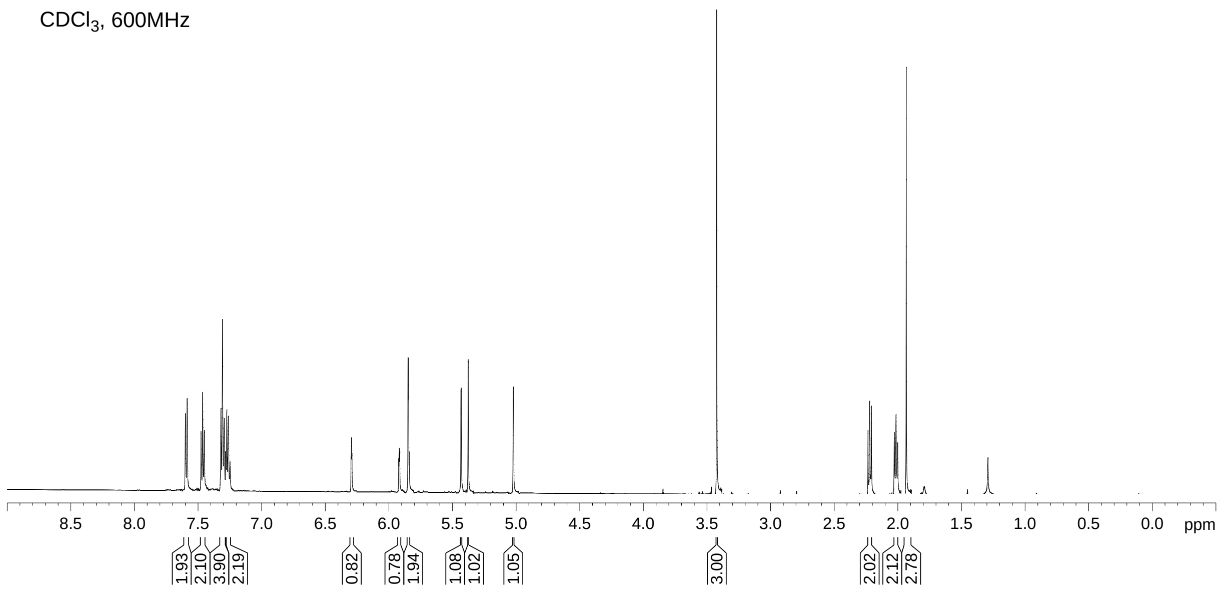

3s

$CDCl_3$, 600MHz

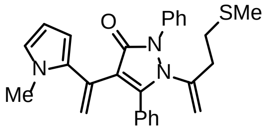

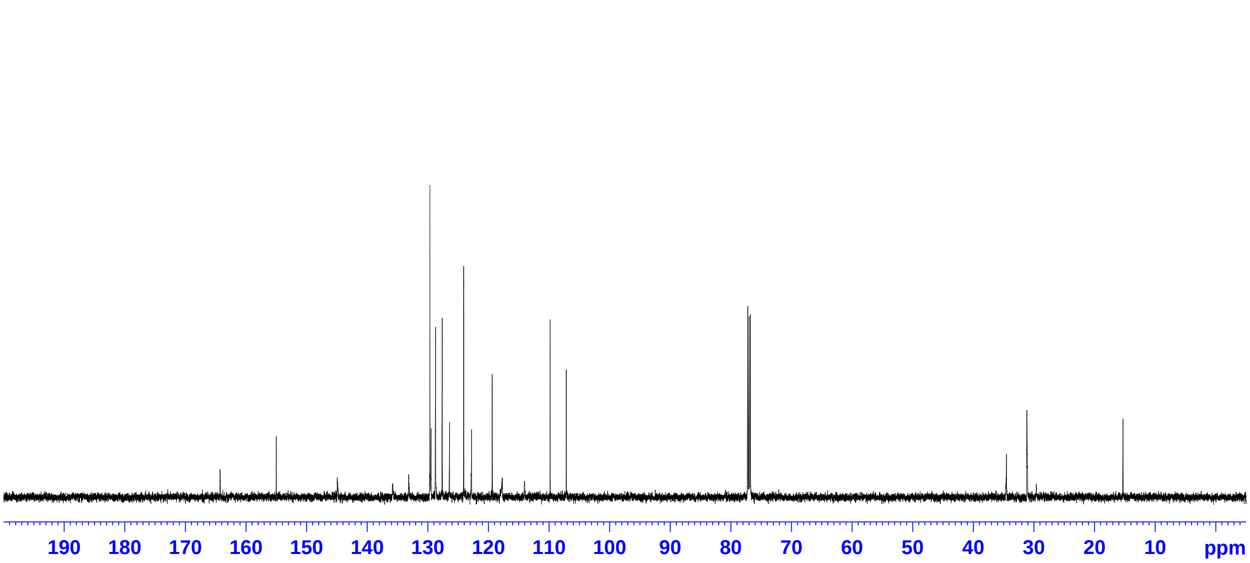

3s

$CDCl_3$, 150MHz

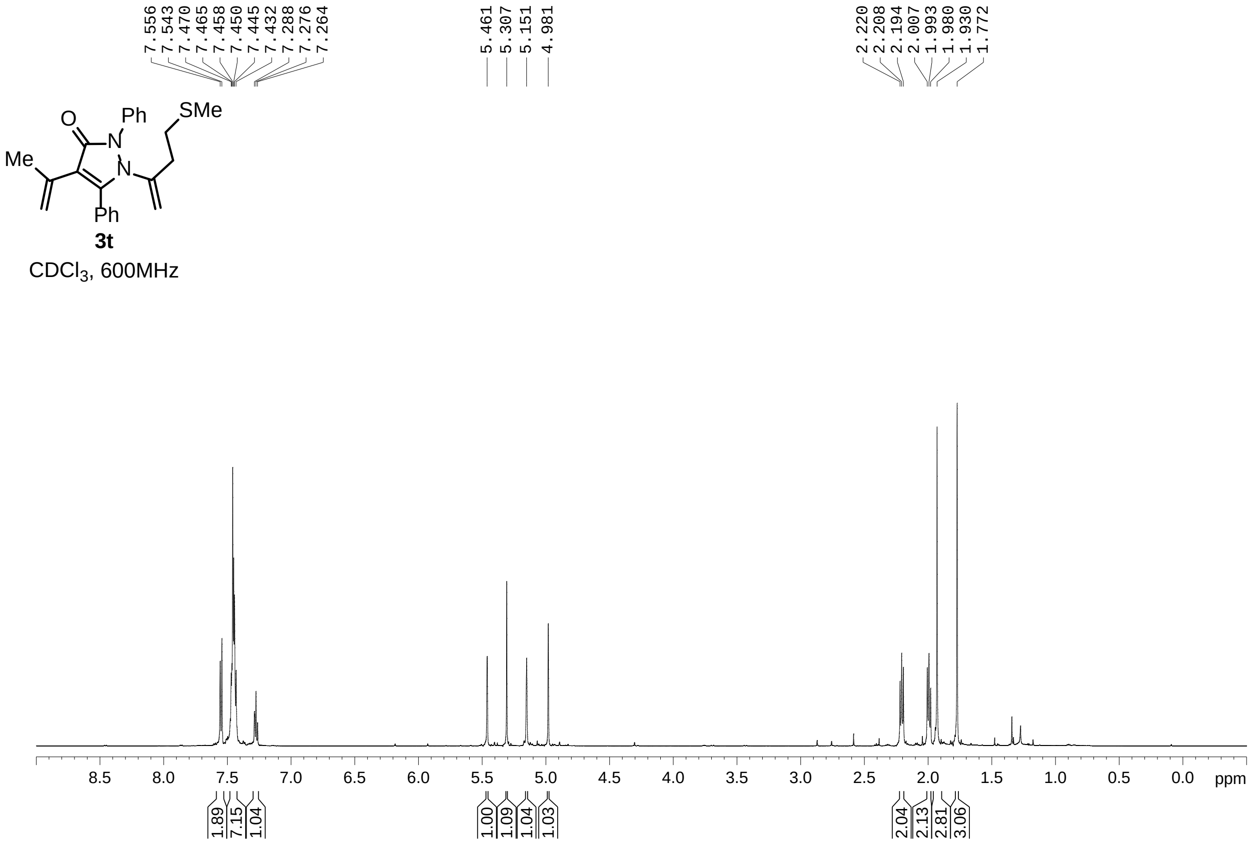
7.556 7.543 7.470 7.465 7.458 7.450 7.445 7.432 7.288 7.276 7.264
5.461 5.307 5.151 4.981
2.220 2.208 2.194 2.007 1.993 1.980 1.930 1.772
O
Ph
SMe
N
N
Me
Ph
3t
CDCl3, 600MHz
8.5 8.0 7.5 7.0 6.5 6.0 5.5 5.0 4.5 4.0 3.5 3.0 2.5 2.0 1.5 1.0 0.5 0.0 ppm
1.89 7.15 1.04 1.00 1.09 1.04 1.03 2.04 2.13 2.81 3.06

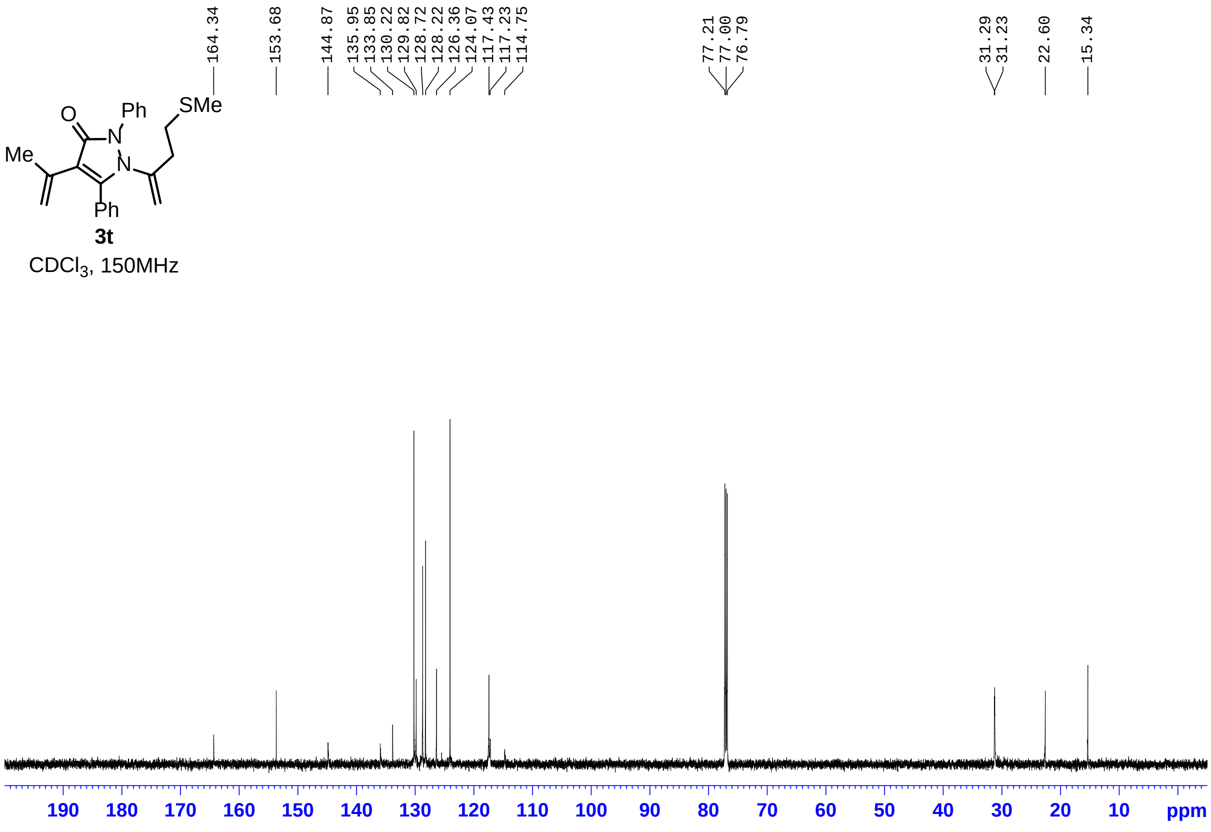
164.34
153.68
144.87
135.95 133.85 130.22 129.82 128.72 128.22 126.36 124.07 117.43 117.23 114.75
77.21 77.00 76.79
31.29 31.23
22.60
15.34
O
Ph
SMe
N
N
Me
Ph
3t
CDCl3, 150MHz
190 180 170 160 150 140 130 120 110 100 90 80 70 60 50 40 30 20 10 ppm

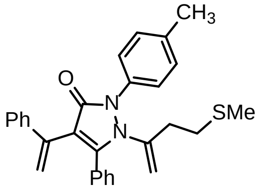

3aa

$CDCl_3$, 600MHz

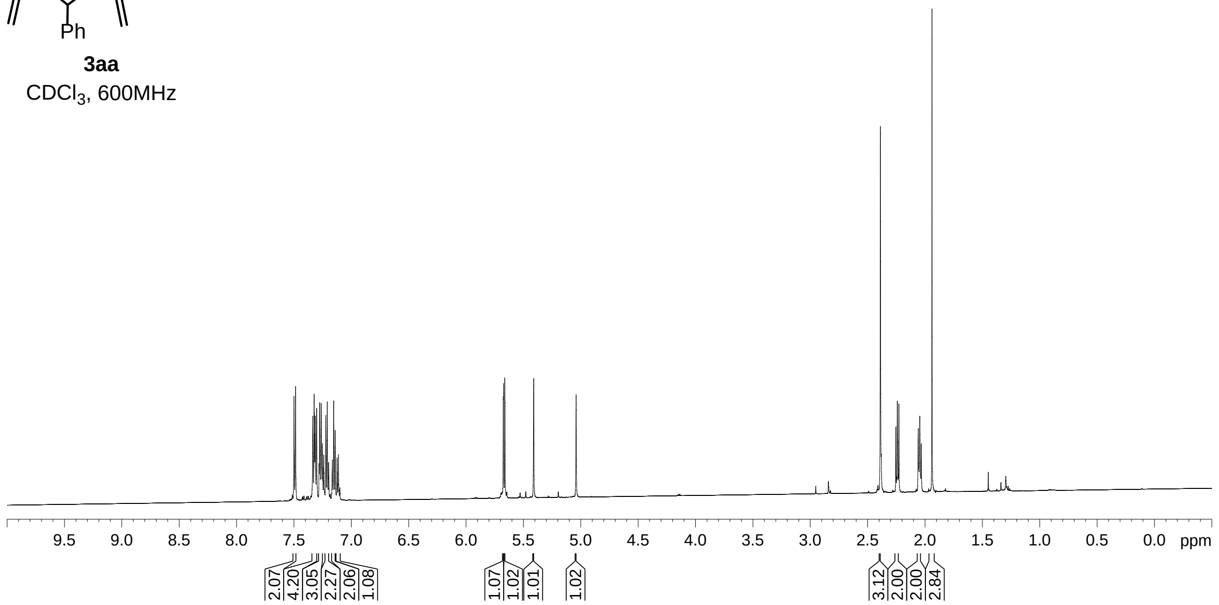

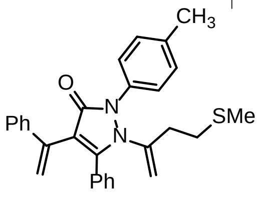

3aa

$CDCl_3$, 150MHz

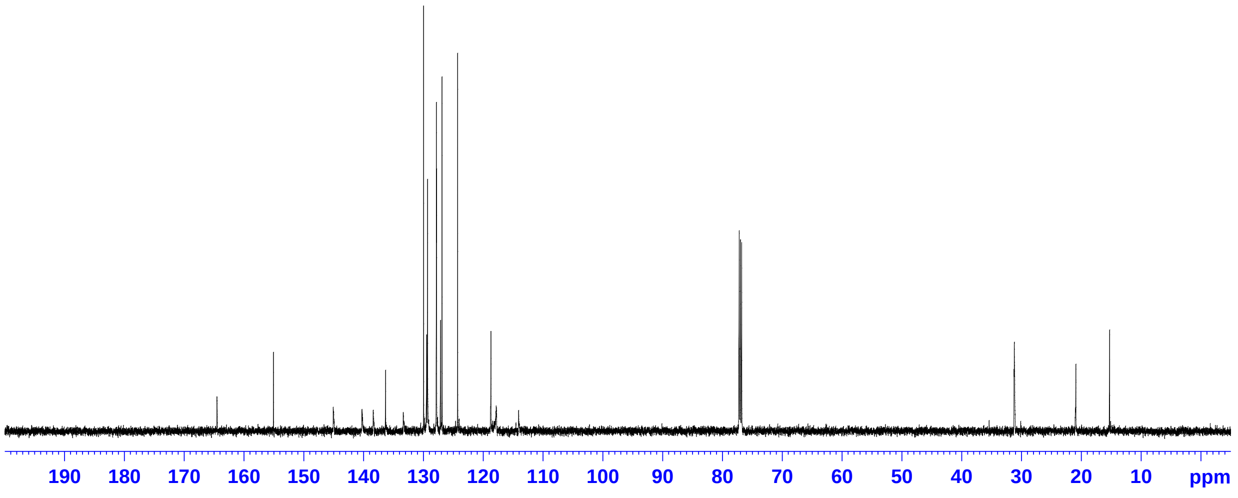

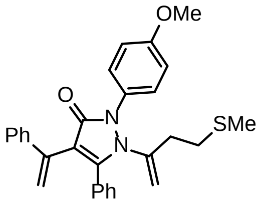

3ab

$CDCl_3$, 600MHz

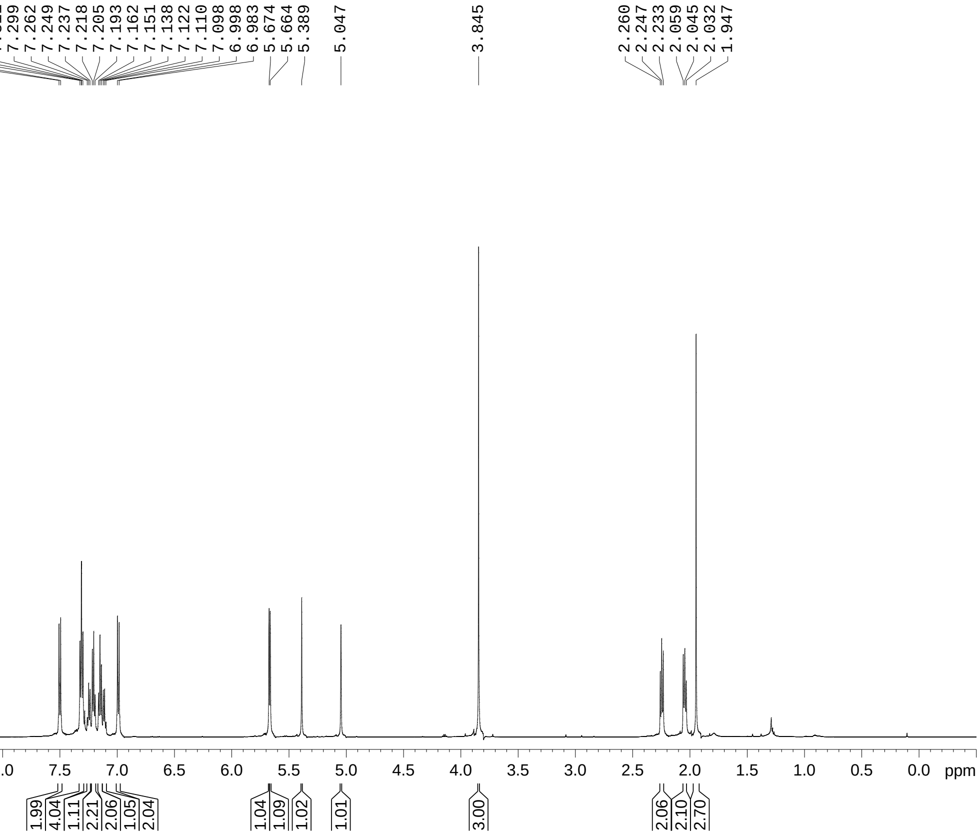

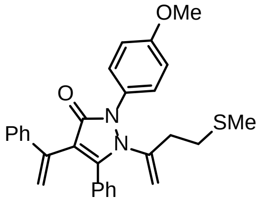

3ab

$CDCl_3$, 150MHz

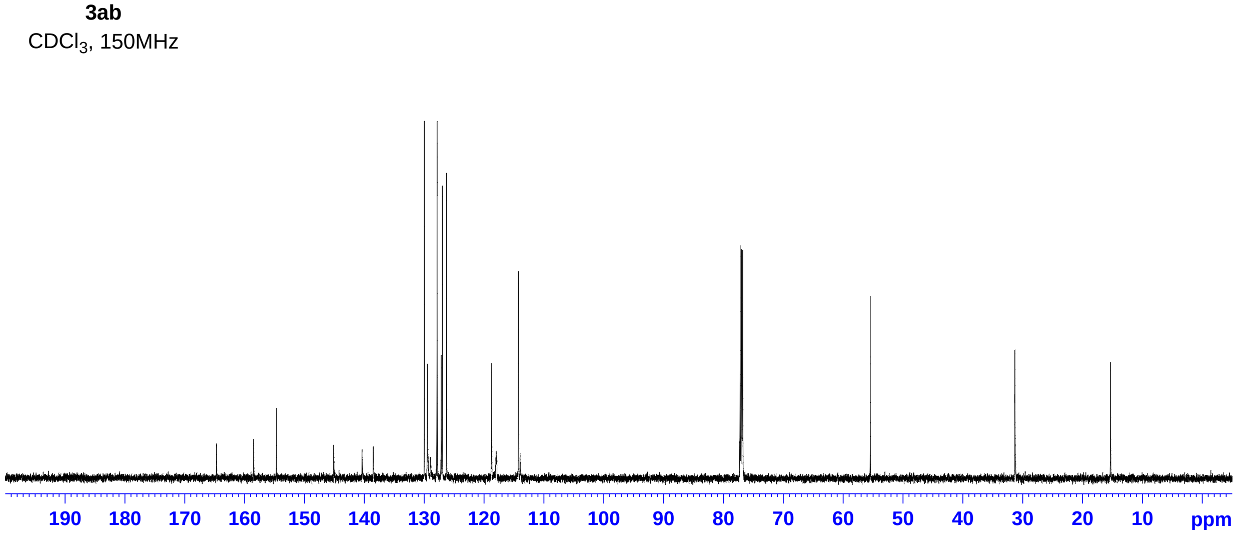

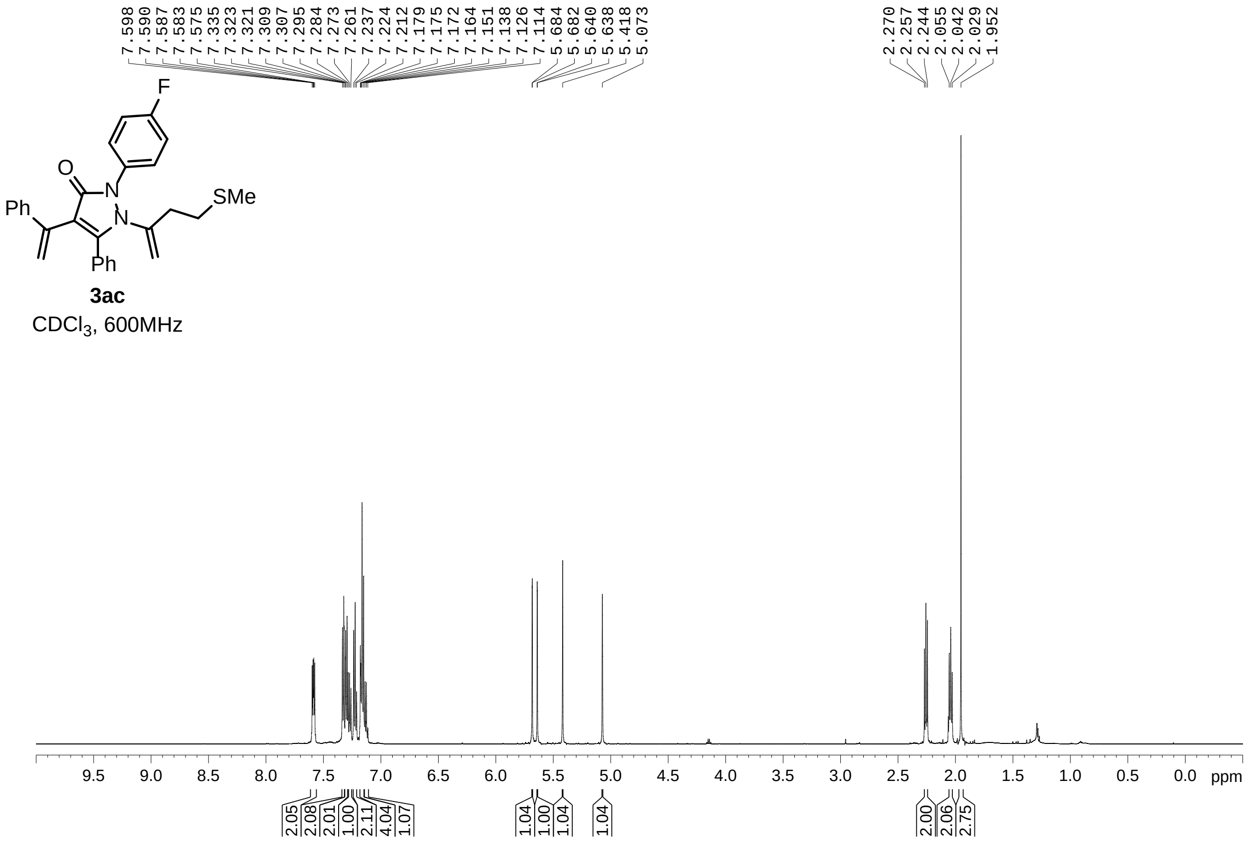
7.598 7.590 7.587 7.583 7.575 7.335 7.323 7.321 7.309 7.307 7.295 7.284 7.273 7.261 7.237 7.224 7.212 7.179 7.175 7.172 7.164 7.151 7.138 7.126 7.114 5.684 5.682 5.640 5.638 5.418 5.073
2.270 2.257 2.244 2.055 2.042 2.029 1.952
F
O
N
N
Ph
SMe
Ph
3ac
CDCl3, 600MHz
9.5 9.0 8.5 8.0 7.5 7.0 6.5 6.0 5.5 5.0 4.5 4.0 3.5 3.0 2.5 2.0 1.5 1.0 0.5 0.0 ppm
2.05 2.08 2.01 1.00 2.11 4.04 1.07
1.04 1.00 1.04
1.04
2.00 2.06 2.75

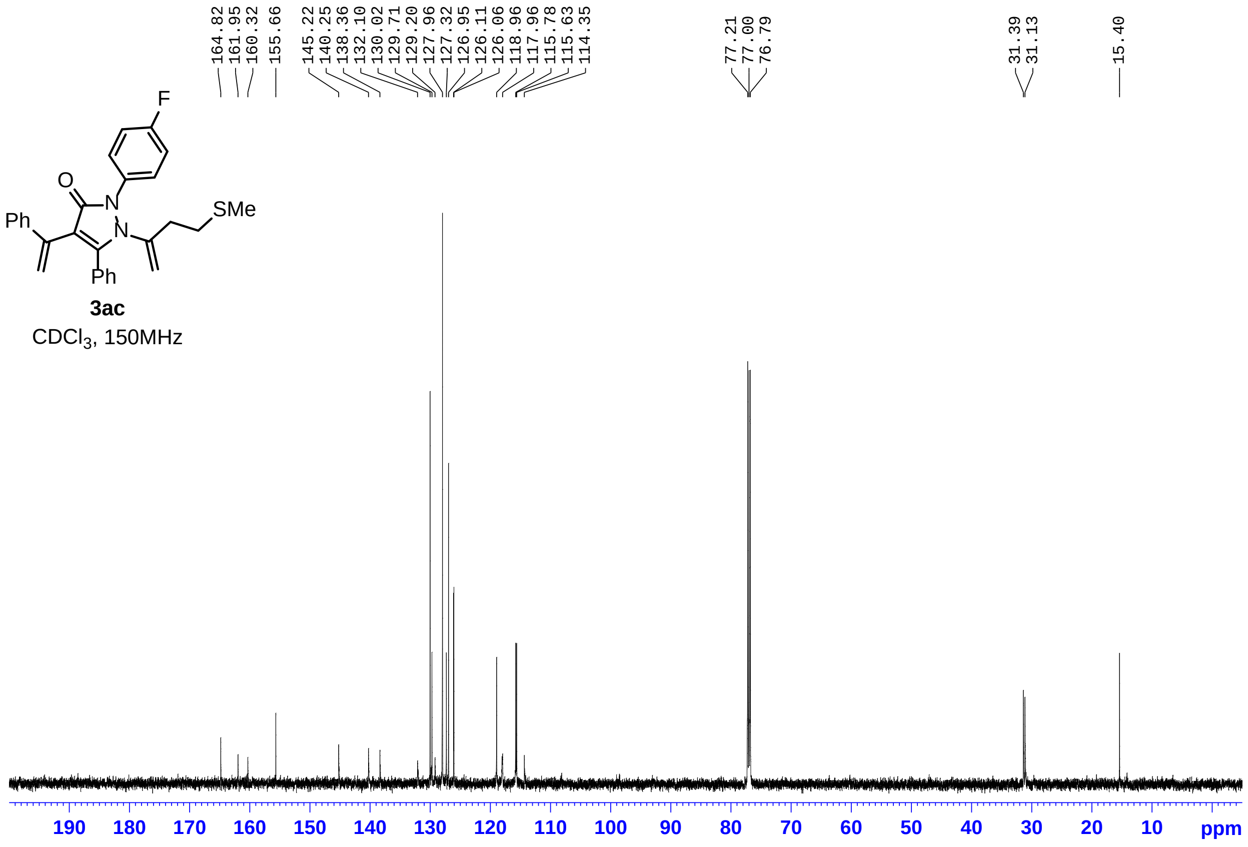
164.82 161.95 160.32 155.66
145.22 140.25 138.36 132.10 130.02 129.71 129.20 127.96 127.32 126.95 126.11 126.06 118.96 117.96 115.78 115.63 114.35
77.21 77.00 76.79
31.39 31.13
15.40
F
O
N
N
Ph
SMe
Ph
3ac
CDCl3, 150MHz
190 180 170 160 150 140 130 120 110 100 90 80 70 60 50 40 30 20 10 ppm

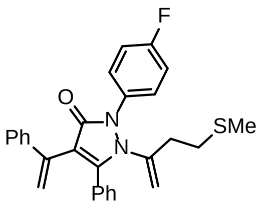

3ac

$CDCl_3$, 600MHz

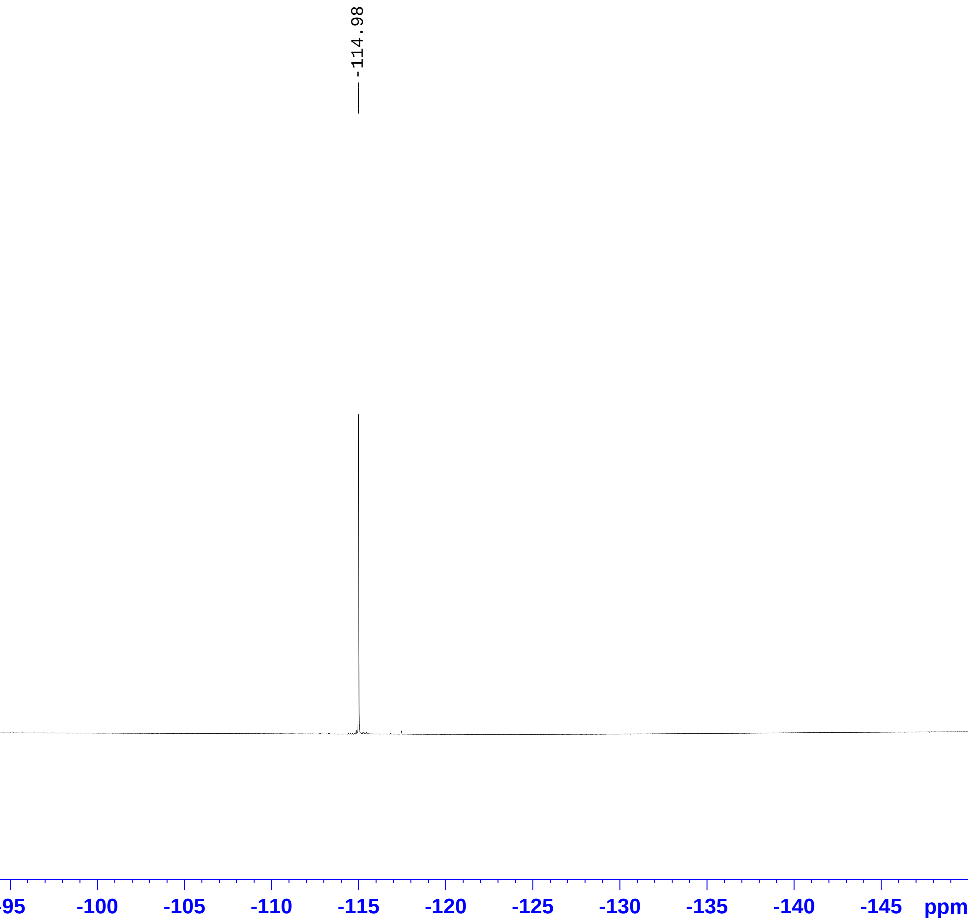

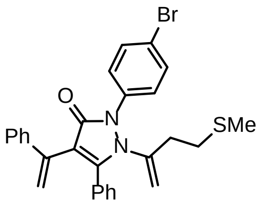

3ad

$CDCl_3$, 600MHz

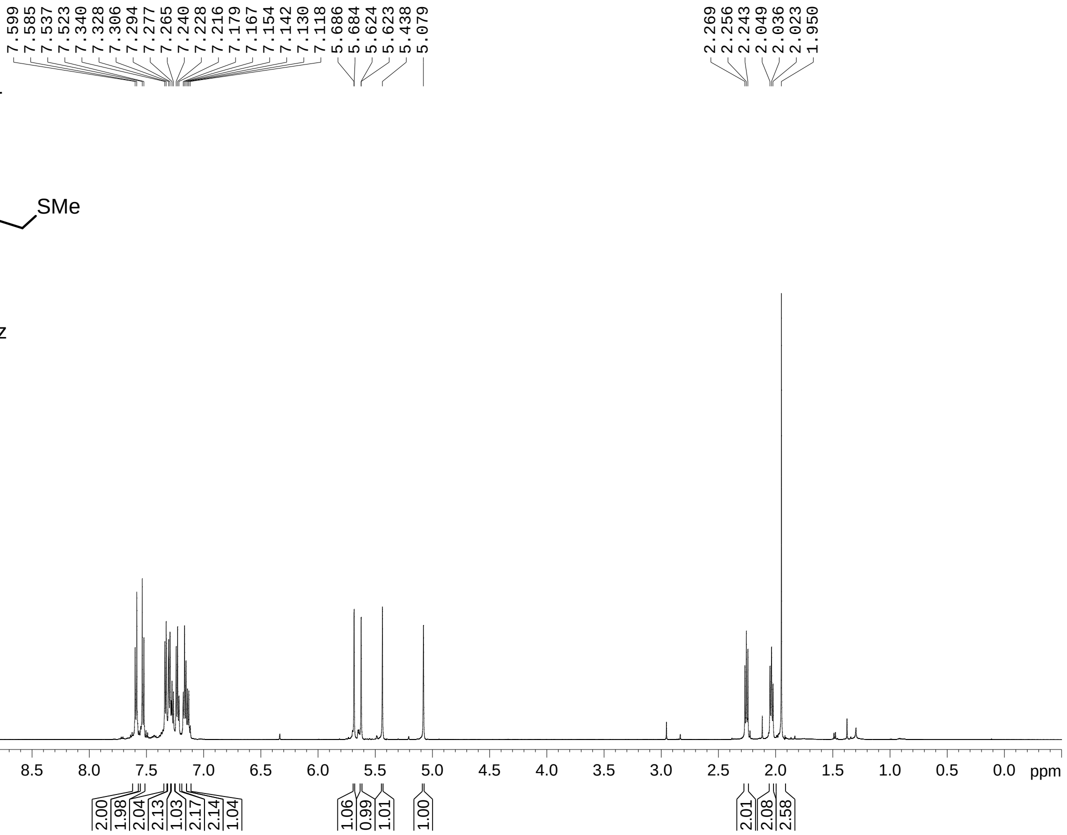

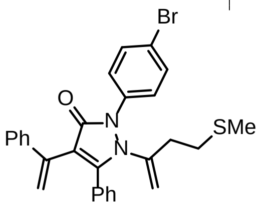

3ad

$CDCl_3$, 150MHz

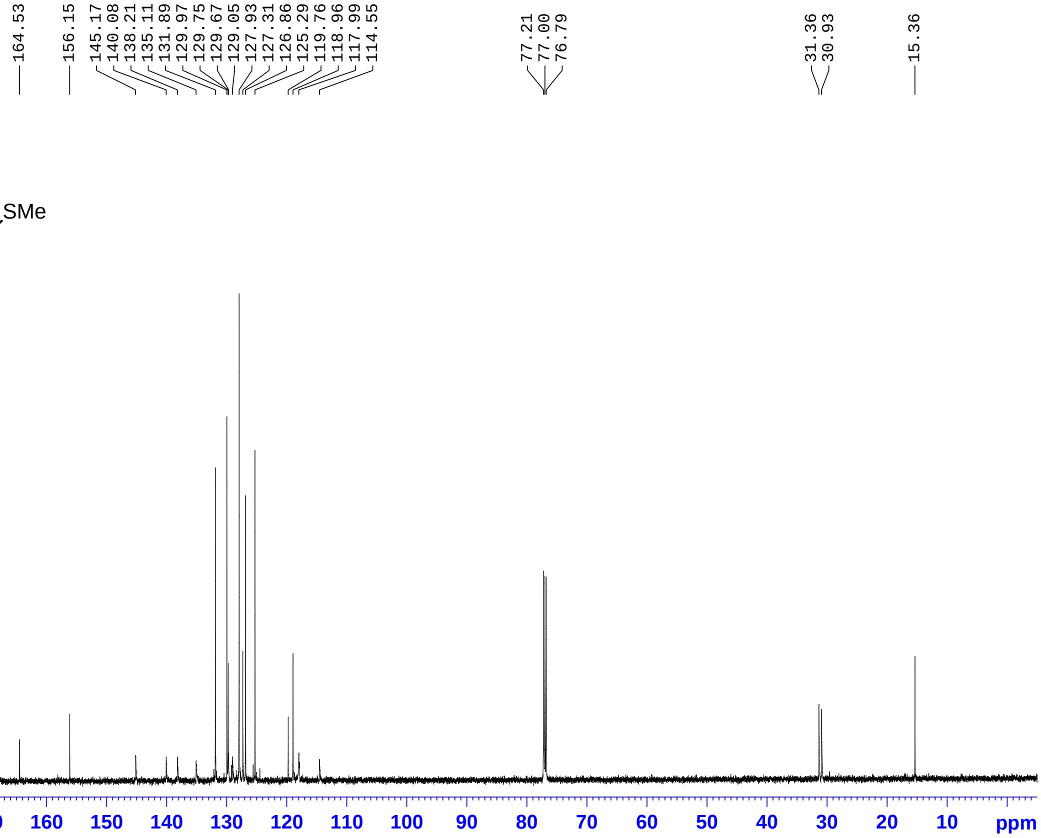

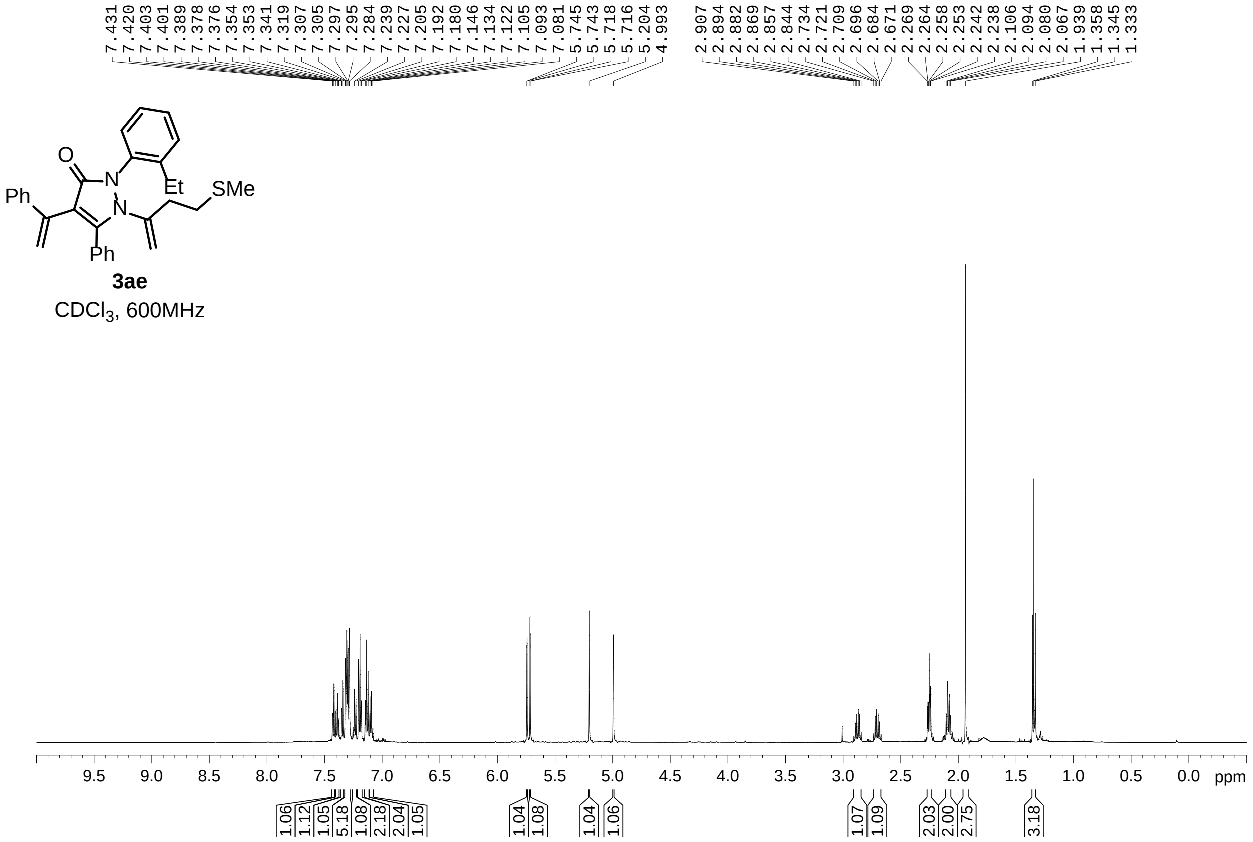

3ae

$CDCl_3$, 600MHz

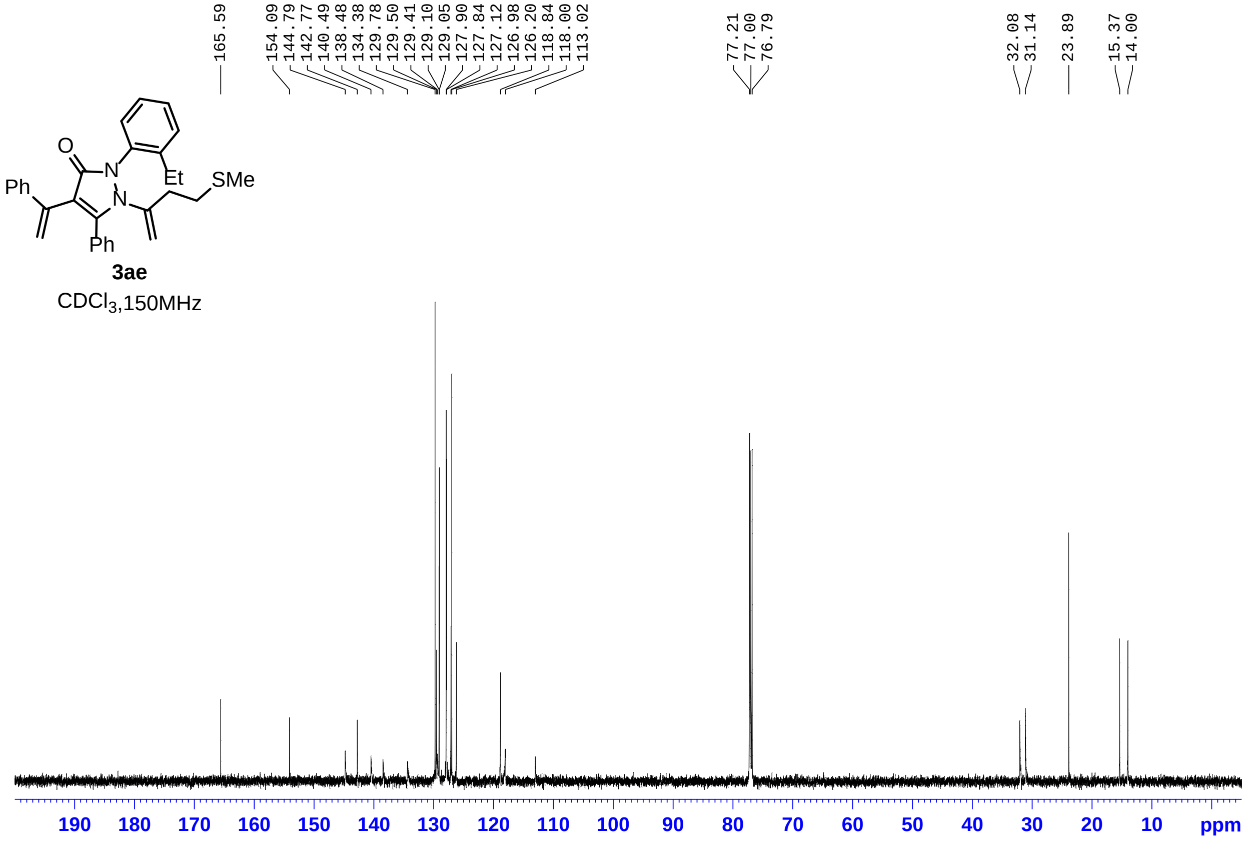

3ae

$CDCl_3$,150MHz

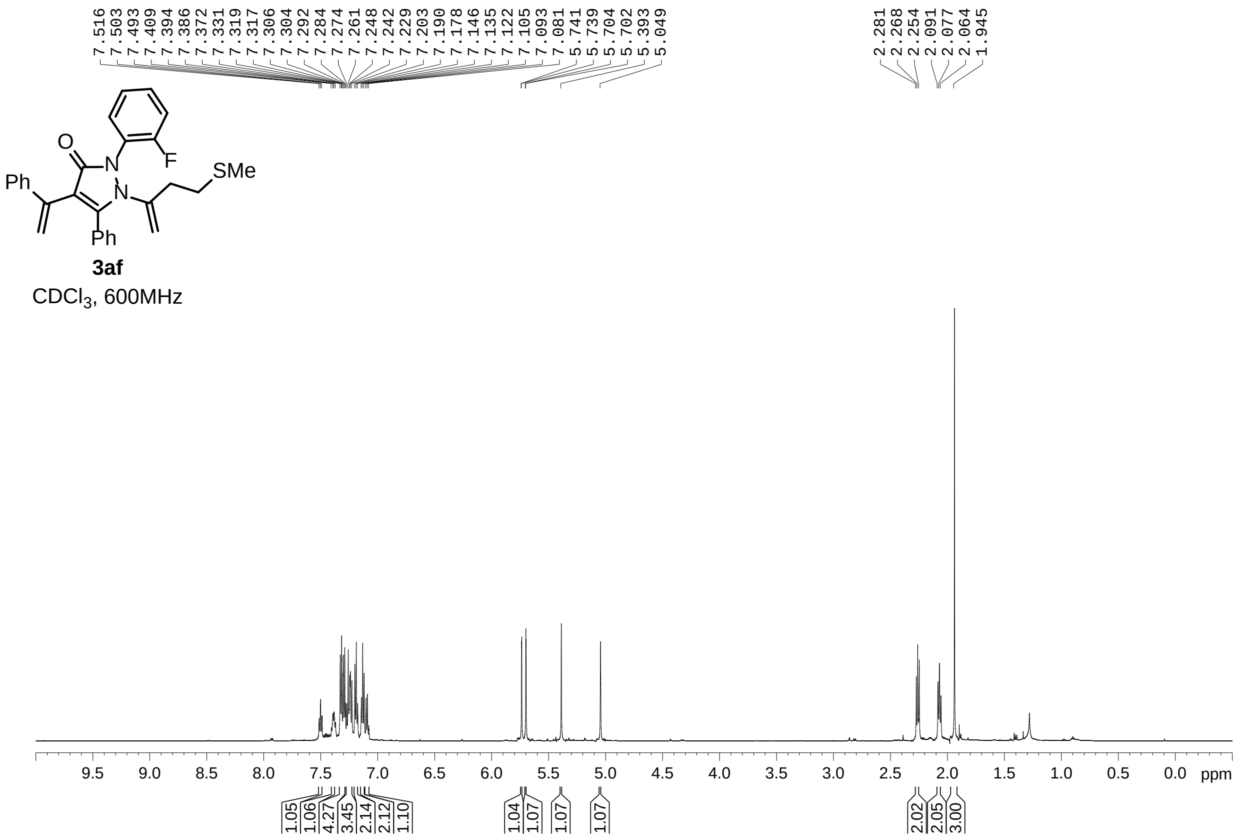
7.516 7.503 7.493 7.409 7.394 7.386 7.372 7.331 7.319 7.317 7.306 7.304 7.292 7.284 7.274 7.261 7.248 7.242 7.229 7.203 7.190 7.178 7.146 7.135 7.122 7.105 7.093 7.081 5.741 5.739 5.704 5.702 5.393 5.049
2.281 2.268 2.254 2.091 2.077 2.064 1.945
O
N
F
SMe
Ph
N
Ph
3af
CDCl3, 600MHz
9.5 9.0 8.5 8.0 7.5 7.0 6.5 6.0 5.5 5.0 4.5 4.0 3.5 3.0 2.5 2.0 1.5 1.0 0.5 0.0 ppm
1.05 1.06 4.27 3.45 2.14 2.12 1.10 1.04 1.07 1.07 1.07 2.02 2.05 3.00

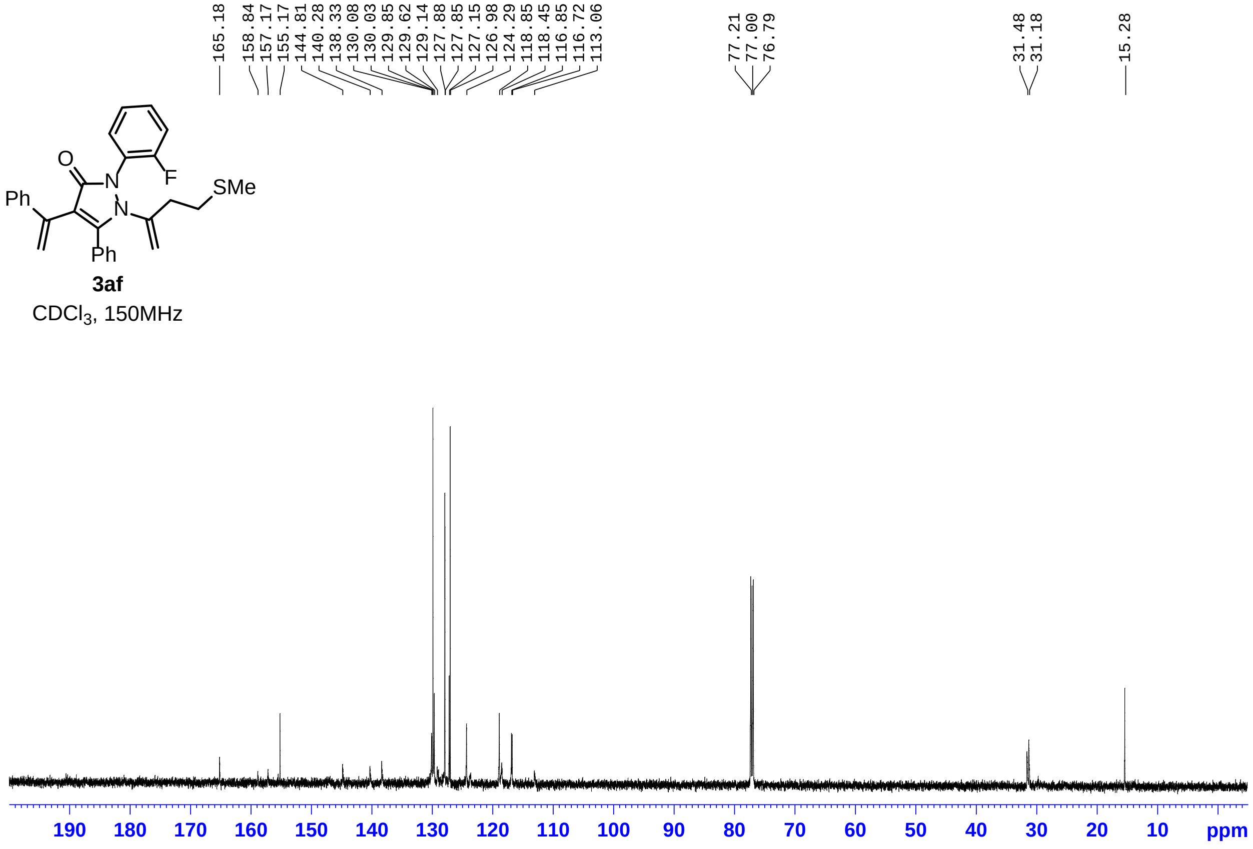
165.18 158.84 157.17 155.17 144.81 140.28 138.33 130.08 130.03 129.85 129.62 129.14 127.88 127.85 127.15 126.98 124.29 118.85 118.45 116.85 116.72 113.06
77.21 77.00 76.79
31.48 31.18
15.28
O
N
F
SMe
Ph
N
Ph
3af
CDCl3, 150MHz
190 180 170 160 150 140 130 120 110 100 90 80 70 60 50 40 30 20 10 ppm

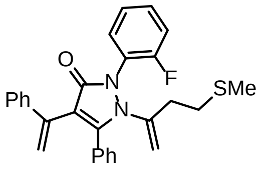

3af

$CDCl_3$, 600MHz

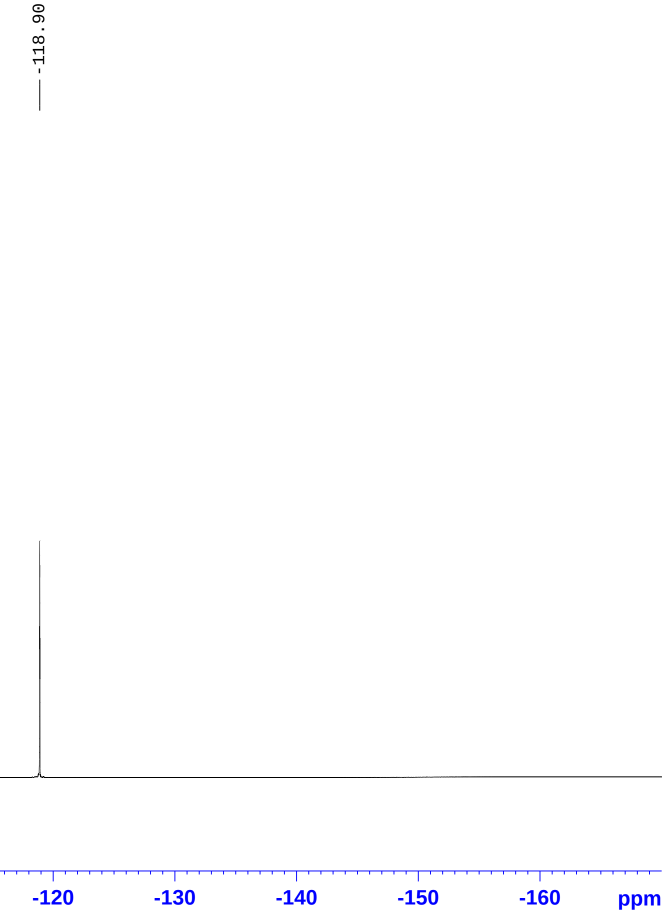

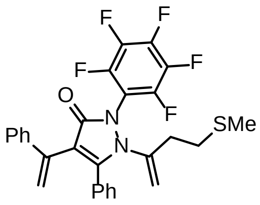

3ag

$CDCl_3$, 600MHz

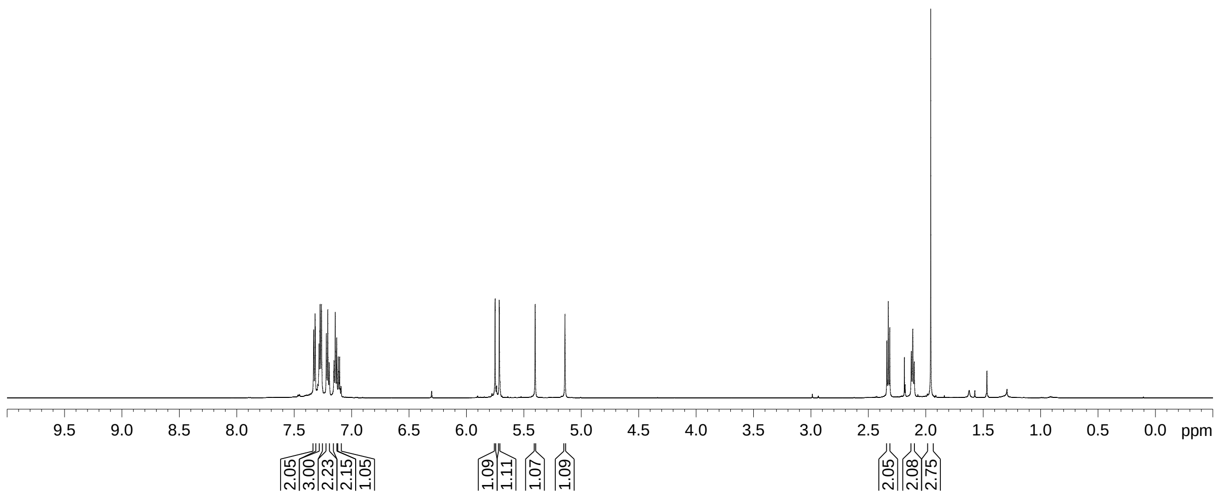

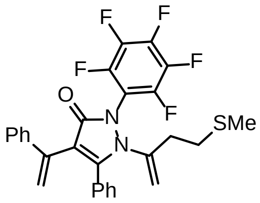

3ag

$CDCl_3$, 150MHz

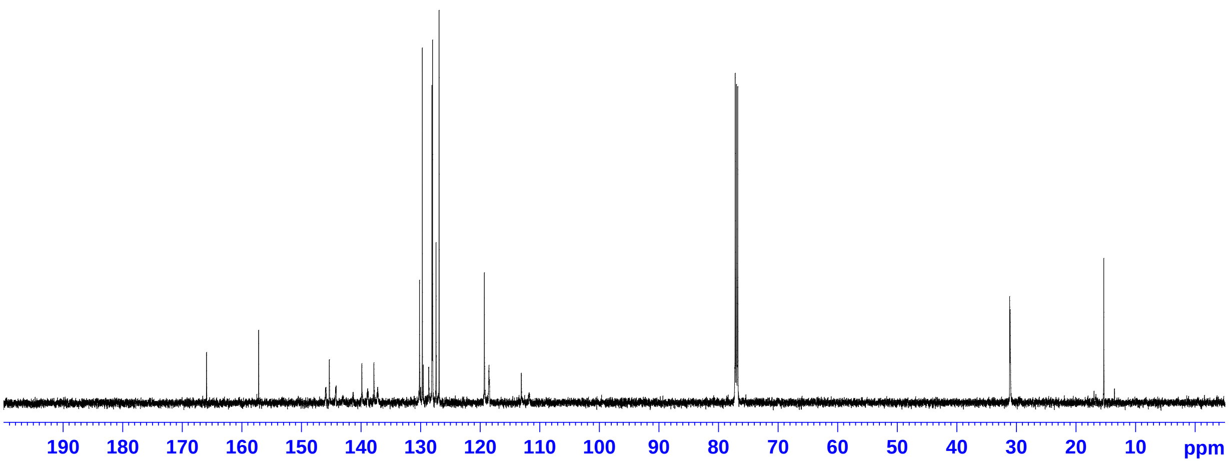

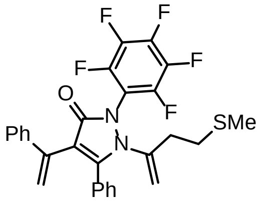

3ag

$CDCl_3$, 600MHz

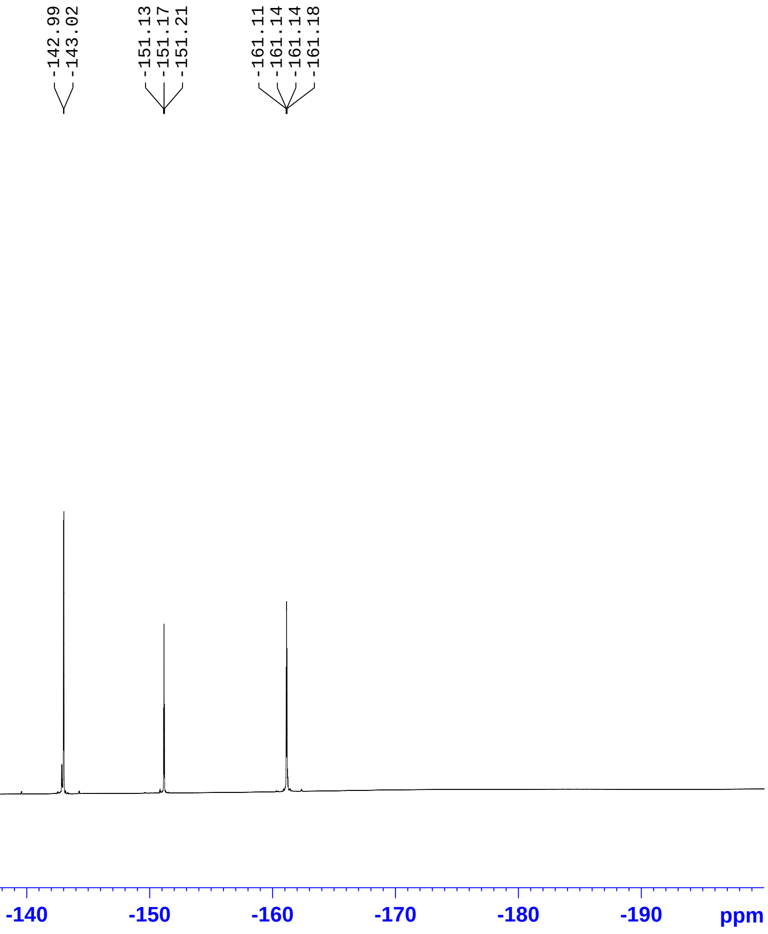

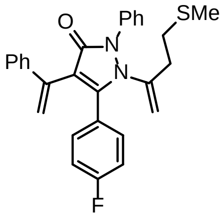

3ah

$CDCl_3$, 600MHz

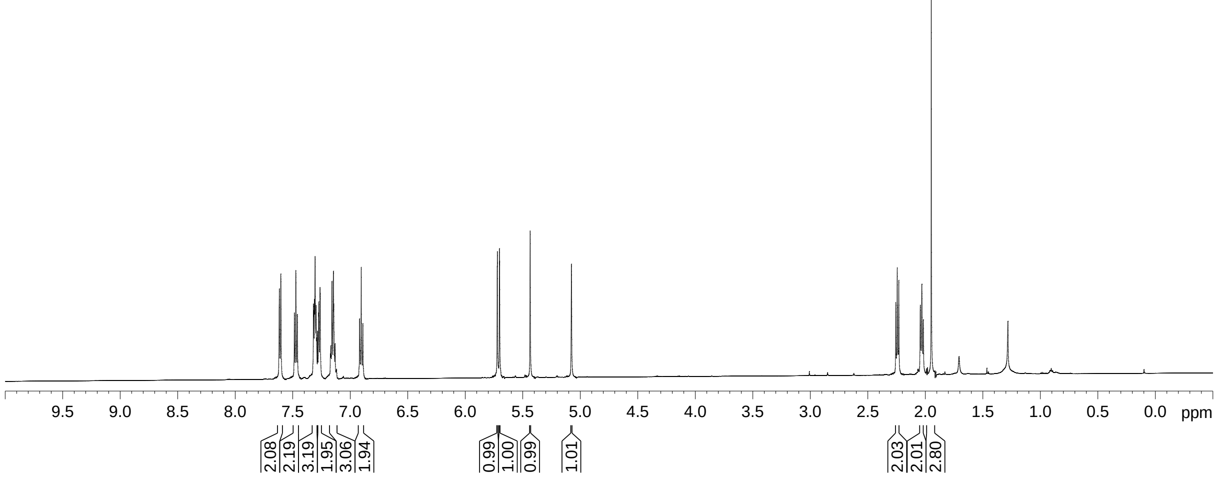

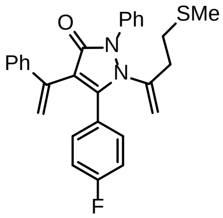

3ah

$CDCl_3$, 150MHz

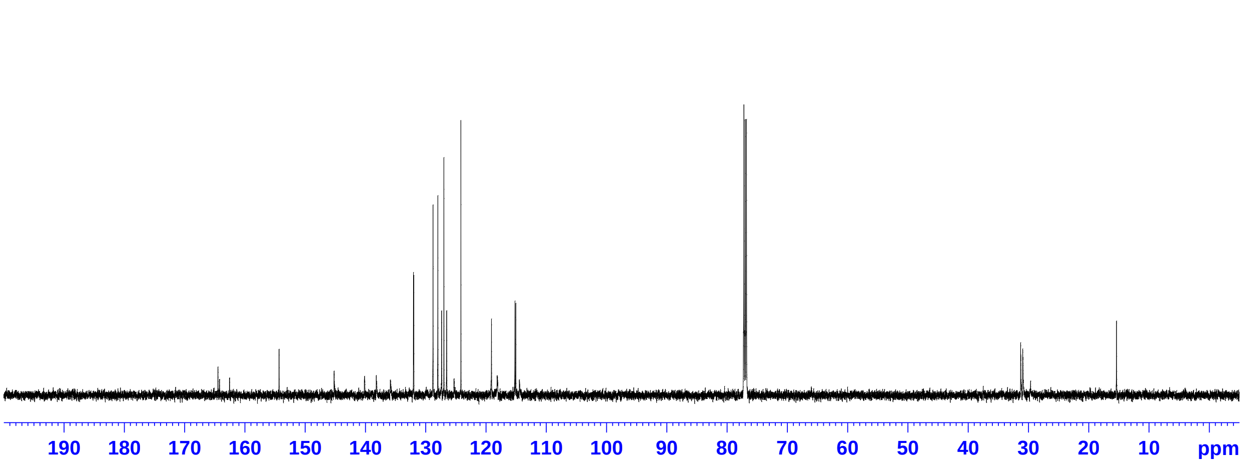

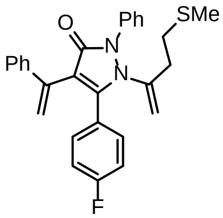

3ah

$CDCl_3$, 600MHz

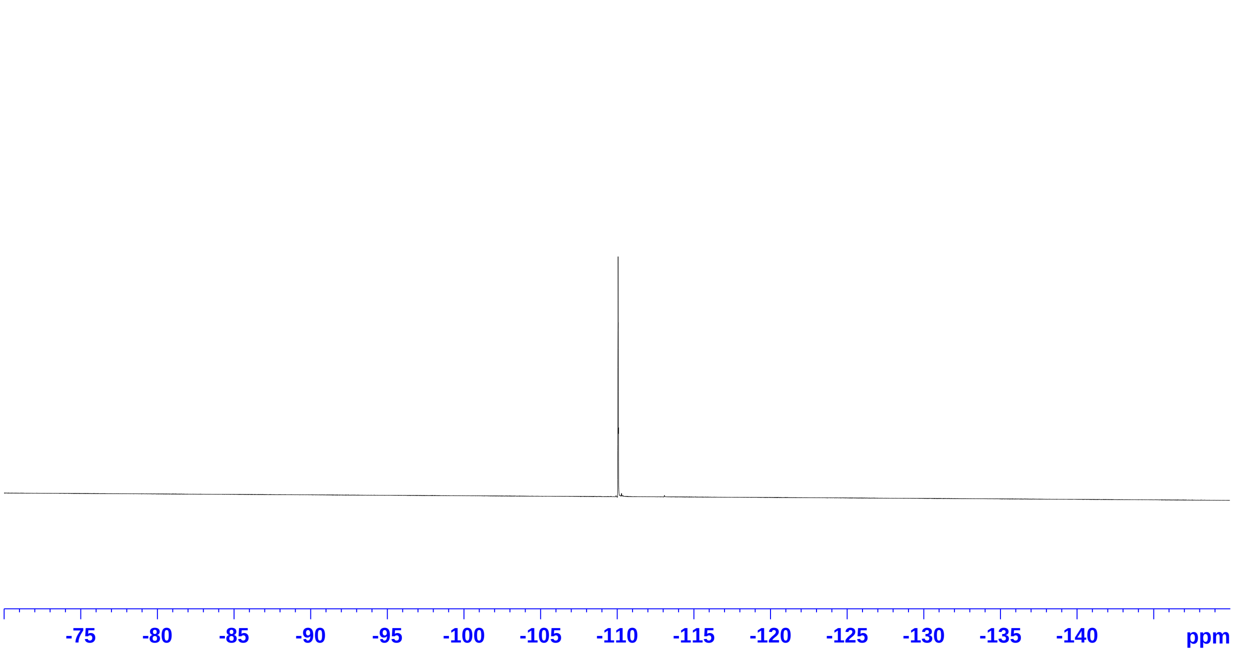

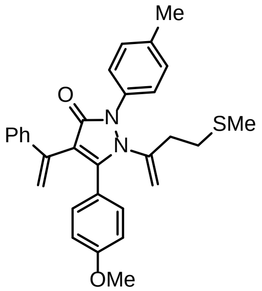

3ai

$CDCl_3$, 600MHz

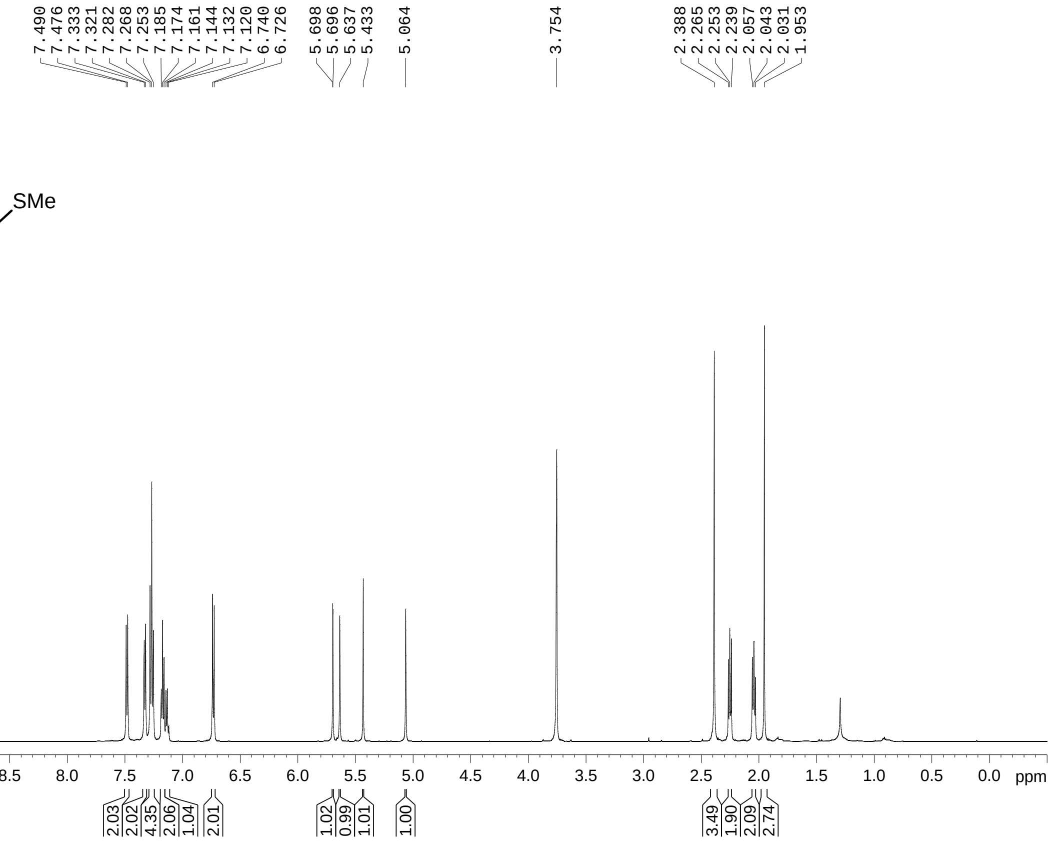

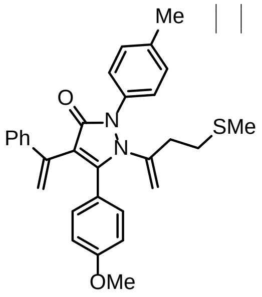

3ai

$CDCl_3$,150MHz

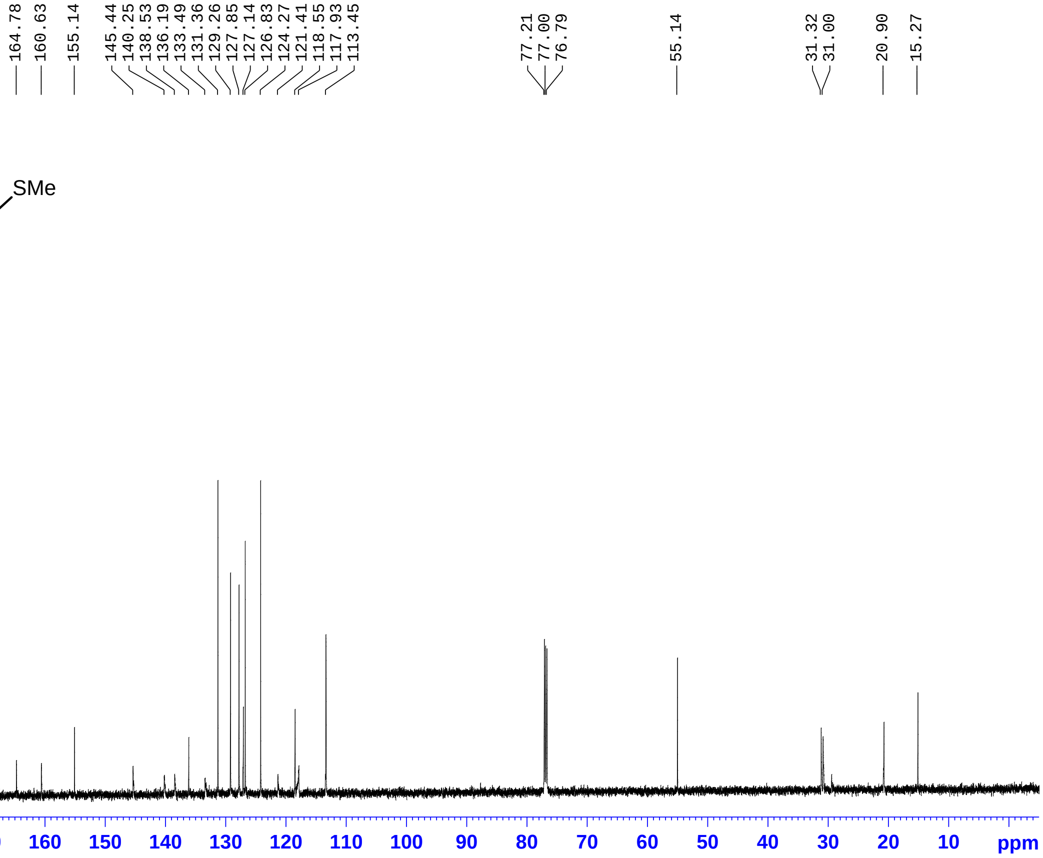

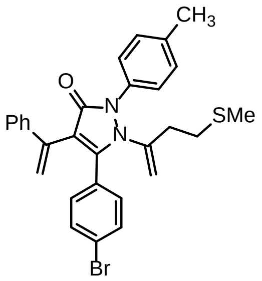

3aj

$CDCl_3$, 600MHz

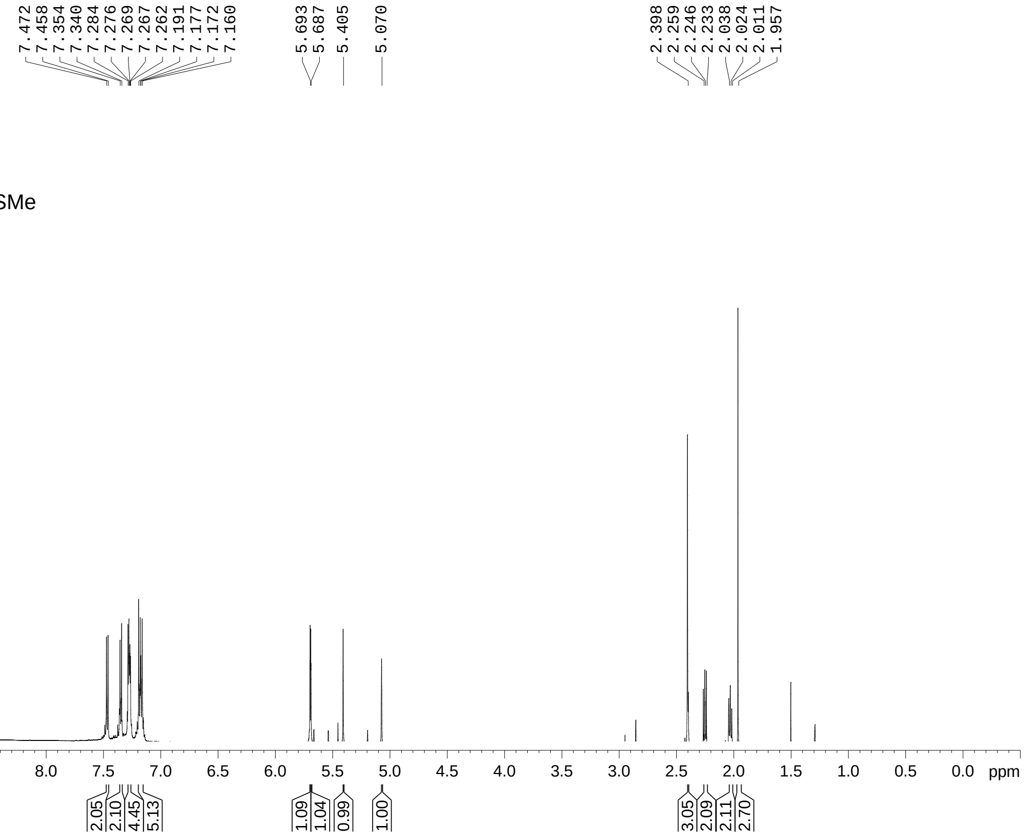

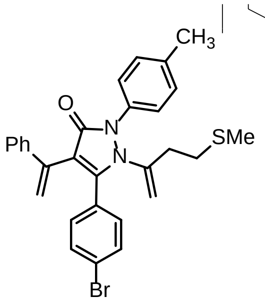

3aj

$CDCl_3$, 150MHz

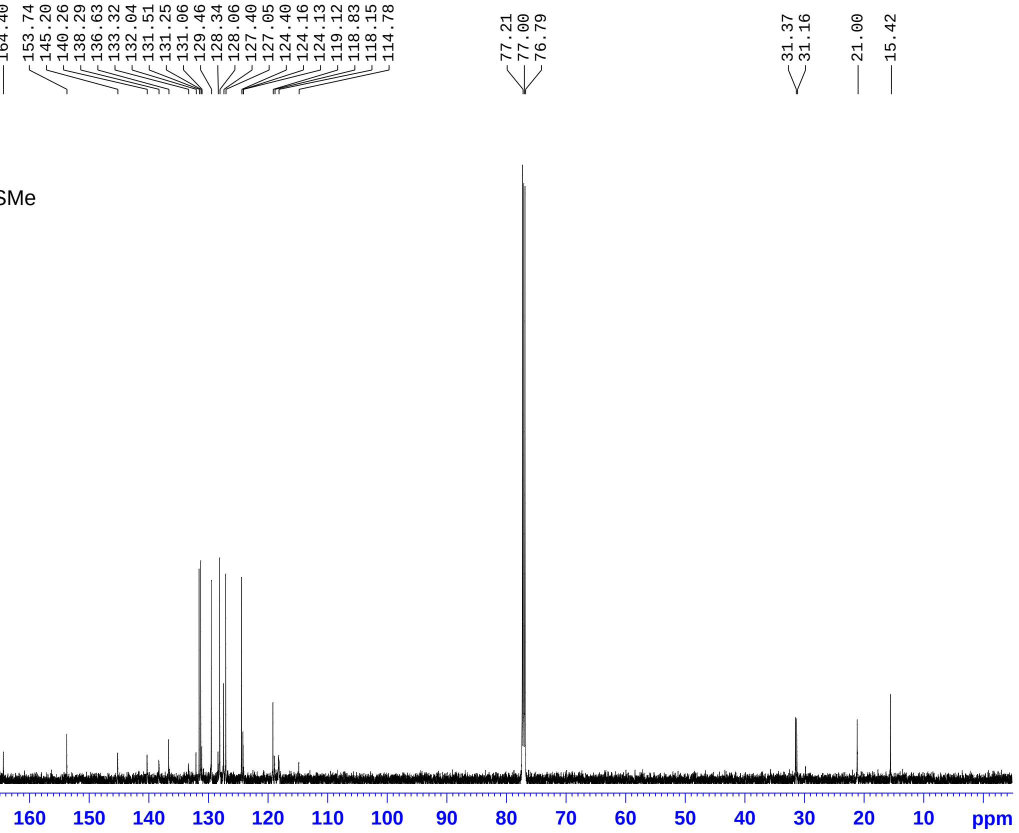

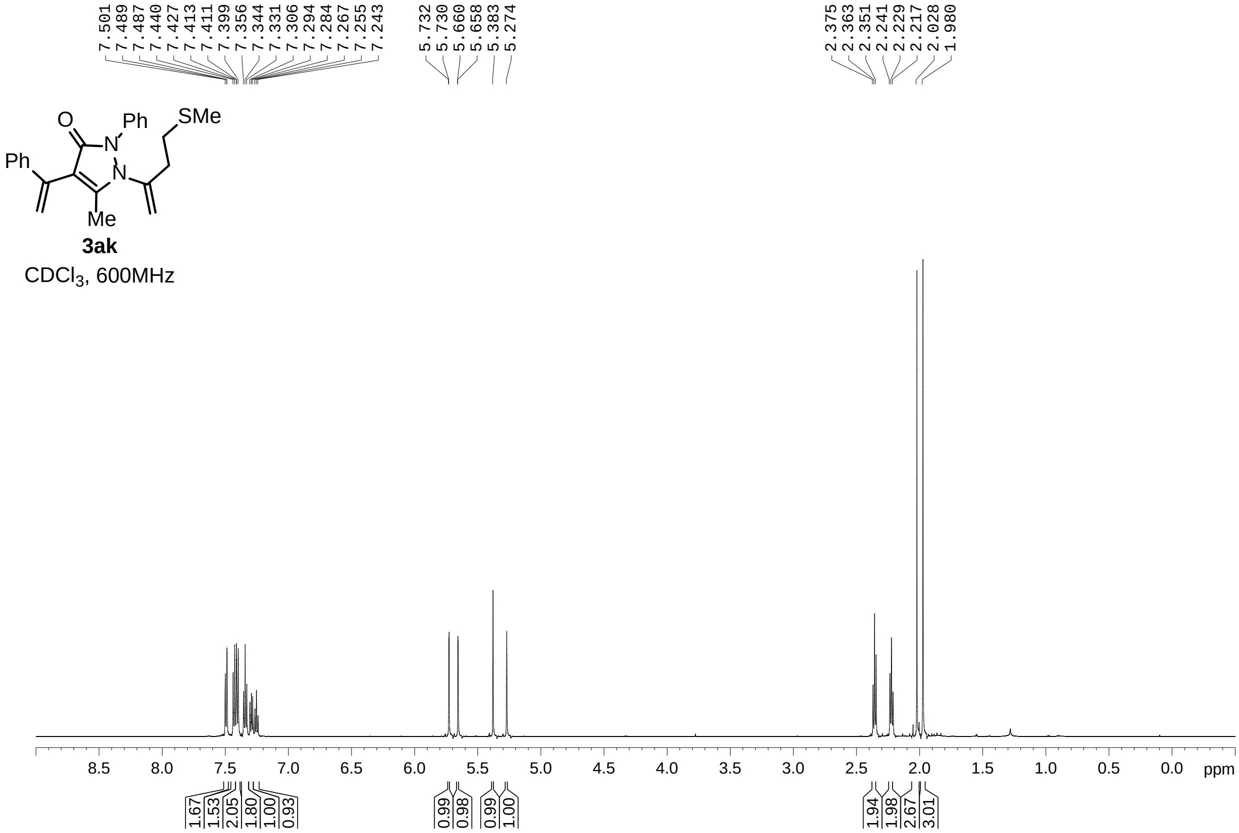
7.501
7.489
7.487
7.440
7.427
7.413
7.411
7.399
7.356
7.344
7.331
7.306
7.294
7.284
7.267
7.255
7.243
5.732
5.730
5.660
5.658
5.383
5.274
2.375
2.363
2.351
2.241
2.229
2.217
2.028
1.980
O
Ph
SMe
N
Ph
N
Me
3ak
CDCl3, 600MHz
1.67
1.53
2.05
1.80
1.00
0.93
0.99
0.98
0.99
1.00
1.94
1.98
2.67
3.01
ppm

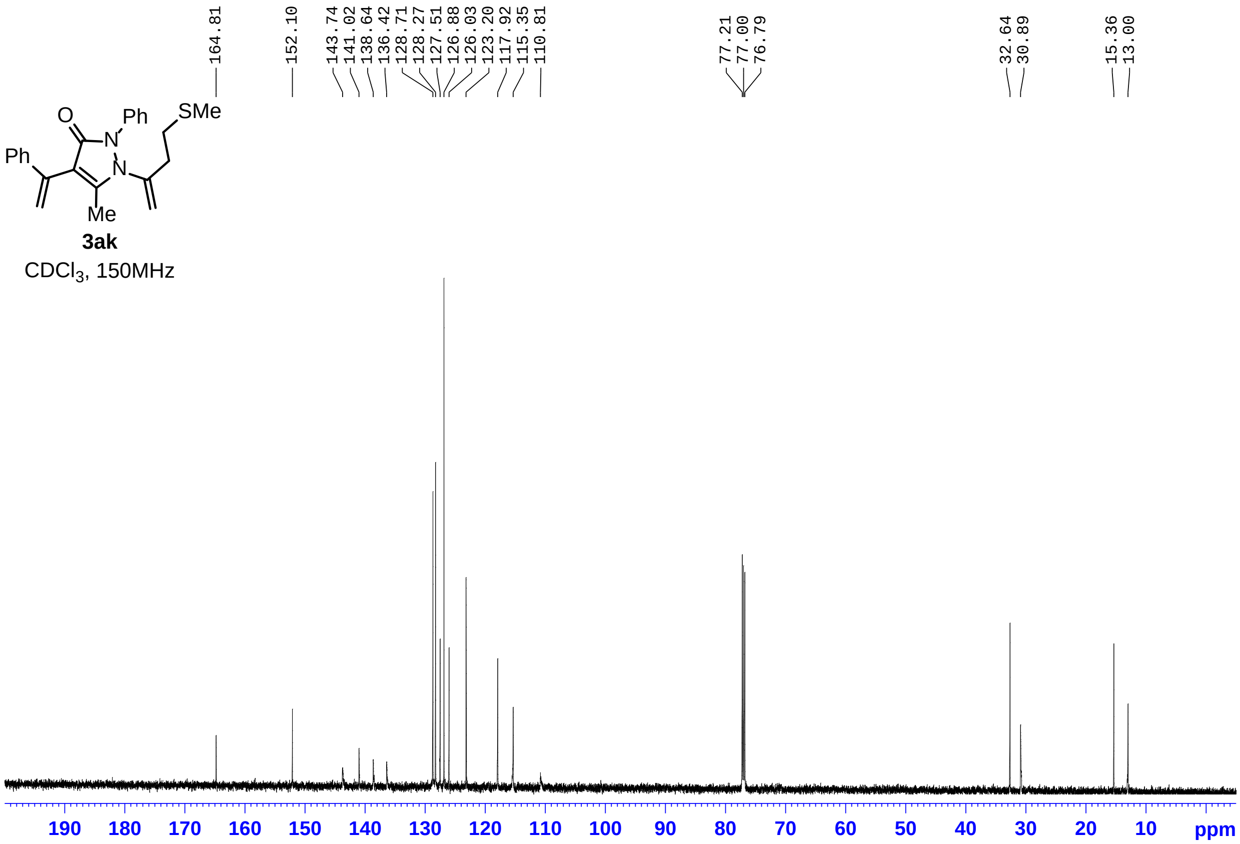
164.81
152.10
143.74
141.02
138.64
136.42
128.71
128.27
127.51
126.88
126.03
123.20
117.92
115.35
110.81
77.21
77.00
76.79
32.64
30.89
15.36
13.00
O
Ph
SMe
N
Ph
N
Me
3ak
CDCl3, 150MHz
ppm

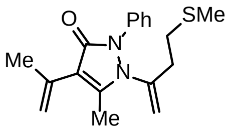

3al

$CDCl_3$, 600MHz

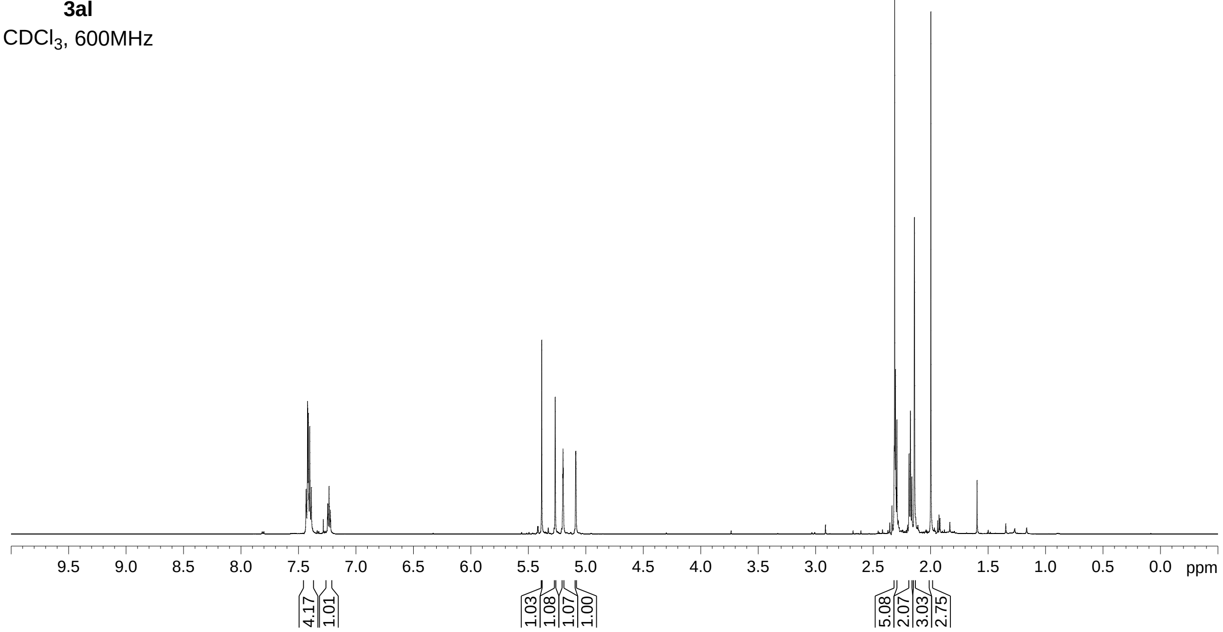

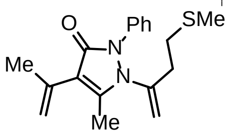

3al

$CDCl_3$, 150MHz

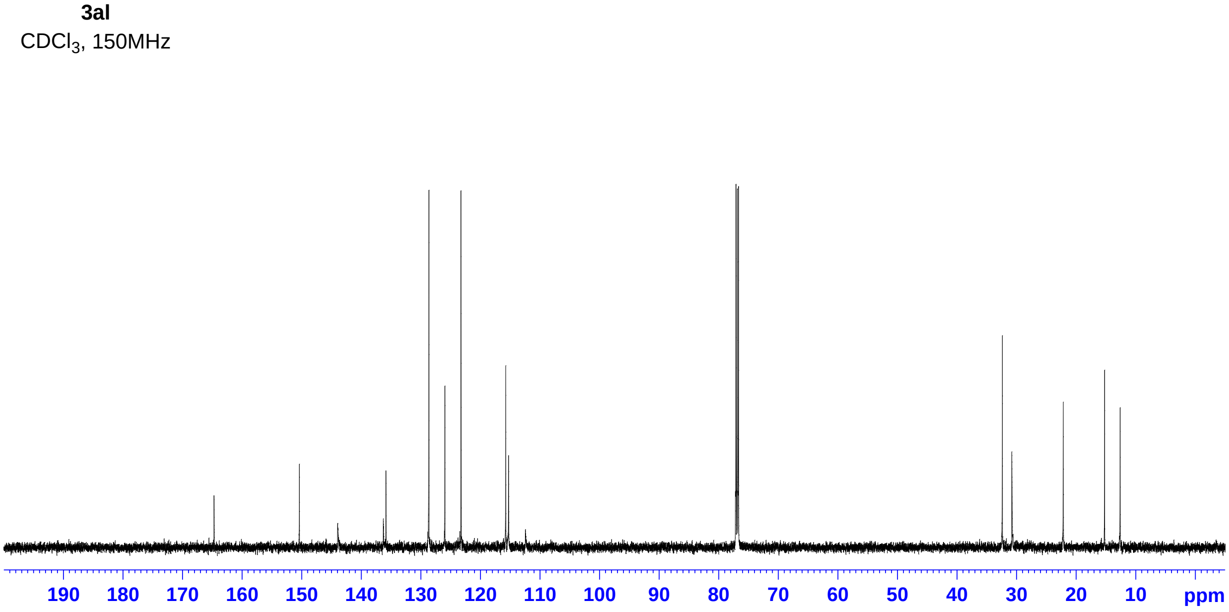

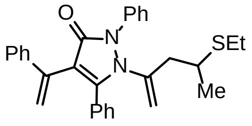
4
CDCl$_3$, 600 MHz

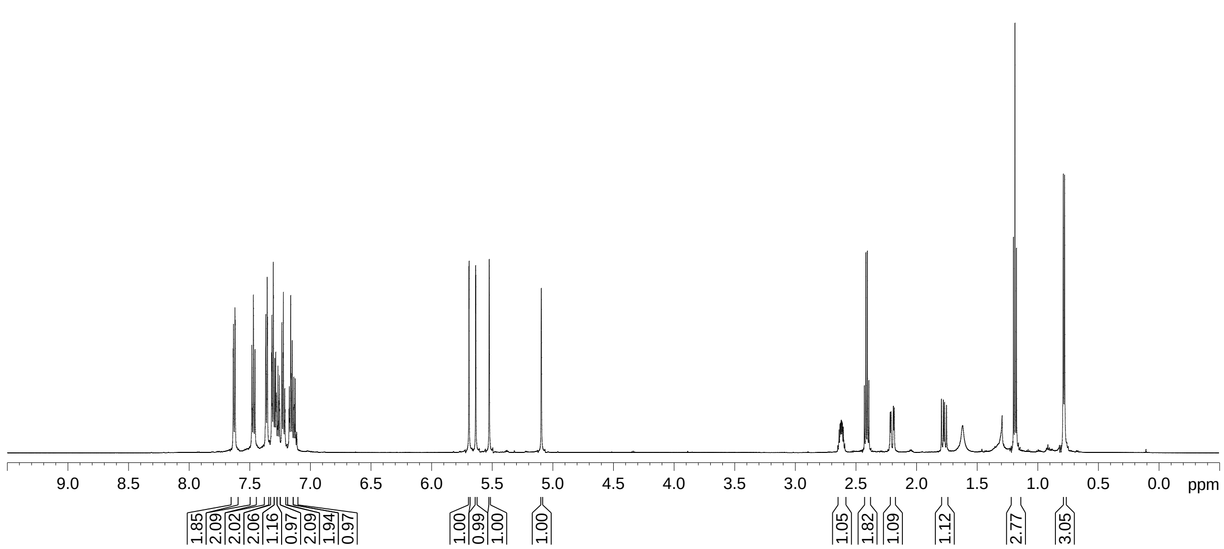

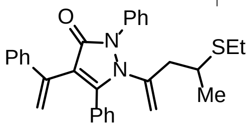
4
CDCl$_3$, 150MHz

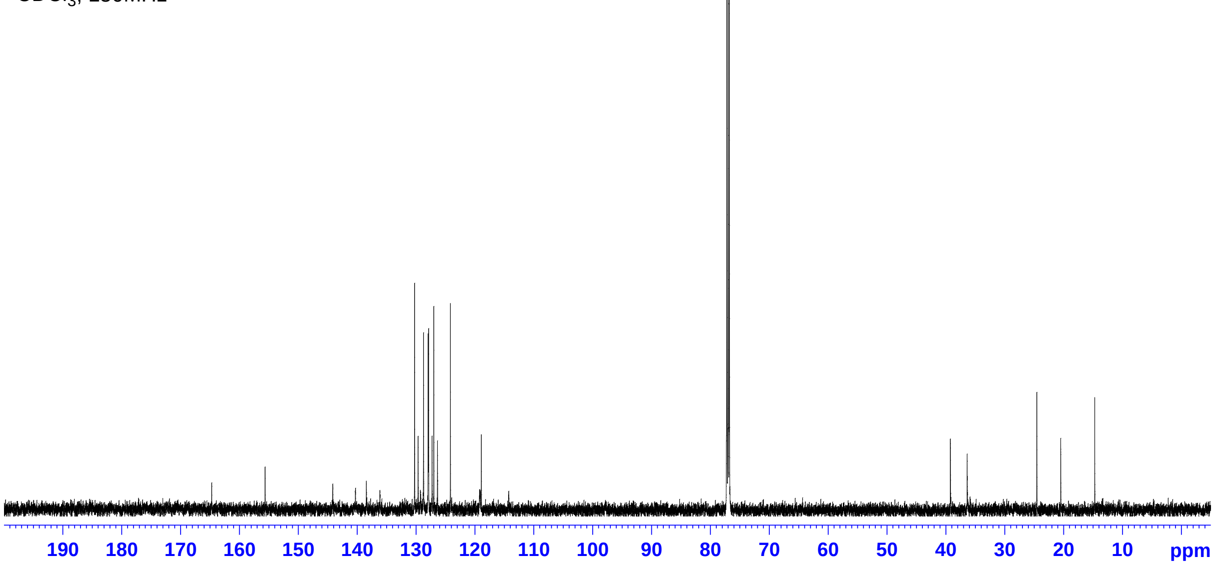

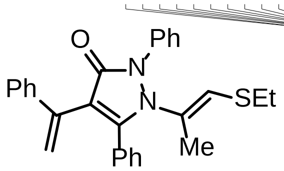

5

$CDCl_3$, 600 MHz

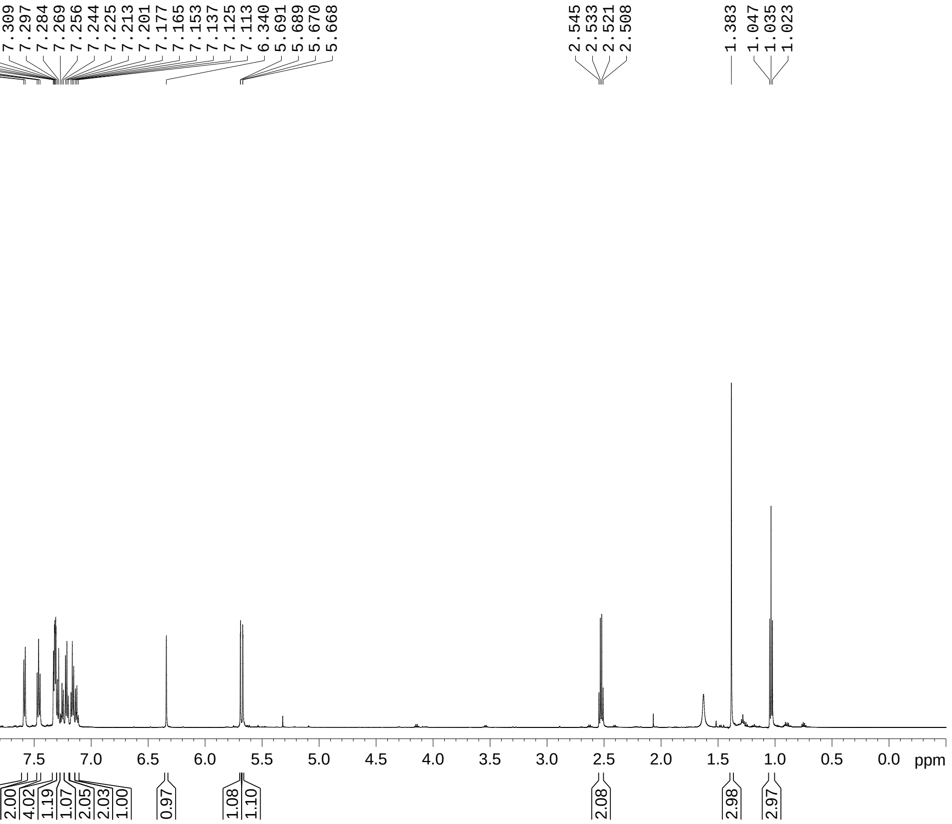

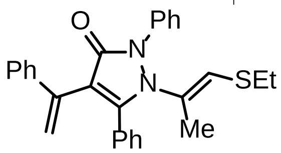

5

$CDCl_3$, 150 MHz

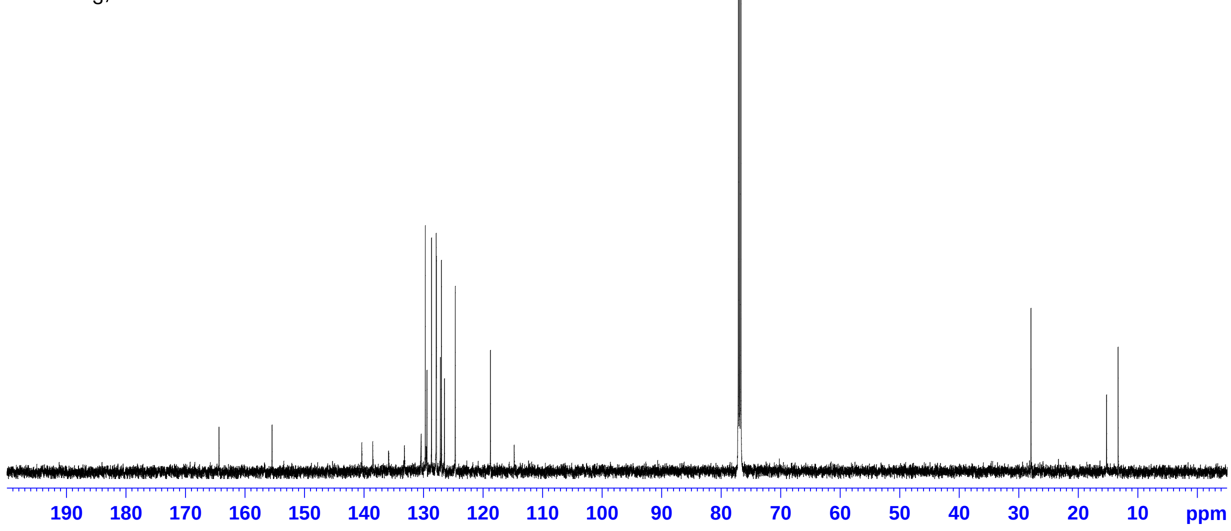

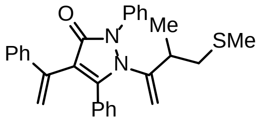

6

$CDCl_3$, 600 MHz

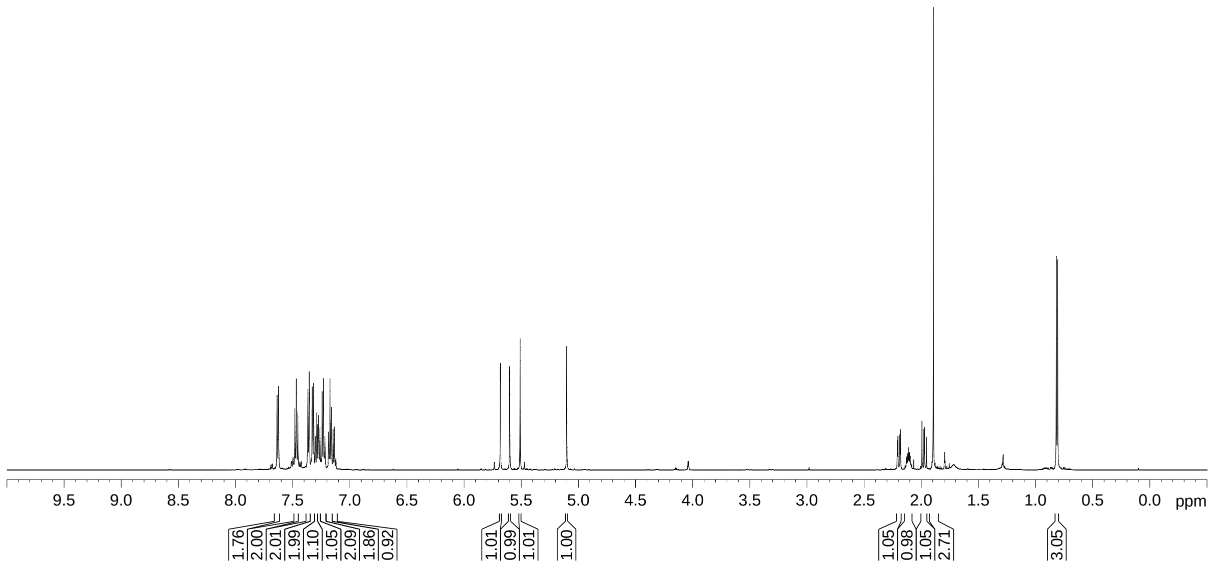

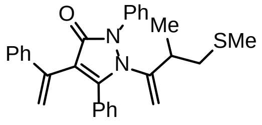

6

$CDCl_3$, 150 MHz

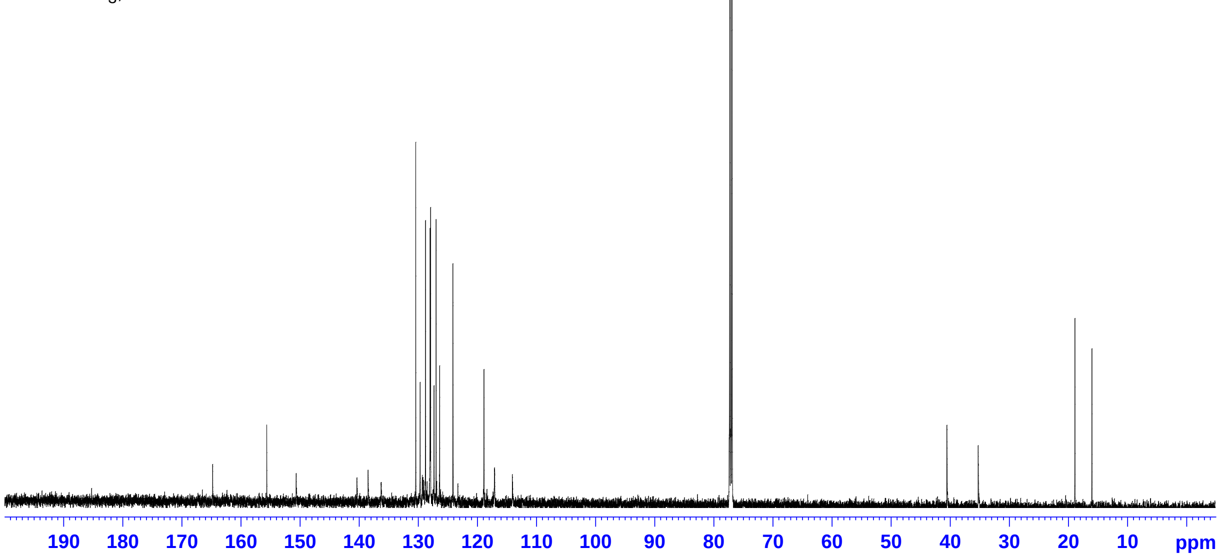

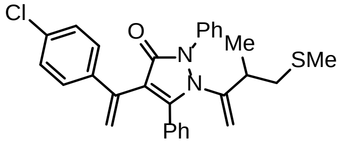

7

$CDCl_3$, 600 MHz

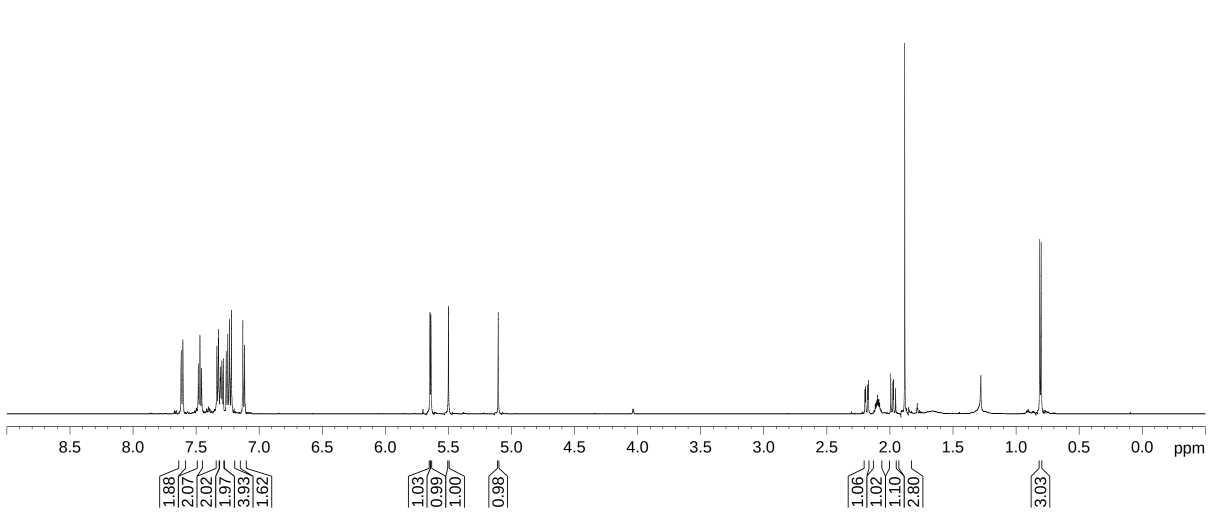

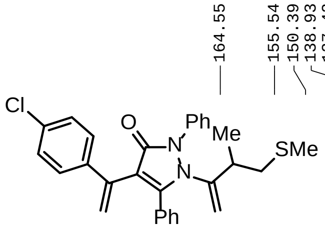

7

$CDCl_3$, 150 MHz

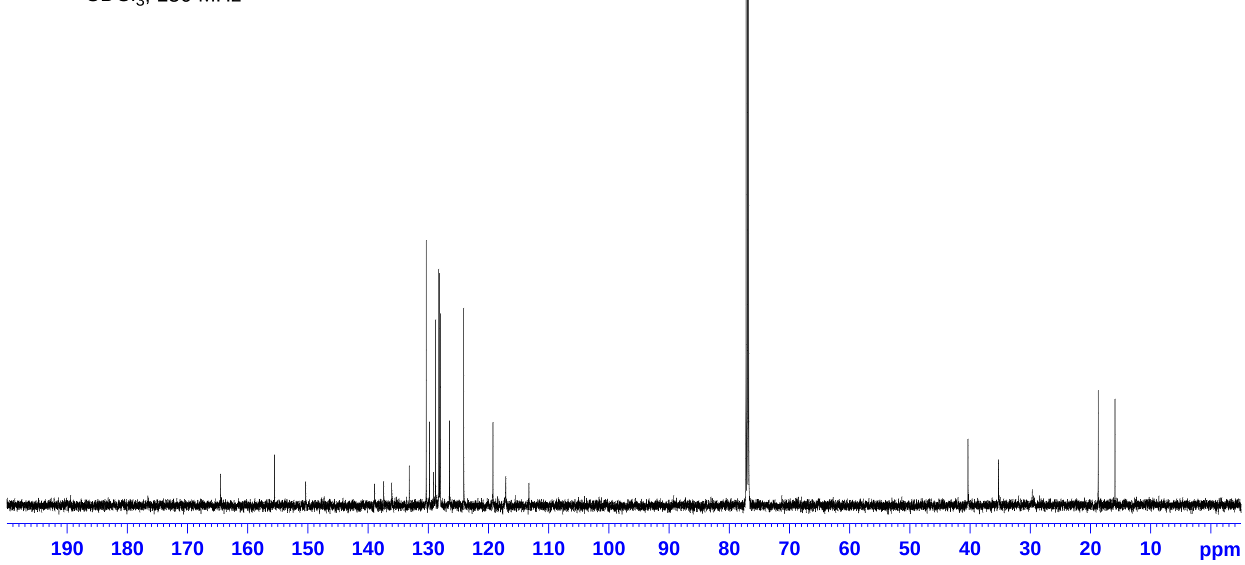

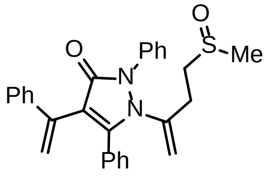

8

$CDCl_3$, 600 MHz

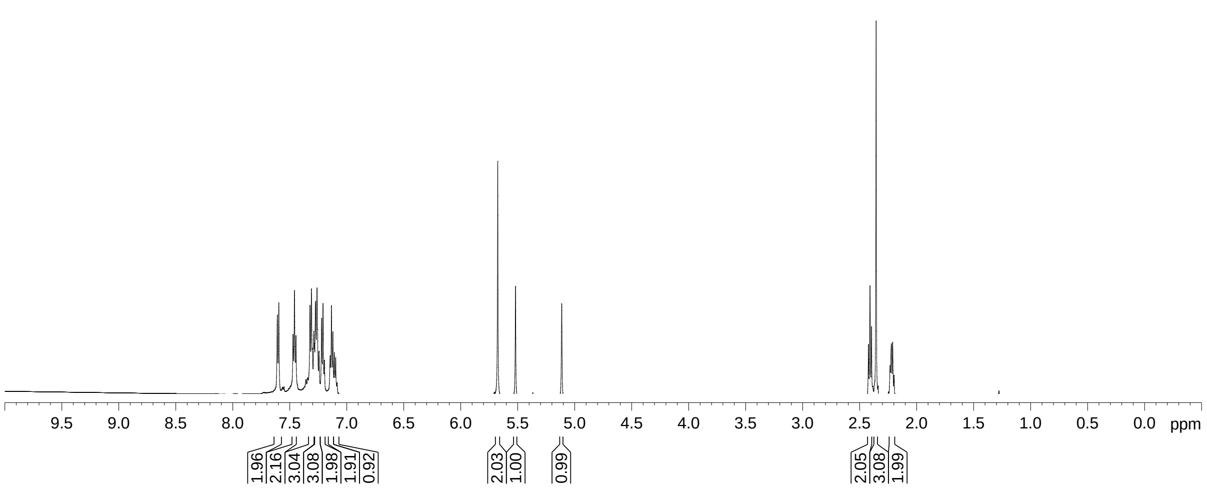

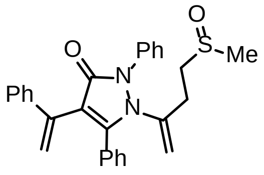

8

$CDCl_3$, 150 MHz

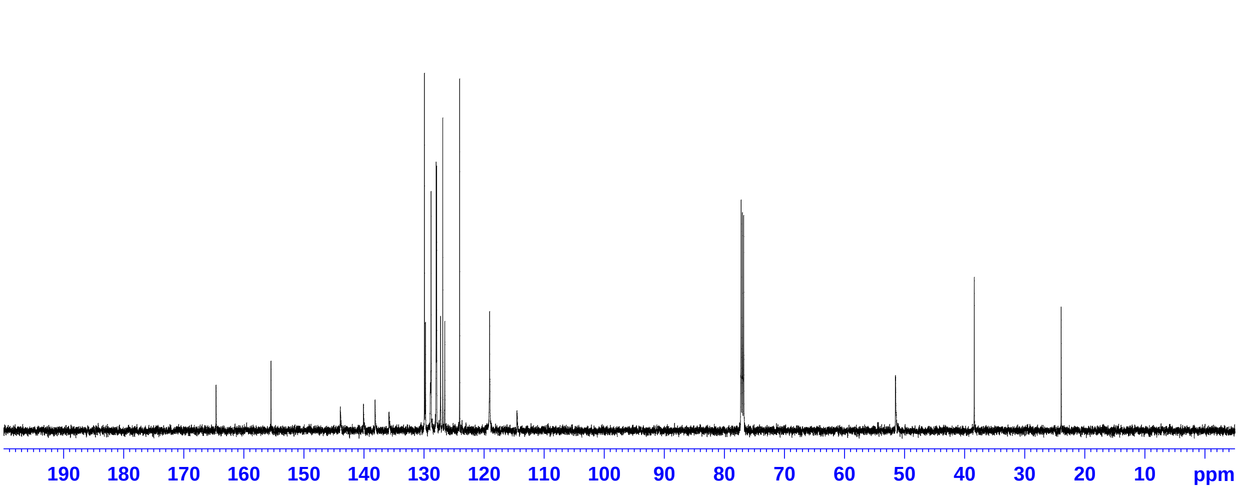

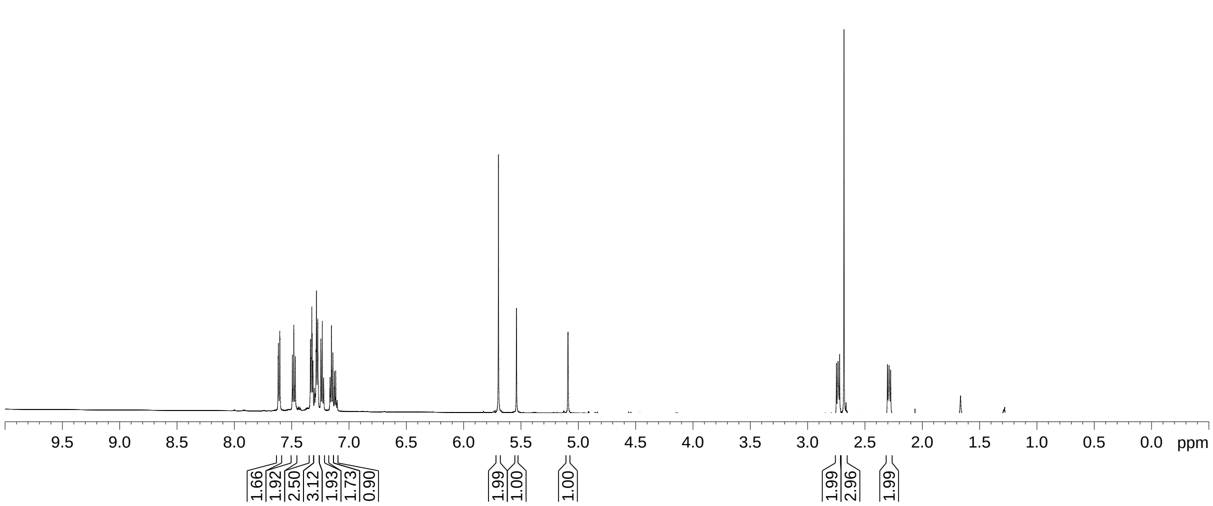

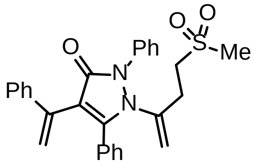

9

$CDCl_3$, 600 MHz

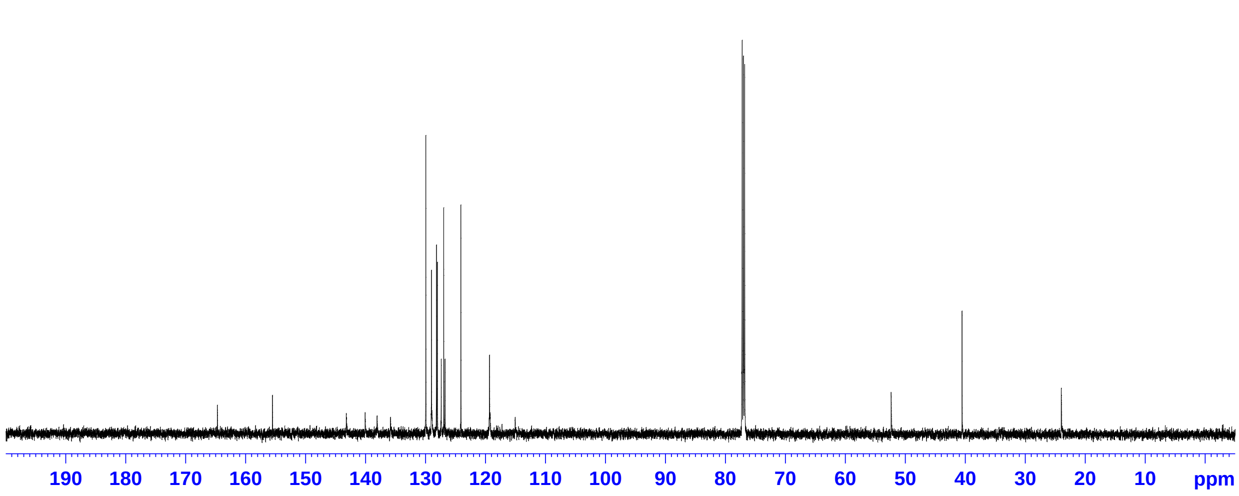

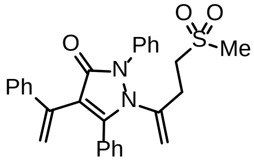

9

$CDCl_3$, 150 MHz
